# Supplementary material for: Recreating pathophysiology of CLN2 disease and demonstrating reversion by TPP1 gene therapy in hiPSC-derived retinal organoids and retina-on-chip
Source: Cell Rep Med. 2025 Jul 23;6(8):102244. doi: 10.1016/j.xcrm.2025.102244 (PMC12432371; doi:10.1016/j.xcrm.2025.102244)
Supplement: Document S1. Figures S1–S7 and Tables S1–S3 [file mmc1.pdf]

Cell Reports Medicine, Volume *6*

Supplemental information

Recreating pathophysiology of CLN2 disease and demonstrating reversion by TPP1 gene therapy in hiPSC-derived retinal organoids and retina-on-chip

Serena Corti, Kwi Hye Kim, Ting Chen, Adelina Botezatu, Virginia Cora, Ke Ma, Natalia Pashkovskaia, Anamaria Bernal Vergara, Denise Sperlich, Kaushambee Dave, Arianna Tolone, Ryan M. Reddinger, Christopher B. Tully, Mikayla Higgins, Alexander Kleger, Markus Breunig, Paul Lopatta, Svenja Wingerter, Madalena Cipriano, Sylvia Bolz, Marius Ueffing, Nicholas Buss, Peter Loskill, Stefan Liebau, and Kevin Achberger

Supplemental Information- Recreating pathophysiology of CLN2 disease and demonstrating reversion by TPP1 gene therapy in hiPSCs-derived retinal organoid and retina-on-chip

Serena Corti[1*], Kwi Hye Kim[2*], Ting Chen[2], Adelina Botezatu[3,4], Virginia Cora[1], Ke Ma[1], Natalia Pashkovskaia[1], Anamaria Bernal Vergara[1], Denise Sperlich[1], Kaushambee Dave[1], Arianna Tolone[1], Ryan Reddinger[2], Christopher Tully[2], Mikayla Higgins[2], Alexander Kleger[6], Markus Breunig[6], Paul Lopatta[6], Svenja Wingerter[6], Madalena Cipriano[6], Sylvia Bolz[7], Marius Ueffing[7], Nicholas Buss[2], Peter Loskill[6,8], Stefan Liebau[1], Kevin Achberger[1†]

[1]Institute of Neuroanatomy & Developmental Biology (INDB), Eberhard Karls University Tübingen, Tübingen, Germany

[2]REGENXBIO Inc. Rockville, MD, USA

[3]German Cancer Consortium (DKTK), partner site Dresden, Germany

[4]German Cancer Research Center (DKFZ), Heidelberg, Germany

[5]Institute for Molecular Oncology and Stem Cell Biology, Ulm University Hospital, Ulm, Germany

[6]Institute of Biomedical Engineering, Eberhard Karls University Tübingen, Tübingen, Germany

[7]Centre for Ophthalmology, Institute for Ophthalmic Research, Eberhard Karls University Tübingen, Tübingen, Germany

[8]NMI Natural and Medical Sciences Institute at the University of Tübingen, Reutlingen, Germany

*Authors contributed equally

† Lead contact: kevin.achberger@uni-tuebingen.de

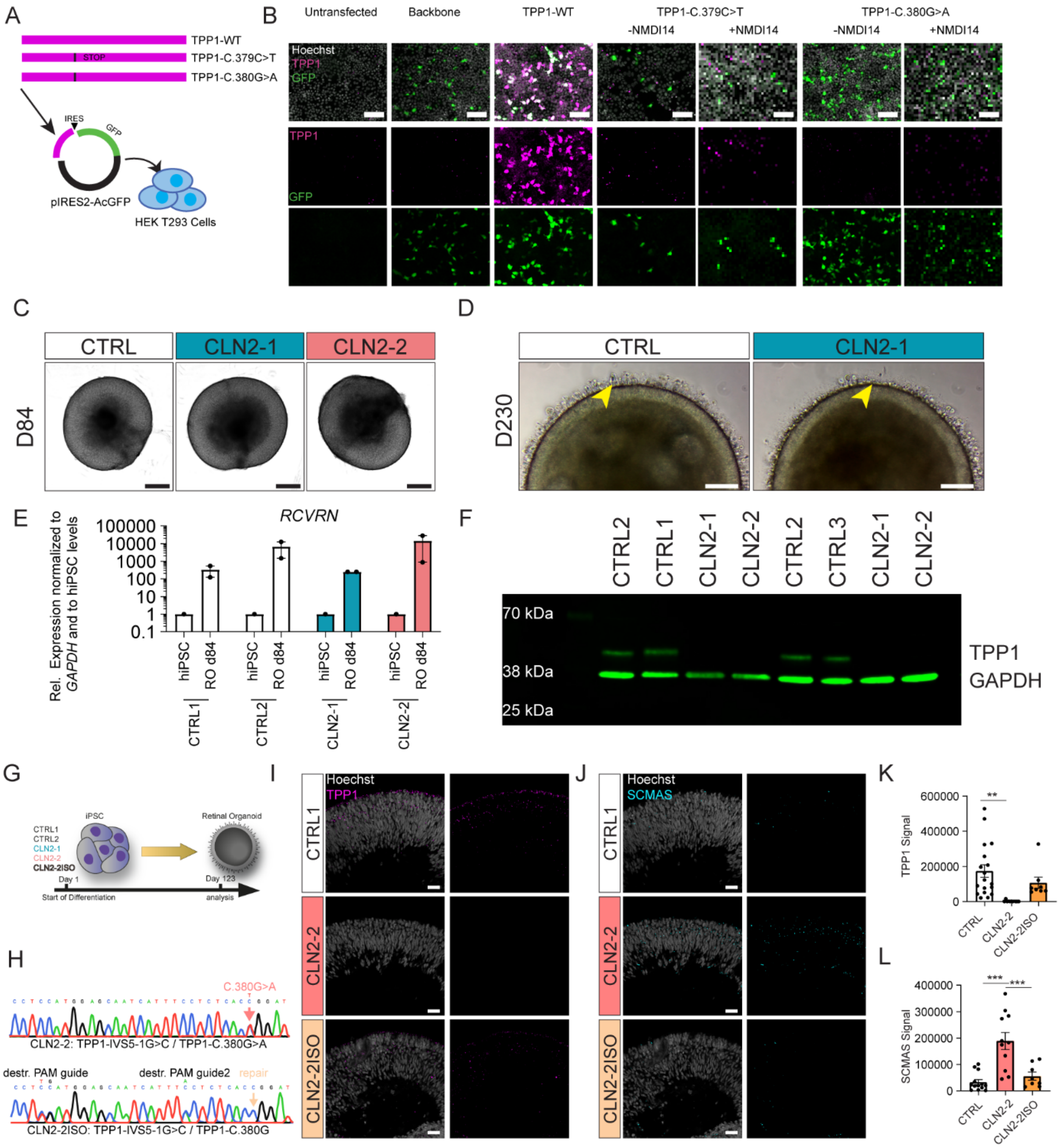

Fig. S1: Characterization of *TPP1* mutations and CLN2 Retinal Organoids. Related to Fig.1 and Fig.2

A) Experimental procedure: *TPP1*-wildtype (WT), *TPP1*-c.379C>T and *TPP1*-c380G>A cDNA was cloned into a pIRES2.AcGFP expression vector and then expressed in HEK T293 cells. B) *TPP1* cDNA variants were expressed for 24h in HEK T293 cells with or without 1 µM of nonsense mediated inhibitor NMDI14 and then stained for GFP (green) and for TPP1 (magenta). C) Brightfield image of CTRL1, CLN2-1 and CLN2-2 ROs at day 84 of differentiation. Scale bar = 200 µm. D) Brightfield image of photoreceptor outer segments (yellow arrowhead) in CTRL1 and CLN2-1 ROs at day 230 of differentiation. Scale bar = 100 µm. E) *RCVRN* (Recoverin) gene expression in ROs at day 84 of differentiation in comparison to hiPSC levels measured by qPCR. Values were normalized to the housekeeping gene GAPDH and then normalized to hiPSC levels. Y-axis is on log 10 scale. N= 1 hiPSC and 2 ROs from one differentiation. F) Western Blot analysis of TPP1 protein expression in CTRL1, CTRL2, CLN2-1, and CLN2-2 ROs at day 350. Samples from the first four and the last four lanes belong to two independent differentiation experiments. G) Creation of isogenic controls. H) Sanger Sequencing trace of mutated

(top) and corrected (bottom) allele in CLN-2ISO. I , J) TPP1 (magenta, I) and SCMAS (cyan, J) immunostaining of CTRL1, CLN2-2, and CLN2-2ISO ROs at day 123 of differentiation. Nuclei were counterstained with Hoechst (gray). Scale bar = 25 µm. K, L) TPP1 (K) and SCMAS (L) fluorescent intensity quantification in CTRL, CLN2-2 and CLN2-2ISO ROs at day 123. Values are mean ± SEM. N= 8-13 ROs from two differentiations. Statistical significance was evaluated by ordinary one-way ANOVA (***$p < 0.001$; ****$p < 0.0001$)

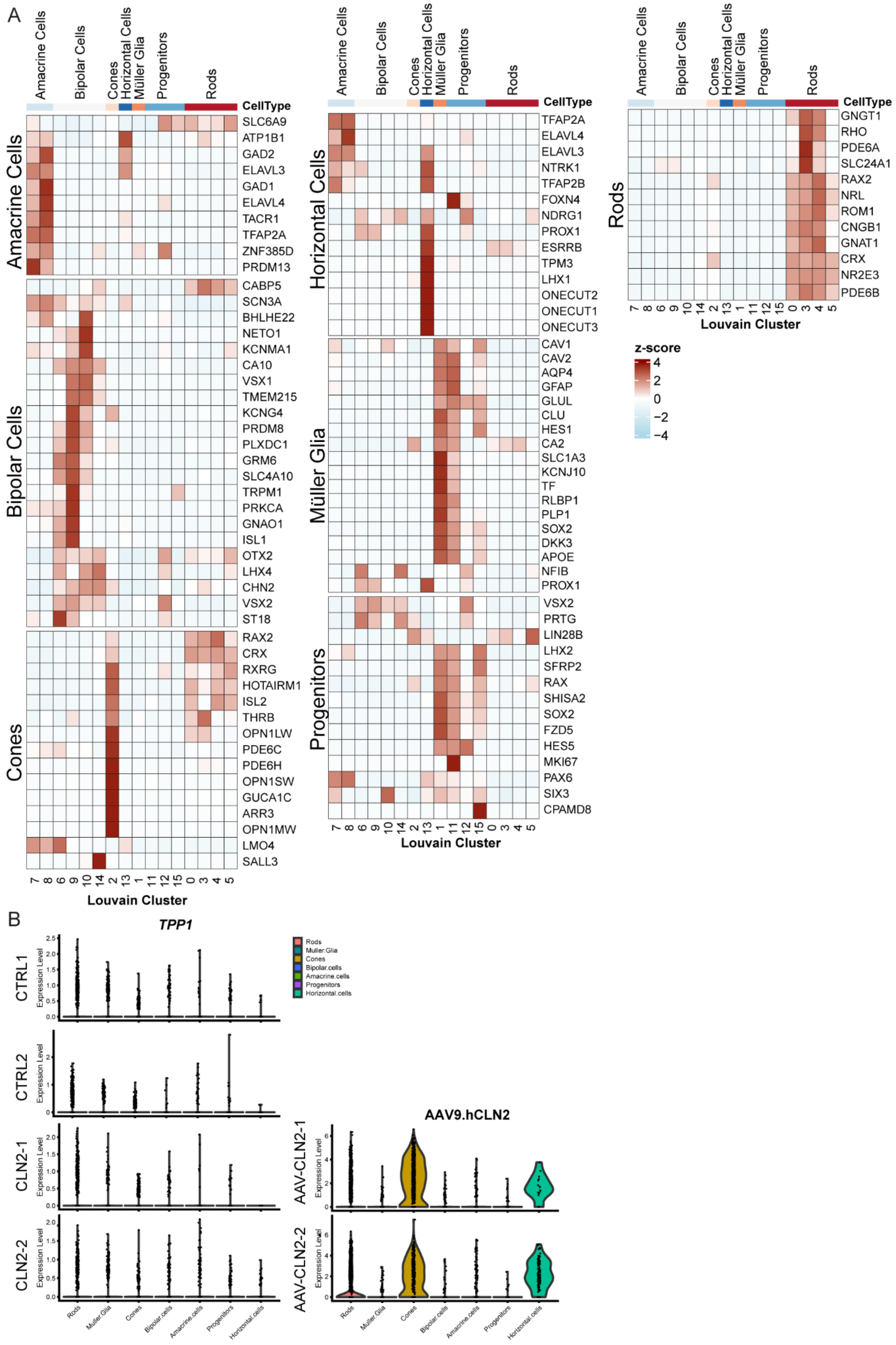
A
Amacrine Cells
Bipolar Cells
Cones
Horizontal Cells
Müller Glia
Progenitors
Rods
CellType
SLC6A9
ATP1B1
GAD2
ELAVL3
GAD1
ELAVL4
TACR1
TFAP2A
ZNF385D
PRDM13
CABP5
SCN3A
BHLHE22
NETO1
KCNMA1
CA10
VSX1
TMEM215
KCNG4
PRDM8
PLXDC1
GRM6
SLC4A10
TRPM1
PRKCA
GNAO1
ISL1
OTX2
LHX4
CHN2
VSX2
ST18
RAX2
CRX
RXRG
HOTAIRM1
ISL2
THRB
OPN1LW
PDE6C
PDE6H
OPN1SW
GUCA1C
ARR3
OPN1MW
LMO4
SALL3
7 8 6 9 10 14 2 13 1 11 12 15 0 3 4 5
Louvain Cluster
TFAP2A
ELAVL4
ELAVL3
NTRK1
TFAP2B
FOXN4
NDRG1
PROX1
ESRRB
TPM3
LHX1
ONECUT2
ONECUT1
ONECUT3
CAV1
CAV2
AQP4
GFAP
GLUL
CLU
HES1
CA2
SLC1A3
KCNJ10
TF
RLBP1
PLP1
SOX2
DKK3
APOE
NFIB
PROX1
VSX2
PRTG
LIN28B
LHX2
SFRP2
RAX
SHISA2
SOX2
FZD5
HES5
MKI67
PAX6
SIX3
CPAMD8
GNGT1
RHO
PDE6A
SLC24A1
RAX2
NRL
ROM1
CNGB1
GNAT1
CRX
NR2E3
PDE6B
z-score
4
2
0
−2
−4
B
TPP1
CTRL1
CTRL2
CLN2-1
CLN2-2
Expression Level
Rods
Muller.Glia
Cones
Bipolar.cells
Amacrine.cells
Progenitors
Horizontal.cells
AAV9.hCLN2
AAV-CLN2-1
AAV-CLN2-2

Fig. S2. Retinal cell type identity, TPP1 and AAV9.hCLN2 transgene expression in CTRL and CLN2 ROs. Related to Fig.1

(A) Heatmap of a single cell RNA sequencing dataset of day 192 ROs (CTRL, CLN2 and AAV9.hCLN2-treated CLN2 ROs combined) depicting marker gene expression of 7 different retinal cell types (Amacrine Cells, Bipolar Cells, Cones, Horizontal Cells, Müller Glia, Retinal Progenitors and Rods) in unsupervised Louvain clusters (0-15). Values are depicted as scaled z-score. Top row indicates cell type label that was selected for each Louvain cluster. (B) Violin plots of a single-cell RNA sequencing dataset of day 192 ROs (CTRL1, CTRL2, CLN2-1, CLN2-2 and AAV9.hCLN2-treated CLN2 ROs) depicting *TPP1* gene and AAV9.hCLN2 transgene expression in 7 different retinal cell types (Rods, Müller Glia, Cones, Bipolar Cells, Amacrine Cells, Retinal Progenitors, and Horizontal Cells).

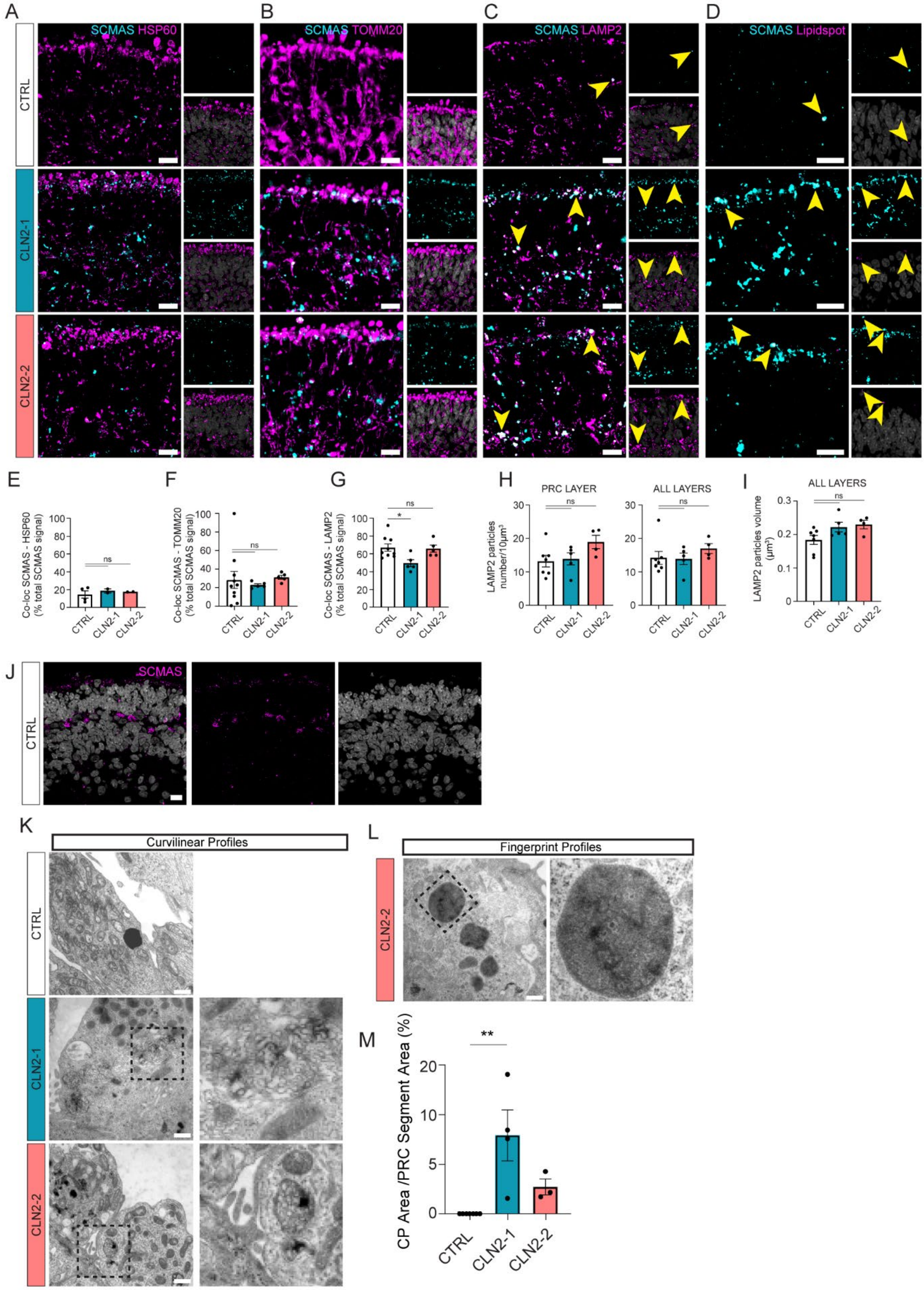

A
B
C
D
SCMAS HSP60
SCMAS TOMM20
SCMAS LAMP2
SCMAS Lipidspot
CTRL
CLN2-1
CLN2-2
E
F
G
H
I
Co-loc SCMAS - HSP60 (% total SCMAS signal)
Co-loc SCMAS - TOMM20 (% total SCMAS signal)
Co-loc SCMAS - LAMP2 (% total SCMAS signal)
LAMP2 particles number/10µm³
LAMP2 particles volume (µm³)
PRC LAYER
ALL LAYERS
ns
*
J
SCMAS
K
Curvilinear Profiles
L
Fingerprint Profiles
M
CP Area /PRC Segment Area (%)
**

Fig. S3. Subcellular localization analysis of SCMAS punctae and ultrastructural evaluation of CLN2 depositions . Related to Fig.2

A-D) Single confocal plane showing co-localization of SCMAS (cyan) with HSP60 (magenta, A), TOMM20 (magenta, B) LAMP2 (magenta, C) and LipidSpot (magenta, D) in CTRL1 and CLN2 ROs at day 200 of differentiation. Nuclei were counterstained with Hoechst (gray). Scale bar = 10 µm. Examples of colocalizing signal are indicated with yellow arrowheads. E-G) Co-localization percentage of SCMAS with HSP60 (E), TOMM20 (F) and LAMP2 (G). Values indicate proportion of SCMAS signal that co-localizes with either HSP60 or LAMP2, respectively. N= 2-5 ROs from one differentiation. H and I) Quantification of LAMP2+ particles per 10 µm3 in photoreceptor cell (PRC) layer (H, left graph) and all retinal layers (H, right graph) and LAMP2+ particle volume (I) in CTRL and CLN2 ROs at day 200. Values are mean ± SEM. N=5 ROs from one differentiation. Statistical significance was evaluated by ordinary one-way ANOVA (*p < 0.05). J) Single confocal plane showing SCMAS expression visualized with increased brightness in a CTRL RO at day 158. K) Transmission electron microscopy images of d300 ROs showing curvilinear profiles (CP) in CLN2-1 and CLN2-2 photoreceptor (PRC) segments. The magnification of dashed squares is shown in the right column. Scale bar = 500nm. L) Transmission electron microscopy images of d300 CLN2-2 ROs showing fingerprint deposits in photoreceptor segments. The magnification of the dashed square is shown in the right column. Scale bar = 500nm. M) Percentage of photoreceptor segment area covered with curvilinear profiles in CTRL, CLN2-1 and CLN2-1 d300 ROs. Values are mean ± SEM. N= 3-5 ROs from one differentiation. Statistical significance was evaluated by ordinary One-Way ANOVA test (**p < 0.01)

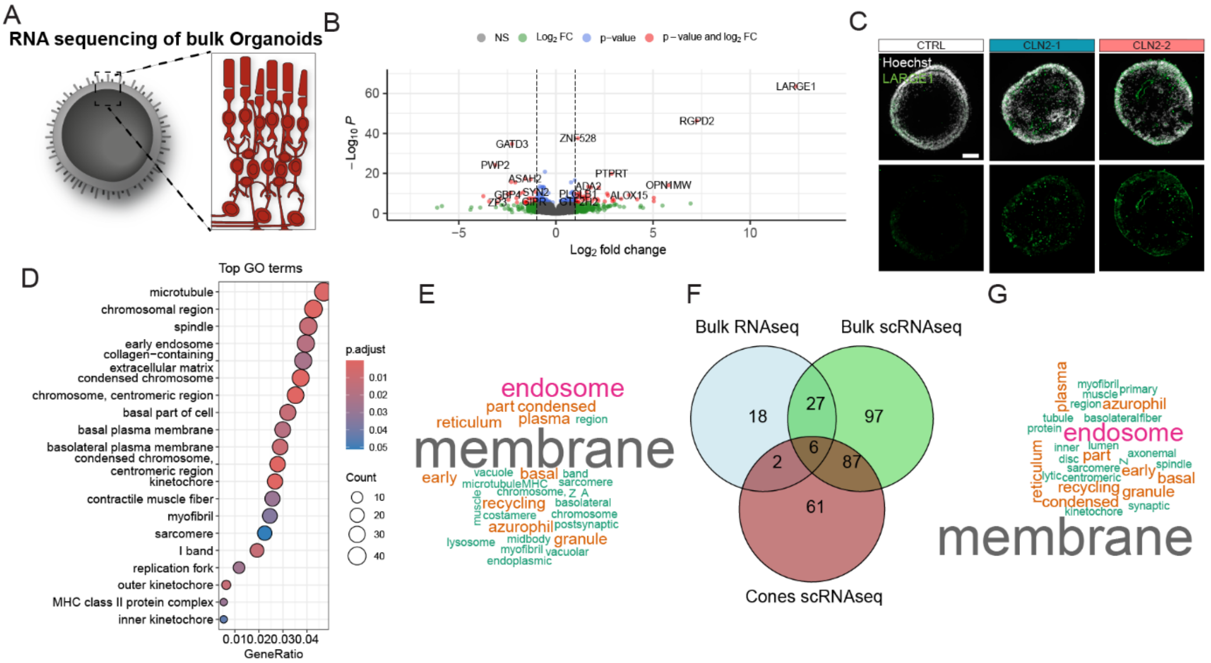

Fig. S4. Bulk RNA sequencing analysis. Related to Fig.3

A) RNA sequencing was performed with bulk d200 ROs. B) Vulcano Plot showing up- and downregulated genes comparing CLN2 (CLN2-1 and CLN2-2) and CTRLs (CTRL1 and CTRL2). Positive Log2 fold change values depict upregulation. C) Immunofluorescent staining showing LARGE1 (green) in CTRL2 and CLN2 organoids at day 321 of differentiation. Nuclei were counterstained with Hoechst (gray). Scale bar = 100 µm. D) Dot Plot of Gene Ontology (GO) Analysis showing the 20 top GO terms amongst dysregulated genes in CLN2 ROs in comparison to CTRLs. E) Word Cloud of semantic terms that were most mentioned in significantly enriched GO terms comparing CLN2 and CTRL ROs. F) Venn Diagram of GO Terms that have been found when using DGE of Bulk RNAseq, (Pseudo) bulk single cell RNAseq and scRNAseq cone data for the analysis. G) Word Cloud of semantic terms that are shared between bulk RNAseq and bulk scRNAseq analysis.

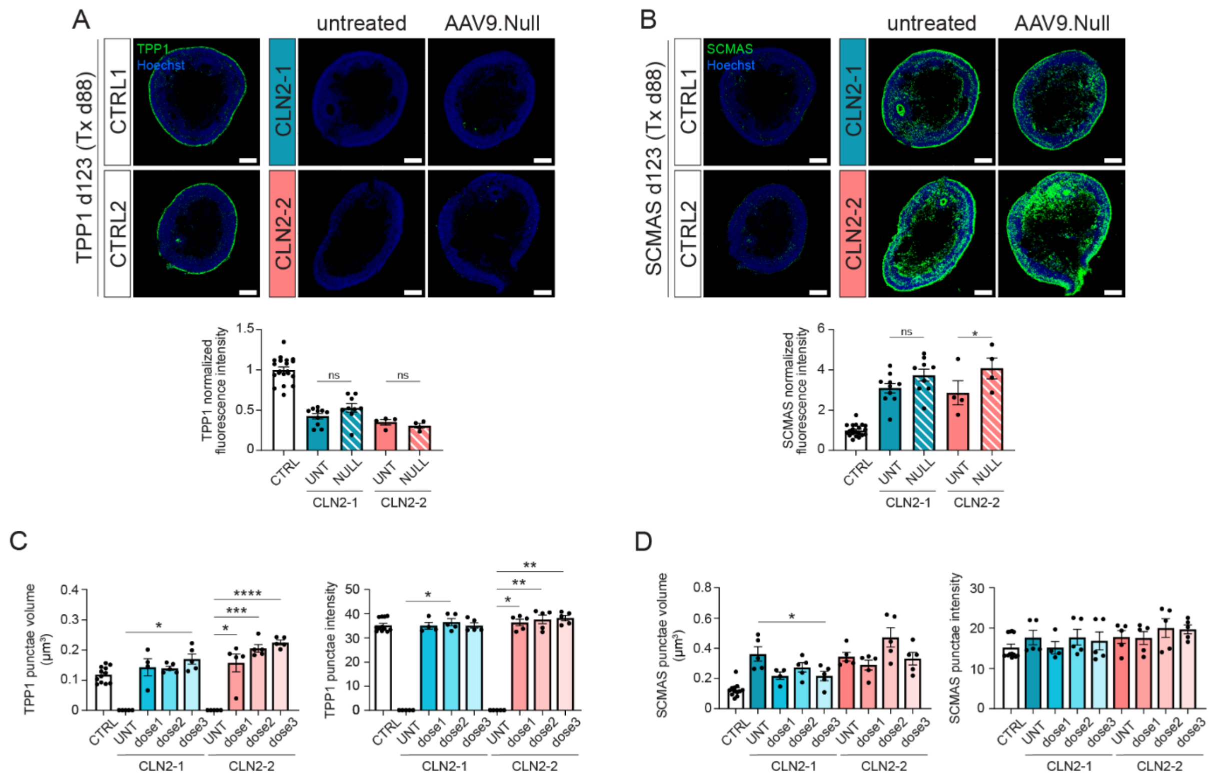

Fig. S5. Characterization of CLN2 ROs treated with AAV9.Null or AAV9.hCLN2. Related to Fig.4 and Fig.5

A and B) TPP1 (green, A) and SCMAS (green, B) immunostaining of d123 CTRL, untreated (UNT) CLN2 and CLN2 organoids treated with AAV9.Null (NULL) (1.67×10^{11} gc/RO) at day 88. Nuclei were counterstained with Hoechst (blue). Scale bar = 100 µm. TPP1 and SCMAS protein expressions were evaluated by quantification of their fluorescent intensity. Number of analyzed organoids: CLN2-1 n=9-10 from two independent experiments; CLN2-2 n=4 from one experiment; CTRL1 n=8 from one experiment; CTRL2 n=12 from three independent experiments. Values are mean ± SEM. Statistical significance was evaluated by ordinary one-way ANOVA (*$p < 0.05$). Tx: treatment. C and D) Quantification of TPP1 (C) and SCMAS (D) punctae volume (left graphs) and fluorescent intensity (right graphs) in CTRL and CLN2 d158 ROs treated with AAV9.hCLN2 at day 123. Three different doses of AAV9.hCLN2 were tested (dose 1: 5×10^{9}, dose 2: 5×10^{10} and dose 3: 1.67×10^{11} gc/RO). Values are mean ± SEM. N=5 organoids from two independent experiments. Statistical significance was evaluated by ordinary one-way ANOVA (*$p < 0.05$; ***$p < 0.001$; ****$p < 0.0001$)

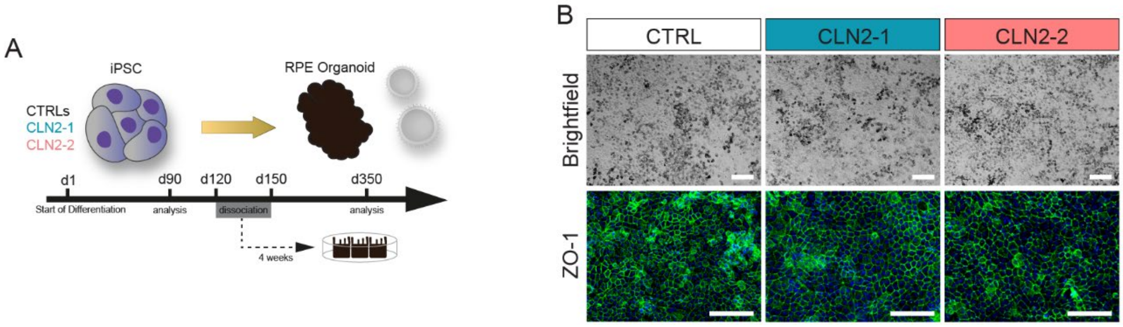

Fig. S6. Characterization of RPE cells. Related to Fig.6

A) Schematic overview of the 2D RPE derived from 3D RPE organoids differentiation protocol and time points used for analyses. B) Brightfield images (top row) and immunostaining of the RPE marker ZO-1 (green, bottom row) of CTRL1 and CLN2 RPE cells cultured in 2D for 4 weeks. Nuclei were counterstained with Hoechst (blue). Scale bar = 100 µm.

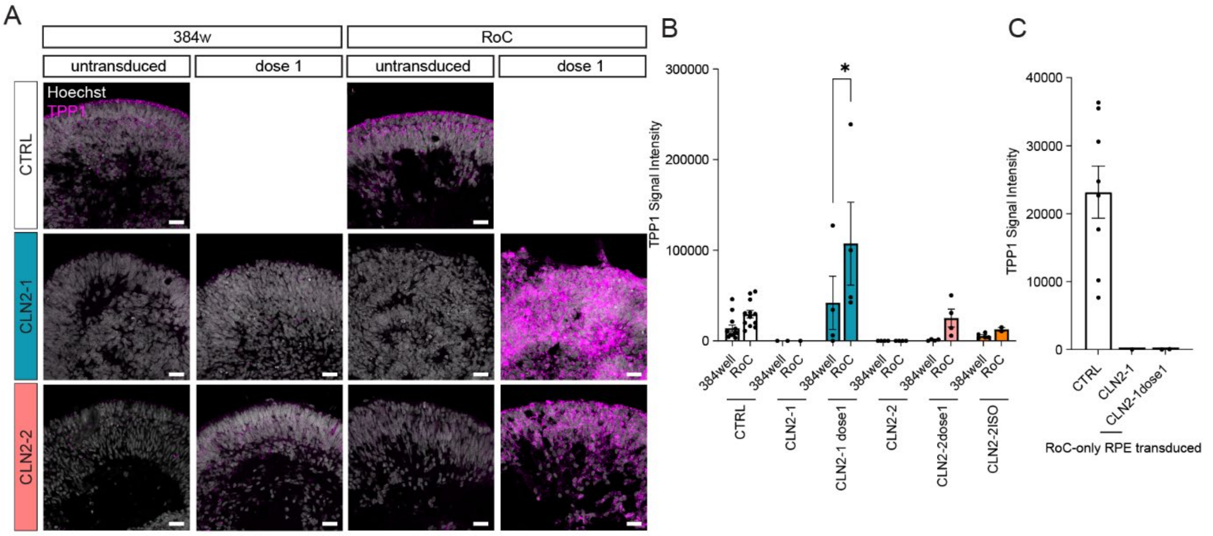

Fig S7: AAV9.hCLN2 is more efficient in the RoC than in 384-well plate. Related to Fig.7

A and B) Comparison of the RO transduced with AAV9-hCLN2 (dose 1) in a 384-well plate and the RoC with similar volumes. A) Exemplary TPP1 (magenta) immunostaining of CTRL1, CLN2-1 and CLN2-2 ROs at day 123 of differentiation comparing 384-well plate and RoC cultures as well as untransduced and AAV9.hCLN2 transduced (dose 1). Nuclei were counterstained with Hoechst (gray). Scale bar = 25 µm. B) TPP1 protein was evaluated by quantification of fluorescent intensity. N=4 from one experiment. Values are mean ± SEM. Statistical significance was evaluated by a two-way ANOVA (**p < 0.01). C) TPP1 protein levels of a RoC, where only the RPE cell were transduced with AAV9-hCLN2 ("RoC-only RPE transduced") at dose 1. Values are mean ± SEM. N=4 from one experiment.

Table S1, related to STAR Methods- Immunohistochemistry

Antibody/dye	Species/type	Dilution	Catalog number	Supplier
CRALBP	Mouse monoclonal	1:250	ab15051	Abcam, UK
Ezrin	Rabbit polyclonal	1:200	3145	Cell Signaling Technology, USA
GAPDH	Mouse monoclonal	1:1000	MAB374	Merck Millipore, USA
GFP	Rabbit polyclonal	1:1000	A6455	Thermo Fisher Scientific, USA
HSP60	Mouse monoclonal	1:1000	ab128567	Abcam, UK
LAMP1	Rabbit polyclonal	1:200	ab24170-100	Abcam, UK
LAMP2	Mouse monoclonal	1:50	Sc-18822	Santa Cruz Biotechnology, USA
LipidSpot™ 488 Lipid Droplet Stain	-	1:1000	70065-T	Biotium, USA
Recoverin	Goat polyclonal	1:50	Sc-20353	Santa Cruz Biotechnology, USA
TPP1	Mouse monoclonal	1:200	Sc-365838	Santa Cruz Biotechnology, USA
SCMAS	Rabbit polyclonal	1:100	ab181243	Abcam, UK
TOMM20	Rabbit polyclonal	1:100	11802-1-AP	Proteintech,USA
TOMM20	Mouse monoclonal	1:200	SC-17764	Santa Cruz Biotechnology, USA

ZO1	Mouse monoclonal	1:100	33-9100	Thermo Fisher Scientific, USA

Table S2, related to STAR Methods- Immunohistochemistry

**Antibody**	**Type**	**Dilution**	**Catalog number**	**Supplier**
Alexa Fluor™ 488	Donkey anti mouse IgG	1:500	ab150105	Abcam, UK
Alexa Fluor™ 488	Donkey anti rabbit IgG	1:500	ab150073	Abcam, UK
Alexa Fluor™ 568	Donkey anti mouse IgG	1:500	ab175472	Abcam, UK
Alexa Fluor™ 568	Donkey anti rabbit IgG	1:500	ab175470	Abcam, UK
Alexa Fluor™ 647	Donkey anti mouse IgG	1:500	ab150107	Abcam, UK
Alexa Fluor™ 647	Donkey anti rabbit IgG	1:500	ab150075	Abcam, UK
Alexa Fluor™ 647	Donkey anti goat IgG	1:500	ab150131	Abcam, UK
Alexa Fluor™ 568	Donkey anti rabbit IgG	1:500	A10042	Thermo Fisher Scientific, USA

Table S3, related to STAR Methods- CRISPR/Cas9 correction of TPP1 mutation

	**Sequence**
sgRNA	GAGAGGAAAUGAUUGCUCCA
Repair Template	ccatccatctcactgatgggatgactggtgccctTGatggagcaatcatttActctcacCGgatgctcagccagcaagtcagaaagtcctgtgtgatcac
Fwd primer for sequencing	ACATGGGTTTCCGTAGGTCC
Rvrs primer for sequencing	TGCCTCTTCTGAATCCCTGC
