# Supplementary material for: Recreating pathophysiology of CLN2 disease and demonstrating reversion by TPP1 gene therapy in hiPSC-derived retinal organoids and retina-on-chip
Source: Cell Rep Med. 2025 Jul 23;6(8):102244. doi: 10.1016/j.xcrm.2025.102244 (PMC12432371; doi:10.1016/j.xcrm.2025.102244)
Supplement: Document S2. Article plus supplemental information [file mmc2.pdf]

Cell Reports Medicine

Article

Recreating pathophysiology of CLN2 disease and demonstrating reversion by TPP1 gene therapy in hiPSC-derived retinal organoids and retina-on-chip

Graphical abstract

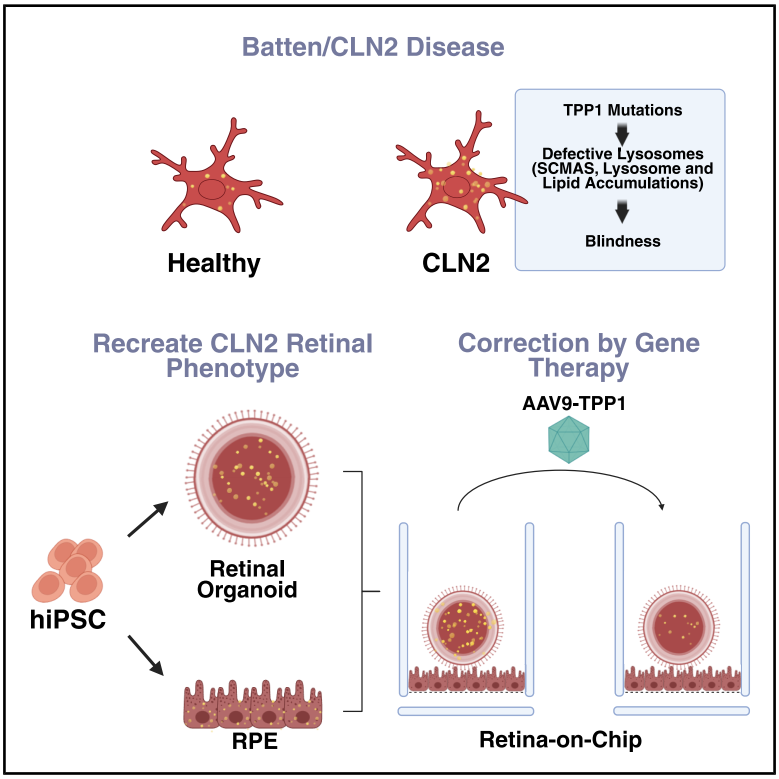

Authors

Serena Corti, Kwi Hye Kim, Ting Chen, ..., Peter Loskill, Stefan Liebau, Kevin Achberger

Correspondence

kevin.achberger@uni-tuebingen.de

In brief

Corti et al. leverage patient hiPSC-derived retinal organoids and retina-on-chip systems to replicate key CLN2 retinal pathologies, including lipofuscin accumulation and cone dysfunction. AAV9-mediated TPP1 gene therapy restores enzyme expression and reduces pathological hallmarks, offering promise for treating CLN2-associated blindness.

Highlights

- Use of human iPSC-derived retinal organoids, RPE cells, and retina-on-chip to model CLN2
- Recapitulation of disease hallmarks such as SCMAS, lipid, and lipofuscin accumulation
- Reversal of pathological hallmarks by AAV-based TPP1 gene therapy

Corti et al., 2025, Cell Reports Medicine 6, 102244
August 19, 2025 © 2025 The Authors. Published by Elsevier Inc.
https://doi.org/10.1016/j.xcrm.2025.102244

CellPress

Cell Reports Medicine

CellPress
OPEN ACCESS

Article

Recreating pathophysiology of CLN2 disease and demonstrating reversion by TPP1 gene therapy in hiPSC-derived retinal organoids and retina-on-chip

Serena Corti,[1,9] Kwi Hye Kim,[2,9] Ting Chen,[2] Adelina Botezatu,[1] Virginia Cora,[1] Ke Ma,[1] Natalia Pashkovskaia,[1] Anamaria Bernal Vergara,[1] Denise Sperlich,[1] Kaushambee Dave,[1] Arianna Tolone,[1] Ryan M. Reddinger,[2] Christopher B. Tully,[2] Mikayla Higgins,[2] Alexander Kleger,[3,4,5] Markus Breunig,[3] Paul Lopatta,[3] Svenja Wingerter,[6] Madalena Cipriano,[6] Sylvia Bolz,[7] Marius Ueffing,[7] Nicholas Buss,[2] Peter Loskill,[6,8] Stefan Liebau,[1] and Kevin Achberger[1,10,*]

[1]Institute of Neuroanatomy & Developmental Biology (INDB), Eberhard Karls University Tübingen, Tübingen, Germany
[2]REGENXBIO Inc, Rockville, MD, USA
[3]Institute of Molecular Oncology and Stem Cell Biology, Ulm University Hospital, Ulm, Germany
[4]Division of Interdisciplinary Pancreatology, Department of Internal Medicine I, Ulm University Hospital, Ulm, Germany
[5]Core Facility Organoids, Ulm University, Ulm, Germany
[6]Institute of Biomedical Engineering, Eberhard Karls University Tübingen, Tübingen, Germany
[7]Centre for Ophthalmology, Institute for Ophthalmic Research, Eberhard Karls University Tübingen, Tübingen, Germany
[8]NMI Natural and Medical Sciences Institute at the University of Tübingen, Reutlingen, Germany
[9]These authors contributed equally
[10]Lead contact
*Correspondence: kevin.achberger@uni-tuebingen.de
https://doi.org/10.1016/j.xcrm.2025.102244

SUMMARY

Mutations in the tripeptidyl peptidase 1 (*TPP1*) gene lead to neuronal ceroid lipofuscinosis type 2 (CLN2), characterized by lysosomal accumulation of lipofuscins predominantly in the brain and retina. The ocular phenotype is characterized by outer retinal degeneration that leads to vision loss. Leveraging human induced pluripotent stem cell (hiPSC)-derived retinal organoids (ROs), retinal pigmented epithelial cells, and the retina-on-chip system, we establish an *in vitro* CLN2 model that recreates the principal histological hallmarks, namely the accumulation of subunit C of mitochondrial ATP synthase (SCMAS) and lipids mainly in the outer retina. Furthermore, single-cell RNA sequencing reveals a dysregulation of translational and mitochondrial function in CLN2 cones. Finally, adeno-associated virus (AAV)-mediated TPP1 gene therapy can restore TPP1 expression and decrease and even prevent SCMAS accumulations. Our study uses an innovative human-relevant microphysiological retinal disease models, uncovers previously uncharacterized mechanisms of CLN2 pathophysiology, and demonstrates the potential of AAV9.hCLN2 gene therapy for CLN2 disease, potentially treating patient blindness.

INTRODUCTION

Neuronal ceroid lipofuscinosis type 2 (CLN2), or late infantile neuronal ceroid lipofuscinosis, is one of the most common forms of neuronal ceroid lipofuscinosis (NCL) affecting children and young adults. CLN2 disease is caused by mutations in the tripeptidyl peptidase 1 (*TPP1*) gene, which leads to absence or severe reduction of the lysosomal protein TPP1.[1,2] Deficiency of functional TPP1 leads to failed protein digestion and subsequent lysosomal accumulation of lipofuscins (autofluorescent storage material composed of proteins and lipids) in the brain, retina, and other organs.[3,4] The most abundant protein component of lipofuscins is subunit C of mitochondrial ATP synthase (SCMAS), a direct substrate of TPP1 proteolytic activity.[5] Lipofuscin accumulation eventually results in neuronal and retinal cell loss.[6] Patients with CLN2 disease manifest various symptoms at 2–4 years of age including language delay, vision impairment, seizures, and regression of motor and cognitive abilities that lead to premature death between 7 and 15 years of age.[6,7] Vision loss is hallmarked by lipofuscin accumulation, progressive bilateral outer retinal degeneration, and accelerated retinal thinning between 4 and 6 years of age, which leads to complete vision loss around years 8–10.[8] Enzyme replacement therapy (ERT) with recombinant human TPP1, cerliponase alfa, is the current standard treatment of CLN2 disease.[7,9] Patients undergo biweekly infusion of ERT into a surgically implanted cerebral port. Despite delaying the decline of motor and language skills and extending lifespan, ERT fails to prevent retinal degeneration and vision loss.[10]

Mutations in the *Tpp1* gene in a CLN2 mouse model were associated with the absence of TPP1 protein and recapitulated neurological symptoms including seizures and decline of motor

© 2025 The Authors. Published by Elsevier Inc.
This is an open access article under the CC BY-NC license (http://creativecommons.org/licenses/by-nc/4.0/).

skills, histopathology of storage material accumulations in the brain, and premature death.[11,12] In addition, ERT successfully reversed histopathology and improved motor skills in CLN2 mice.[13] However, CLN2 mice did not display the ocular disease phenotypes, indicating their unsuitability as a model for pharmacologic evaluation of potential retinal therapies.[11] Finally, in a CLN2 canine model, the disease is characterized by inner retinal degeneration,[14–17] whereas in patients with CLN2, the disease manifests as outer retinal degeneration.[18,19] Consequently, the lack of a relevant animal model urges the development of alternative retinal systems for disease modeling, drug development, and target validation.

Recent advances in stem cell technologies allowed the differentiation of human induced pluripotent stem cells (hiPSCs) into three-dimensional (3D) retinal organoids (ROs) and retinal pigmented epithelial (RPE) cells.[20,21] ROs are self-assembling, laminated 3D structures containing all major retinal cell types, such as rods, cones, Müller glia, amacrine, horizontal, bipolar, and ganglion cells.[20] Single-cell RNA sequencing (scRNA-seq) highlighted a strong similarity of the cell composition between ROs and fetal retinal tissue at equivalent developmental stages.[22] Deriving RPE cells and ROs from patient-derived hiPSCs made it possible to study disease phenotypes caused by specific mutations or genetic backgrounds, thus enabling to test and evaluate drug effects in a disease- and patient-specific manner.[23–25] Despite their relevance for disease modeling, toxicology studies, and drug development, RO use in nonclinical drug testing is still hampered by limitations compared to *in vivo* human retinal tissue, including the absence of physiological interaction between RPE cells and photoreceptors, lack of vasculature, and cultivation in static cell culture plates.

Organ-on-chip (OoC) technology has emerged in the last decade as a potentially powerful tool for pharmaceutical research and development by providing patient- and disease-specific microphysiological *in vitro* models and serving as an alternative to animal testing toward the 3Rs (reduction, replacement, and refinement).[26] To recreate organ-level functionality, OoCs integrate human tissues recapitulating *in vivo* structure and cellular interactions in a physiological microenvironment including vasculature-like microfluidic perfusion.[27] We previously developed a retina-on-chip (RoC) system that recapitulates key aspects of retinal biology by combining RPE cells and ROs in a tailored microfluidic platform, demonstrating increased outer segment formation in photoreceptor cells as well as outer segment phagocytosis by RPE cells.[28] The RoC has been successfully employed as a screening platform to test the transduction efficiency and cell tropism of different types of adeno-associated viral (AAV) vectors after a subretinal-like administration.[29]

In this study, we established and characterized an *in vitro* human disease model of the CLN2 retinal phenotype using patient hiPSC-derived ROs, RPE cells, and RoCs. By delivering the *TPP1* transgene, we employed an experimental gene therapy for CLN2 and showed a rescue of TPP1 expression as well as a substantial amelioration of the disease phenotype. Moreover, we used scRNA-seq technology to uncover gene expression differences between control and CLN2 organoids as well as to investigate the cell tropism of an AAV9-based TPP1 vector (AAV9.hCLN2) in CLN2 ROs. Finally, we used the RoC as a platform for nonclinical pharmacological investigation and validation of gene therapy.

RESULTS

CLN2 ROs display TPP1 deficiency and normal cell type composition

HiPSCs derived from fibroblasts from two patients with CLN2 (CLN2-1 and CLN2-2) and two commercially available healthy hiPSC control lines (CTRL1 and CTRL2) were differentiated into ROs. Both patients with CLN2 are compound heterozygotes with point mutations in both *TPP1* alleles. CLN2-1 harbors nonsense mutations in exon 4 (c.379C>T) and 6 (c.622C>T), both leading to premature termination of TPP1 protein translation. CLN2-2 carries a missense mutation in exon 4 (c.380G>A) and a transversion in intron 5 (IVS5-1G>C). c.622C>T and IVS5-1G>C are among the most common mutations identified in patients with CLN2.[30–32] To investigate the effect of two of these mutations on TPP1 translation, we overexpressed wild-type, c.379C>T, and c.380G>A *TPP1* variants in HEK293T cells (Figure S1A). Immunofluorescent evaluation confirmed the absence of TPP1 protein in cells expressing the mutant variants (Figure S1B). Inhibition of nonsense-mediated decay (NMD) using NDMI14 failed to restore expression, suggesting an NMD-independent degradation mechanism (Figure S1B).

CLN2 and control ROs were collected at different time points for morphological and molecular analysis (Figure 1A). At day 84 of differentiation, both displayed typical columnar neuroepithelium and substantial *RCVRN*/recoverin expression, marking photoreceptors (Figures S1C and S1E). Around day 200, CLN2 and control ROs exhibited a continuous layer of recoverin-positive photoreceptors aligned at the apical side (Figure 1E), as well as outer segment formation (Figure S1D).

To evaluate how TPP1 mutations impact RO composition and gene expression, we performed scRNA-seq at day 192 using the 10× Genomics platform. Major retinal cell types (rods, cones, Müller glia, bipolar, amacrine, and horizontal cells) were detected in both CLN2 and control ROs using known marker gene sets (Figures 1B, 1C, and S2A). Ganglion cells, typically absent after day 126 in ROs,[33] and RPE cells (manually excised from ROs) were not assessed. Cell type proportions were comparable across all samples (Figure 1C).

TPP1 transcripts were detected in all retinal cell types (Figures 1F and S2B). In control ROs, highest proportions of *TPP1*-expressing cells were found to be Müller glia (20% of cells expressing *TPP1*), cones (~17%), and horizontal cells (~16%) (Figure 1G). CLN2-2 showed similar expression to controls, while CLN2-1 had markedly lower TPP1 transcript levels across all retinal cell types.

In control ROs, TPP1 protein expression was nearly undetectable at day 84 but increased over time (Figure 1H). At day 200, TPP1 was observed in all layers of the control ROs with a prominent accumulation at the outer rim of the RO and the outer plexiform layer (Figure 1H), partially co-localizing with recoverin (Figure 1I). Furthermore, TPP1 displayed a punctate appearance and co-labeled with the lysosome marker lysosome-associated membrane protein 1 (LAMP1), thus confirming lysosomal sub-localization of TPP1 in control organoids (Figure 1J). In contrast to the

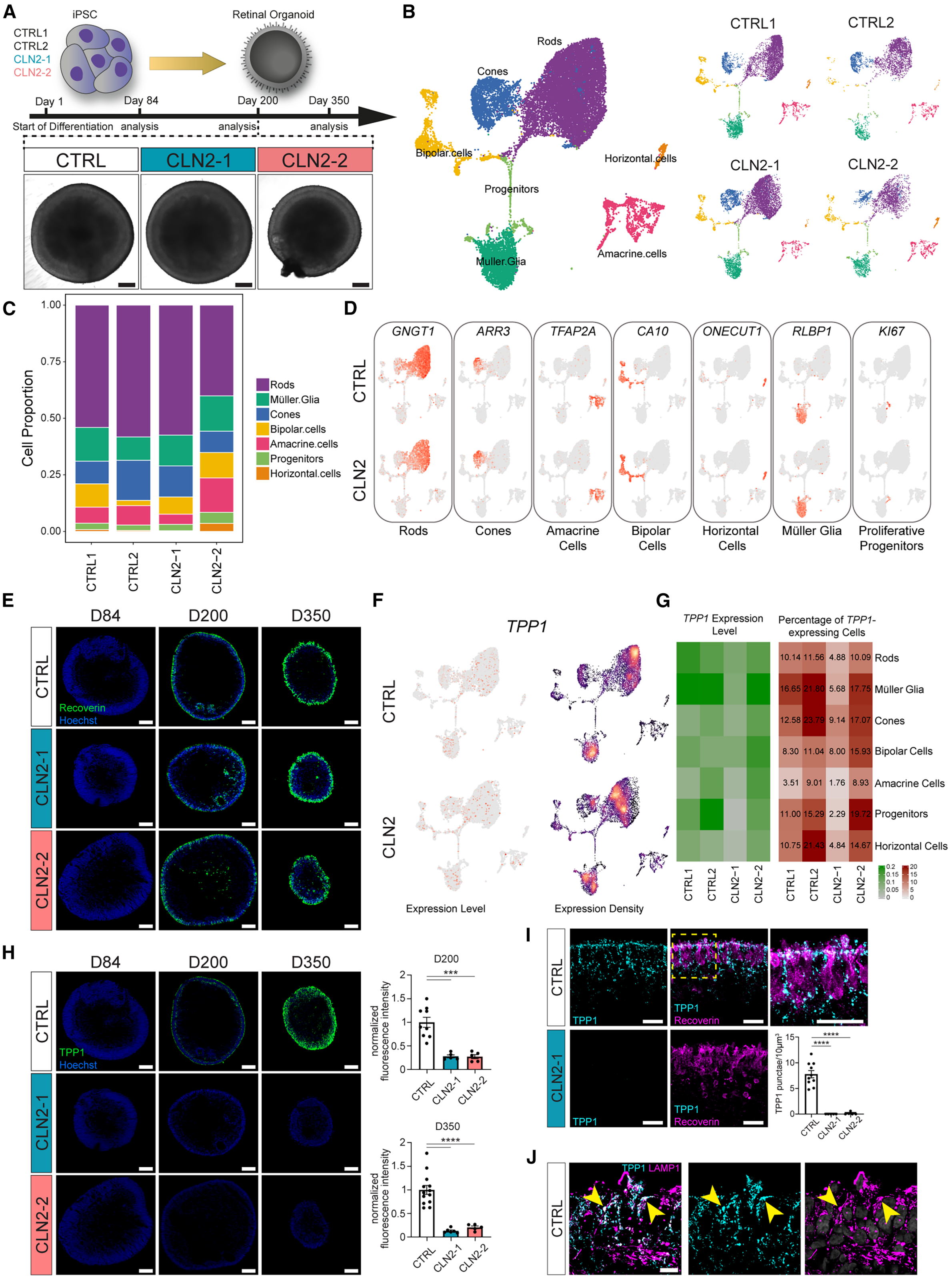

(legend on next page)

mRNA levels, TPP1 protein expression was substantially reduced at all analyzed time points: 72%–73% lower at day 200 and 87%–80% at day 350 (Figure 1H). Western blotting at day 350 confirmed the complete absence of TPP1 protein in both CLN2 lines (Figure S1F). High-magnification analysis showed that TPP1 punctae numbers were reduced by 99.96% and 98.2% in CLN2-1 and CLN2-2, respectively (Figure 1I). Lastly, CRISPR-Cas9-mediated heterozygous correction of the c.380G>A mutation in CLN2-2 restored TPP1 protein in isogenic ROs (CLN2-2ISO) to >50% of CTRL levels by day 123, while TPP1 remained absent in uncorrected CLN2-2 ROs (Figures S1G–S1I and S1K).

Characterization of CLN2 ROs reveals SCMAS and lipid droplet accumulations

The major histological manifestation of CLN2 pathology is lysosomal accumulation of autofluorescent intracellular lipofuscins, composed of proteins (among which the most abundant is SCMAS) and lipids.[34] To investigate the presence and composition of lysosomal storage bodies in CLN2 ROs, we first analyzed the expression of the lysosomal protein lysosome-associated membrane protein 2 (LAMP2) at day 200 (Figure S3C). Immunofluorescent analyses showed a comparable number of LAMP2^{+} lysosomes in CLN2-1 and control ROs, whereas CLN2-2 ROs displayed a slight, although not significant, increase of lysosome number that was mainly observed within the photoreceptor layer (Figure S3H). In addition, the mean volume of LAMP2^{+} lysosomes showed slightly higher levels, but was not significantly increased, in both CLN2 lines (Figure S3I). Lipid accumulations (labeled via LipidSpot) showed a significant 5.6-fold increase in lipid droplet accumulation in CLN2-1 but not in CLN2-2 ROs compared to controls (Figure 2A).

Next, we investigated the accumulation of SCMAS, the most abundant protein component of lipofuscins, in patients with CLN2. While absent in day 84 ROs, SCMAS^{+} accumulations substantially increased at later time points in CLN2 ROs. At day 200, a 5.2-fold and 3.3-fold increase was observed in CLN2-1 and CLN2-2 ROs, respectively, whereas 7.5-fold (CLN2-1) and 4.5-fold (CLN2-2) increases were documented at day 350 (Figure 2B) compared to control ROs. To identify the first developmental time point at which SCMAS accumulation is noticeable, SCMAS staining was performed at different stages between days 84 and 200. Our investigation reveals SCMAS accumulation as early as day 123 (Figure 5A). Of note, a weaker baseline of SCMAS expression was also detected in CTRL RO (Figure S3J). Finally, SCMAS accumulation was evaluated in isogenic controls of CLN2 lines originated by CRISPR-Cas9-mediated correction of *TPP1* mutation. At day 123, SCMAS accumulation was significantly higher in CLN2-2 ROs compared to CTRLs and CLN2-2ISO ROs (Figures S1J and S1L), thus indicating that SCMAS accumulation phenotype correlates with *TPP1* mutation and not with a cell line-specific genetic background.

At high magnification, SCMAS accumulations displayed a discrete punctate appearance. Particle quantification confirmed that SCMAS accumulations were 45.6 times more abundant in CLN2-1 and 37.2 times more abundant in CLN2-2 ROs compared to the control (Figure 2E). Interestingly, SCMAS accumulations were larger in CLN2-1 than in CLN2-2 ROs (Figure 2E). Finally, SCMAS punctae co-localized with yellow-emitting autofluorescent accumulations that were more abundant in day 350 CLN2 organoids compared to controls (Figure 2C). Of note, although SCMAS accumulations were primarily detected in CLN2 ROs, SCMAS protein expression was also seen in both CTRLs and CLN2 samples.

To delineate the exact retinal cell types containing SCMAS punctae, we performed co-staining of SCMAS with the photoreceptor marker recoverin or the Müller glia marker cellular retinaldehyde-binding protein (CRALBP), which represent the most disease-relevant cell types of the retina (Figure 2D). Confocal microscopy analysis showed a high degree of colocalization of SCMAS with recoverin (around 50% in all lines) (Figure 2F) and CRALBP (around 40% in controls and CLN2-1 and around 30% in CLN2-2) (Figure 2E).

To uncover the subcellular localization of SCMAS, we performed co-staining with the mitochondrial markers HSP60 and TOMM20 and the lysosomal marker LAMP2. SCMAS punctae displayed poor co-localization with HSP60 and TOMM20 (Figures S3A, S3E, S3B, and S3F), whereas co-labeling with LAMP2 confirmed that SCMAS mainly accumulates in lysosomes in both control and CLN2 ROs (Figures S3C and S3G). Some SCMAS punctae were HSP60/TOMM20 and LAMP2 negative in both control and patient organoids, suggesting the presence of extra-lysosomal accumulation, as previously reported in a CLN2 mouse model.[35] Finally, SCMAS accumulations partially co-localized with lipids (LipidSpot) in day 200 organoid (Figure S3D).

Figure 1. Characterization of CLN2 ROs

(A) Schematic of the hiPSC lines, RO differentiation protocol, and analysis time points (days 84, 200, and 350) and bright-field image of ROs at day 200.

(B and C) Uniform manifold approximation and projection (UMAP) of a single-cell RNA-seq dataset from ROs at day 192 ($n = 2$ CTRLs, 2 CLN2s) and (C) cell type composition.

(D) UMAP of cell type-specific markers (*GNGT1*: rods; *ARR3*: cones; *TFAP2A*: amacrine cells; *CA10*: bipolar cells; *ONECUT1*: horizontal cells; *RLBP1*: Müller glia; *KI67*: proliferative progenitors).

(E) Recoverin (photoreceptors) immunostaining in CTRL1 and CLN2 ROs.

(F) UMAP of *TPP1* gene expression as expression levels (left) and expression density (right).

(G) Heatmap of *TPP1* expression ($counts_{TPP1}/counts_{cell}$*10,000) and percentage of *TPP1*-expressing cells.

(H) TPP1 immunostaining and quantification in CTRL (image: CTRL1) and CLN2 ROs at days 84, 200, and 350. Values were normalized on TPP1 expression in CTRLs. $n = 5$ ROs, one differentiation.

(I) Single confocal plane of TPP1 and recoverin in ROs at day 200. Yellow-dashed square: magnified area in the third column. $n = 5$ ROs from one differentiation. Graphs shows number of TPP1 punctae per 10 μm^3.

(J) Single confocal plane showing colocalization of TPP1 with LAMP1 at day 350. Arrowhead: examples of colocalizing.

Values: mean ± SEM. Scale bars: (A) 200 μm, (E, H) 100 μm, (I) 25 μm. Hoechst: (E, H) blue, (J) gray.

$^{*}p < 0.05$, $^{**}p < 0.01$, $^{***}p < 0.001$, $^{****}p < 0.0001$.

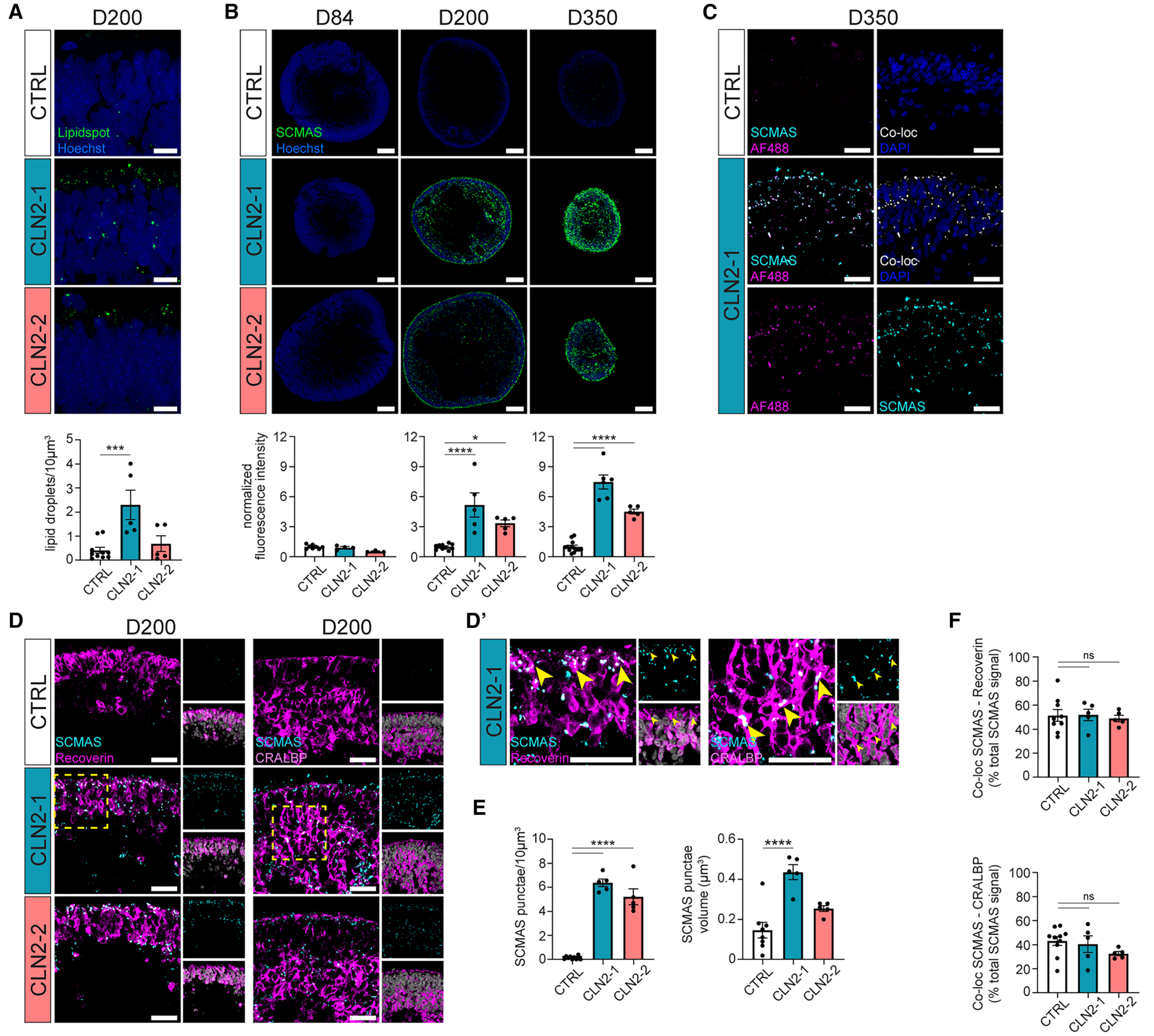

Figure 2. Autofluorescence, SCMAS, and lipid accumulation in CLN2 ROs

(A) LipidSpot and quantification of lipid droplets per 10 μm^3 at day 200 CTRL (image: CTRL1) and CLN2 ROs. Hoechst: blue. $n = 5$ ROs from one differentiation.
(B) SCMAS immunostaining and quantification in CTRL (image: CTRL1) and CLN2 ROs at days 84, 200, and 350. $n = 5$ ROs from one differentiation.
(C) Single confocal plane showing co-localization of SCMAS and green autofluorescence in day 350 CTRL (image: CTRL1) and CLN2 (image: CLN2-1) ROs. SCMAS and autofluorescent co-localization: white.
(D) Single confocal plane showing co-localization of SCMAS with recoverin and CRALBP in CTRL (image: CTRL1) and CLN2 ROs at day 200. Yellow dashed square: magnified area in (D′). (D′) Yellow arrowheads: examples of colocalizing signal.
(E) Quantification of SCMAS punctae per 10 μm^3 and SCMAS punctae volume in CTRL (CTRL1, CTRL2) and CLN2 ROs at day 200. $n = 5$ ROs from one differentiation.
(F) Co-localization percentage of SCMAS with recoverin and CRALBP in CTRL (CTRL1, CTRL2) and CLN2 ROs at day 200. $n = 5$ ROs, one differentiation.
Values are mean ± SEM. (A, B) Values normalized to CTRL ROs. Scale bars: (A) 10 μm, (B) 100 μm, (C, D) 25 μm. Hoechst: (A, C) blue, (D, D′) gray. *$p < 0.05$, **$p < 0.01$, ***$p < 0.001$, ****$p < 0.0001$.

Electron microscopy reveals curvilinear profiles in CLN2 organoids

At the ultrastructural level, most storage material observed in CLN2 disease has been described as "curvilinear profiles" (CPs), uniformly curved, short, thin, lamellar stacks of alternating dark and light lines.[6] CPs have been observed in retinal cells (photoreceptor, Müller glia, RPE, and ganglion cells)[36] and ocular tissue from patients with CLN2,[37] CLN2 hiPSC-derived neurons,[38] CLN2 canine retina models,[14] and brains of CLN2 mouse.[11,35,39] Fingerprint deposits (fingerprint profiles [FPs]), which consist of

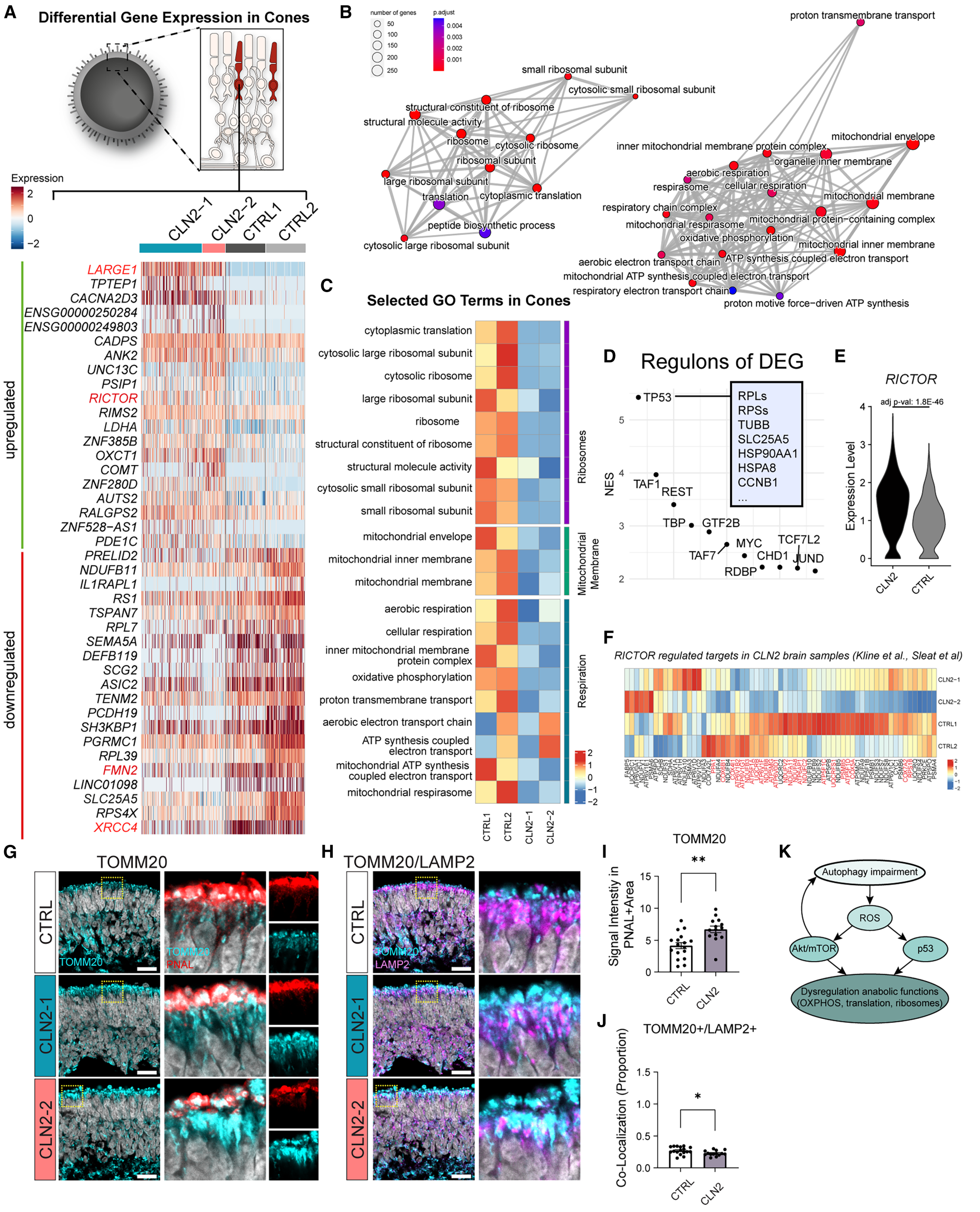

(legend on next page)

CellPress
OPEN ACCESS

membrane-bound, electron-dense bodies composed of paired parallel dark lines,[6] have been described in patients with CLN2,[40] although they are more common in CLN3.[41]

To investigate storage material deposition in CLN2 ROs, we performed electron microscopy evaluation of day 300 ROs, focusing on photoreceptor inner segments. CPs and FPs were absent in CTLRs, whereas 7.9% and 2.7% of the inner segment area in CLN2-1 and CLN2 was covered by CPs, respectively (Figures S3K and S3M). Interestingly, CLN2-2 segments also displayed FPs (Figures S3L) that were not found in the other CLN2 line.

Cones in CLN2 ROs display dysregulation of translation and mitochondrial respiratory gene expression

Patients with CLN2 develop a symmetrical cone-rod dystrophy, with early structural optical coherence tomography (OCT) signs of maculopathy and high proportion of cone loss.[18] To investigate whether early degenerative changes can be observed in cones, we performed differential gene expression (DGE) analysis on pseudobulked scRNA-seq data (Figure S4), bulk RNA sequencing (RNA-seq) data (Figure S4), as well as on the cone cluster in the scRNA-seq dataset (Figure 3). In cones, several genes associated with cell migration (LARGE1), mechanistic target of rapamycin (mTOR) signaling (RICTOR), microcephaly (XRCC4), and intellectual developmental disorder (FMN2) were strongly dysregulated (Figure 3B). Bulk RNA-seq confirmed LARGE1 as one of the most upregulated genes in CLN2 organoids (Figures S4A and S4B), supported by immunostaining that revealed the presence of LARGE1 accumulations in the inner layers (Figure S4C). Gene Ontology (GO) analysis of the bulk RNA-seq data found endosomes, microtubule, and lysosomal-associated proteins to be most highly dysregulated (Figures S4E and S4F). A comparison of GO terms from the bulk RNA-seq with pseudobulked scRNA-seq shows a strong overlap (Figures S4F and S4G).

Gene set enrichment analysis of the cone cluster identified three major gene cluster families: mitochondrial membrane proteins, respiration/oxidative phosphorylation (OXPHOS), and ribosomal/translational proteins (ribosomal protein, large subunits [RPLs] and ribosomal protein, small subunit [RPSs]) (Figure 3B). Subsequent UCell scoring identified 19 of 21 tested gene sets related to those 3 families to be decreased (Figure 3C).

To identify common master transcription factors (TFs) in the cone scRNA-seq dataset, gene regulation analysis (iRegulon) was employed (Figure 3D). The most enriched common master TF found was *TP53*, which is associated with cell division and protein biosynthesis/translation.[42,43] Indeed, among the dysregulated TP53-regulated genes in cones, we identified several proteins involved in translation (RPSs and RPLs genes) (Figure 3D).

Dysregulation of *RICTOR*, a regulator and key component of the mTOR2 complex, was previously reported as a potential driver of changes observed in brains of patients with CLN2, such as altered OXPHOS and mitochondrial dysfunctions.[44] In accordance, *RICTOR* was also found highly upregulated in cones of CLN2 ROs (Figure 3E). In a meta-analysis, Kline and colleagues found that 66 genes previously associated with RICTOR were differentially expressed in CLN2 brain samples.[44] Among these 66 genes, 24 were also identified to be significantly dysregulated in the CLN2 RO cone cluster (Figure 3F), indicating a potential common mechanism involving RICTOR and mTOR.

To examine the involvement of cone mitochondria in the pathomechanism, we assessed the mitochondrial marker TOMM20 in photoreceptor cones segments (Figures 3G and 3I) using co-labeling with the cone segment marker PNA lectin, which revealed a significant increase in CLN2 ROs (Figures 3G and 3I). Decrease of TOMM20/LAMP2 co-localization (in whole ROs) in CLN2 ROs (Figures 3H and 3J) indicates that the increase of cone mitochondrial TOMM20 signal in CLN2 ROs could be caused by decreased mitophagy. In summary, we hypothesize a mechanism that potentially involves AKT/mTOR dysregulation caused by cell stress or autophagy impairment, which impacts translation and mitochondrial function in CLN2 cones (Figure 3K).

AAV9.hCLN2 treatment of CLN2 ROs restores TPP1 expression

In order to restore TPP1 protein level and function in the ROs, an AAV9 vector was employed to deliver a *TPP1* transgene under a modified CB7 promoter, a hybrid between a cytomegalovirus immediate-early enhancer and the chicken beta-actin promoter.[45,46]

Figure 3. scRNA-seq highlights dysregulation of protein translation and mitochondrial function in CLN2 RO cones

(A) Differential gene expression (DGE) analysis performed on the cone cluster of the scRNA-seq dataset (n = 2 CTRL and 2 CLN2 RO samples). Heatmap shows top 25 up- and downregulated genes sorted by a Bonferroni-corrected p value in individual cells of each line. Notable genes are highlighted in red.

(B) Network plot (CNET) of a gene set enrichment analysis (GSEA) comparing Gene Ontology (GO) terms (biological processes, cellular components, and metabolic function) of cones. Node color: adjusted p value of enrichment. Node size: number of genes in the core enrichment set.

(C) UCell score of selected GO terms of three clusters (ribosomes, mitochondrial membrane, and respiration) enriched in the GSEA analysis. Color: average-scaled U-score.

(D) iRegulon analysis of cone DGE (CLN2s vs. CTRLs). y axis: normalized enrichment score (NES) of each depicted transcription factor in DGE cone dataset. *TP53*-selected downstream targets are depicted in the light blue box.

(E) *RICTOR* (regulator of the mTOR complex 2) expression in cones. Adjusted p value: Wilcoxon test and Bonferroni correction.

(F) Gene expression heatmap of downstream targets of *RICTOR* (enriched in a CLN2 brain dataset from Sleat et al., meta-analysis performed by Kline et al.). Red-labeled genes were found significantly different in cones of RO in our dataset.

(G and H) Single confocal plane showing TOMM20 with (G) PNA lectin (PNAL) and (H) LAMP2 in ROs at day 158. Scale bars, 20 μm.

(I and J) Quantification of TOMM20 signal in the PNAL+ area (I) and TOMM20/LAMP2 co-localization (J). Values are mean ± SEM. n = 14–17 ROs from two differentiations, respectively.

(K) Putative dysregulation mechanisms in cones of CLN2 ROs. Hoechst: gray.

*$p < 0.05$, **$p < 0.01$, ***$p < 0.001$, ****$p < 0.0001$.

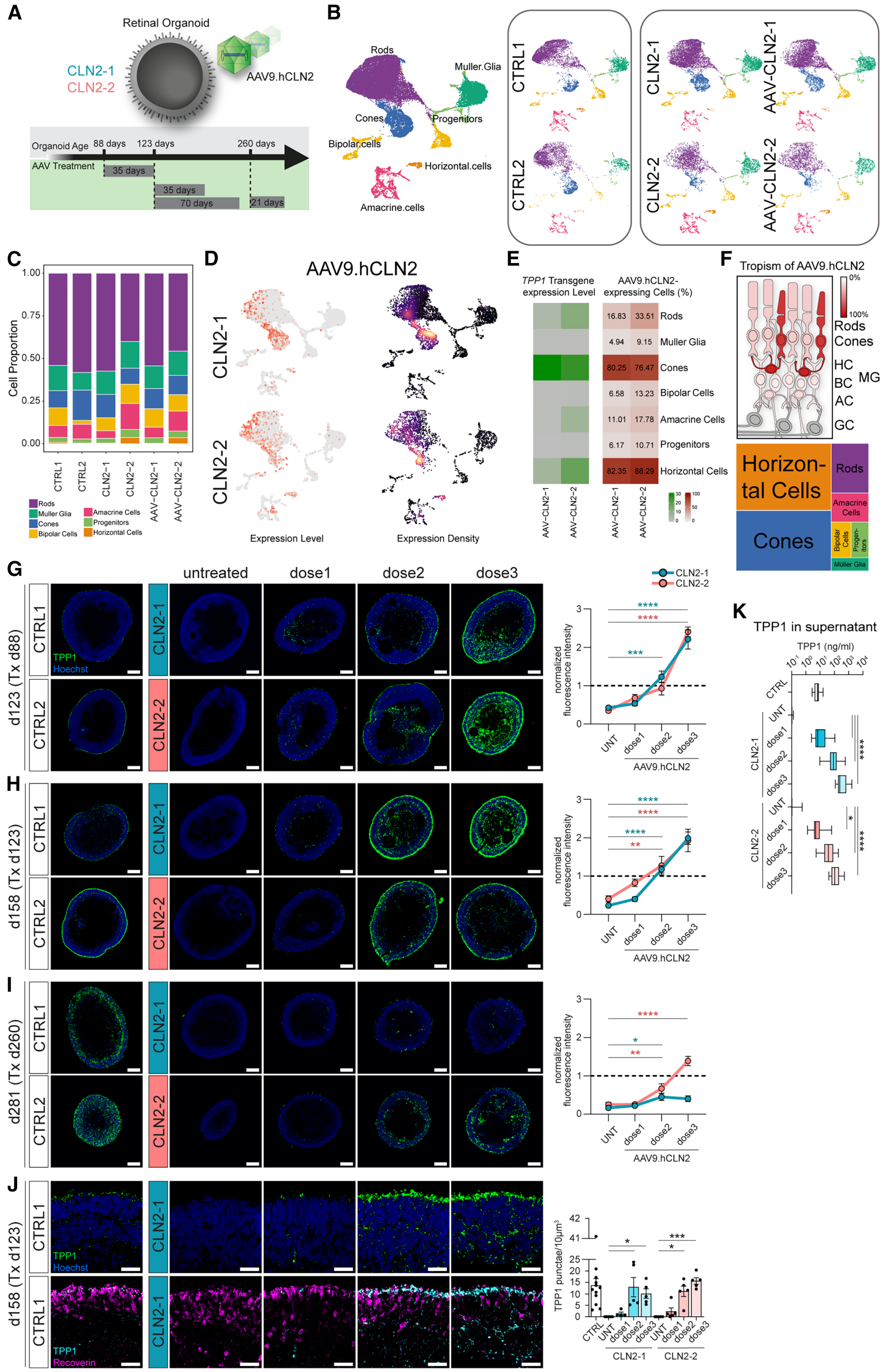

(legend on next page)

To assess viral transduction efficiency and tropism, ROs were treated with AAV9.hCLN2 (1.67 $\times$ 10^{11} gc/RO [genome copies per RO]) at day 123 and subjected to scRNA-seq analysis after 10 weeks (Figure 4A). Treated CLN2 ROs (AAV-CLN2) contained retinal cell types in similar proportion to their untreated counterpart and to controls (Figures 4B and 4C). *TPP1* transgene (AAV9.hCLN2) was expressed in all retinal cell clusters, with highest expression levels in horizontal cells (84% of cells expressed *TPP1* transgene), cones (78%), and rods (27%) (Figures 4D and 4E).

TPP1 protein levels were assessed in AAV-treated CLN2 ROs treated at days 88, 123, or 260 with 3 different dosages of AAV9.hCLN2 (dose 1: 5 $\times$ 10^9, dose 2: 5 $\times$ 10^{10}, and dose 3: 1.67 $\times$ 10^{11} gc/RO). Immunofluorescence analysis and electrochemiluminescence (ECL) assays were performed after 3 to 5 weeks (Figure 4A). In CLN2 ROs, treatment with AAV9.hCLN2 was sufficient to rescue TPP1 protein expression in a dose-dependent manner at all 3 analyzed time points, with higher TPP1 levels in the two youngest ROs (days 88 and 123) (Figures 4G and 4H). When treated with dose 2, CLN2 ROs showed a significant recovery of TPP1 protein expression that reached control levels in young and intermediate ROs (Figures 4G and 4H) and 55% of control levels in old ROs (Figure 4I). Furthermore, when CLN2 ROs were transduced with dose 3, TPP1 expression was up to 2.3 times higher than the control levels (Figures 4G–4I). As expected, CLN2 ROs treated with an AAV9.Null vector displayed TPP1 levels comparable to the untreated CLN2 ROs (Figure S5A).

Quantification at a high magnification showed that TPP1 protein displayed a punctate appearance in AAV-treated CLN2 ROs similar to the controls (Figure 4J). Moreover, CLN2 ROs treated with dose 2 and dose 3 featured TPP1 punctae number (Figure 4J) and intensity (Figure S5C, right) comparable to control ROs and had higher TPP1 punctae volume (Figure S5C, left). Consistent with the immunofluorescence data, we observed a dose-dependent increase of TPP1 protein levels in the supernatant of treated CLN2 organoids (Figure 4K), indicating TPP1 secretion both from healthy control and from AAV-treated CLN2 ROs.

AAV9.hCLN2 treatment of CLN2 RO obliterates SCMAS accumulation

AAV9.hCLN2 transduction at day 88 (before the onset of detectable SCMAS deposits) resulted in a dose-dependent prevention of SCMAS accumulation (Figure 5A). Although AAV9.hCLN2 dose 1 only caused a slight increase in TPP1 expression (Figure 4G), it was sufficient to drastically decrease SCMAS accumulation (Figure 5A). After the beginning of SCMAS deposition (day 123), dose 3 was required to decrease SCMAS level to that of control RO (Figure 5B). In contrast, in CLN2 ROs treated at day 260, SCMAS level decreased but did not reach control levels (Figure 5C), possibly due to the higher initial storage material burden in mature ROs. Of note, SCMAS level was unchanged in CLN2 ROs treated with an AAV9.Null vector (Figure S5B).

High-magnification image analysis of AAV9.hCLN2-treated ROs at day 158 showed a dose-dependent decrease of SCMAS punctae number with both CLN2 lines (Figure 5D), while punctae volume and signal intensity were largely unaffected (Figure S5D).

CLN2 adherent RPE cultures display TPP1 reduction and SCMAS accumulation

Ophthalmic evaluation of patients with CLN2 and electron microscopy inspection of postmortem retinas revealed abnormalities and scattered loss of RPE cells.[19,36] Moreover, scRNA-seq of unaffected human adult postmortem retina showed that RPE cells feature high *TPP1* expression.[33]

To understand if CLN2 hiPSC-derived RPE cells reflect the disease phenotype, we generated RPE dissociated from RPE organoids between days 120 and 150 (Figure S6A).[47] After 4 weeks of culture, control and CLN2 RPE cells formed a pigmented monolayer with a characteristic honeycomb structure and expression of the tight junction marker zonula occludens-1 (Figure S6B). Immunofluorescence analysis revealed a high and relatively uniform expression of TPP1 in the control lines and a marked expression deficiency in the patient lines (82% and 64% lower in CLN2-1 and CLN2-2, respectively, compared to the controls) (Figure 6A). At that time point, no SCMAS accumulation was observed in CLN2 RPE (Figure 6A). Only after a prolonged culture of 13 weeks, a slight increase of SCMAS accumulation was documented (Figure 6D, CLN2-1 and CLN2-2 untreated panels).

To evaluate the transduction capability of AAV9.hCLN2, 2D-cultured RPE cells were treated with two amounts of AAV9.hCLN2 vector comparable to those used on ROs (dose 1: 1 $\times$ 10^5 and dose 2: 1 $\times$ 10^6 gc/cell) (Figure 6B). Nine weeks post treatment, immunofluorescence analysis showed a dose-

Figure 4. AAV9.hCLN2 delivery to CLN2 ROs restores TPP1 expression

(A) Schematic of AAV9.hCLN2 treatment of ROs.

(B and C) UMAP of a single-cell RNA-seq dataset derived from ROs at day 192 (n = 2 CTRLs, 2 CLN2 patient lines, and 2 AAV9.hCLN2-treated CLN2 patient lines) indicating individual cell types and (C) cell type composition.

(D) UMAP of TPP1 transgene expression in AAV9.hCLN2-treated ROs as expression levels and expression density.

(E) Heatmaps of *TPP1* transgene expression levels ($counts_{TPP1}/counts_{cell}$*10,000) and the percentage of *TPP1*-expressing cells (in %).

(F) Transduction efficiency of RO cell types. Top: cell types colored in shades of red proportionally to their TPP1 transgene expression. Ganglion cells (GCs, gray) were not found in day 192 ROs. Bottom: proportional area chart. HCs, horizontal cells; MGs, Müller glia; BCs, bipolar cells; ACs, amacrine cells.

(G–I) TPP1 immunostaining and quantification of ROs treated with AAV9.hCLN2 at days 88, 123, and 260. AAV9.hCLN2 dose 1: 5 $\times$ 10^9, dose 2: 5 $\times$ 10^{10}, and dose 3: 1.67 $\times$ 10^{11} gc/RO. Values were normalized to CTRL ROs (dashed line). Analyzed ROs: CLN2-1 n = 8–11; CLN2-2 n = 3–8; CTRL1 n = 9–14; CTRL2 n = 8–9.

(J) Single confocal plane and quantification of TPP1 in day 123 + 35 ROs treated with AAV9.hCLN2. n = 5 ROs, 2 experiments.

(K) TPP1 protein concentration in supernatants in day 123 + 35 ROs treated with AAV9.hCLN2, evaluated by electrochemiluminescence (ECL) immunoassay. Analyzed ROs: CLN2-1 n = 21–22, 3 experiments; CLN2-2 n = 16–18, 5 experiments; CTRL1 n = 32 from 5 experiments; CTRL2 n = 25, 3 experiments.

Values are mean ± SEM. Scale bars: (G–I) 100 μm, (J) 25 μm. Hoechst: blue. Tx: treatment. *$p < 0.05$, **$p < 0.01$, ***$p < 0.001$, ****$p < 0.0001$.

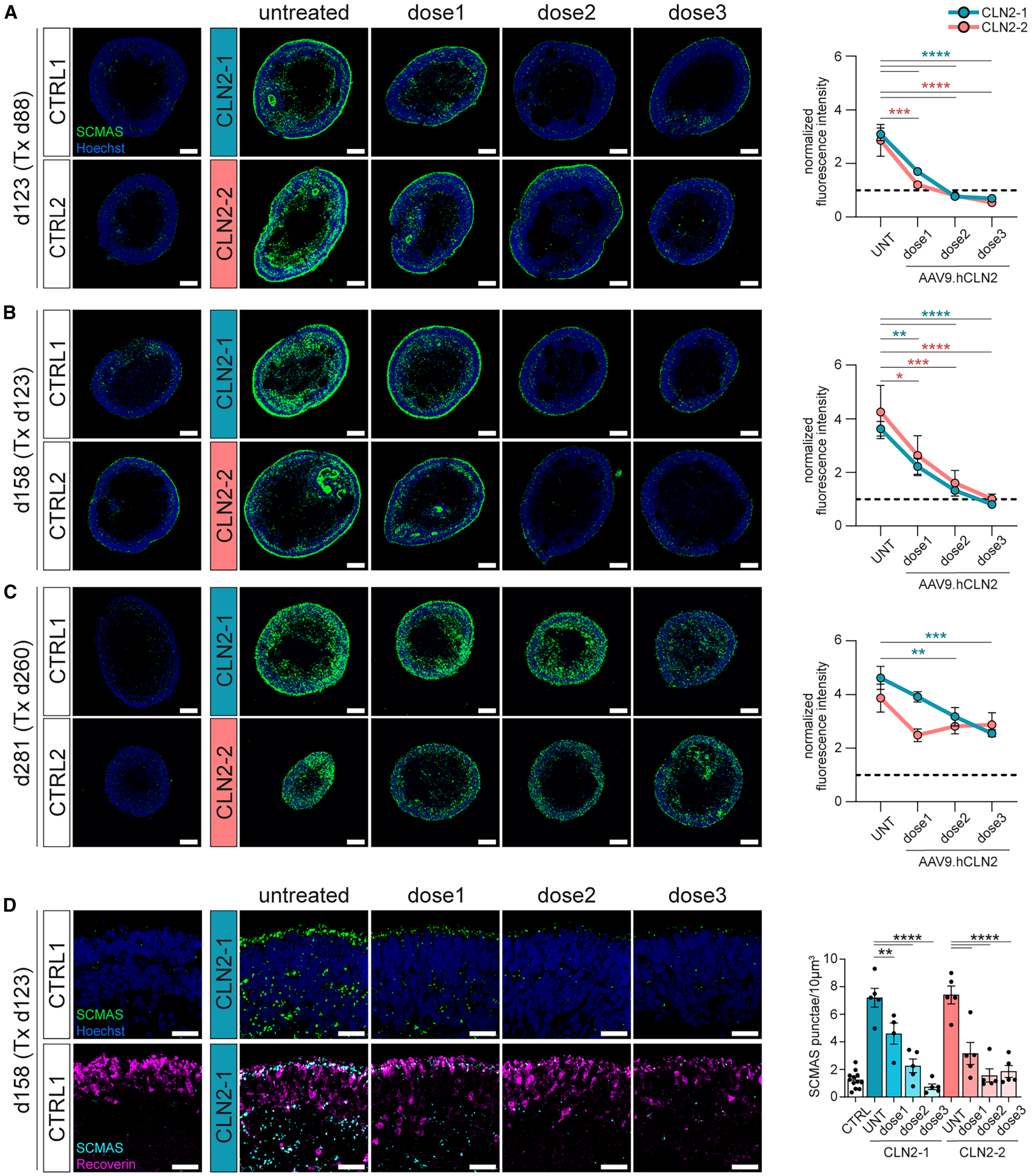

Figure 5. AAV9.hCLN2 treatment can decrease and prevent SCMAS accumulation in CLN2 ROs

(A–C) SCMAS immunostaining and quantification of ROs treated with AAV9.hCLN2 at days 88, 123, and 260. AAV9.hCLN2 dose 1: 5×10^9, dose 2: 5×10^{10}, and dose 3: 1.67×10^{11} gc/RO. Values were normalized on SCMAS expression in CTRL ROs = dashed line. Number of analyzed RO: see Figures 4G–4I.

(D) Single confocal plane of SCMAS immunostaining and quantification in day 123 + 35 ROs treated with AAV9.hCLN2. $N = 5$ ROs, two experiments. Values are mean ± SEM. Scale bars: (A–C) 100 µm, (D) 25 µm. Hoechst: blue. Tx: treatment.

CellPress
OPEN ACCESS

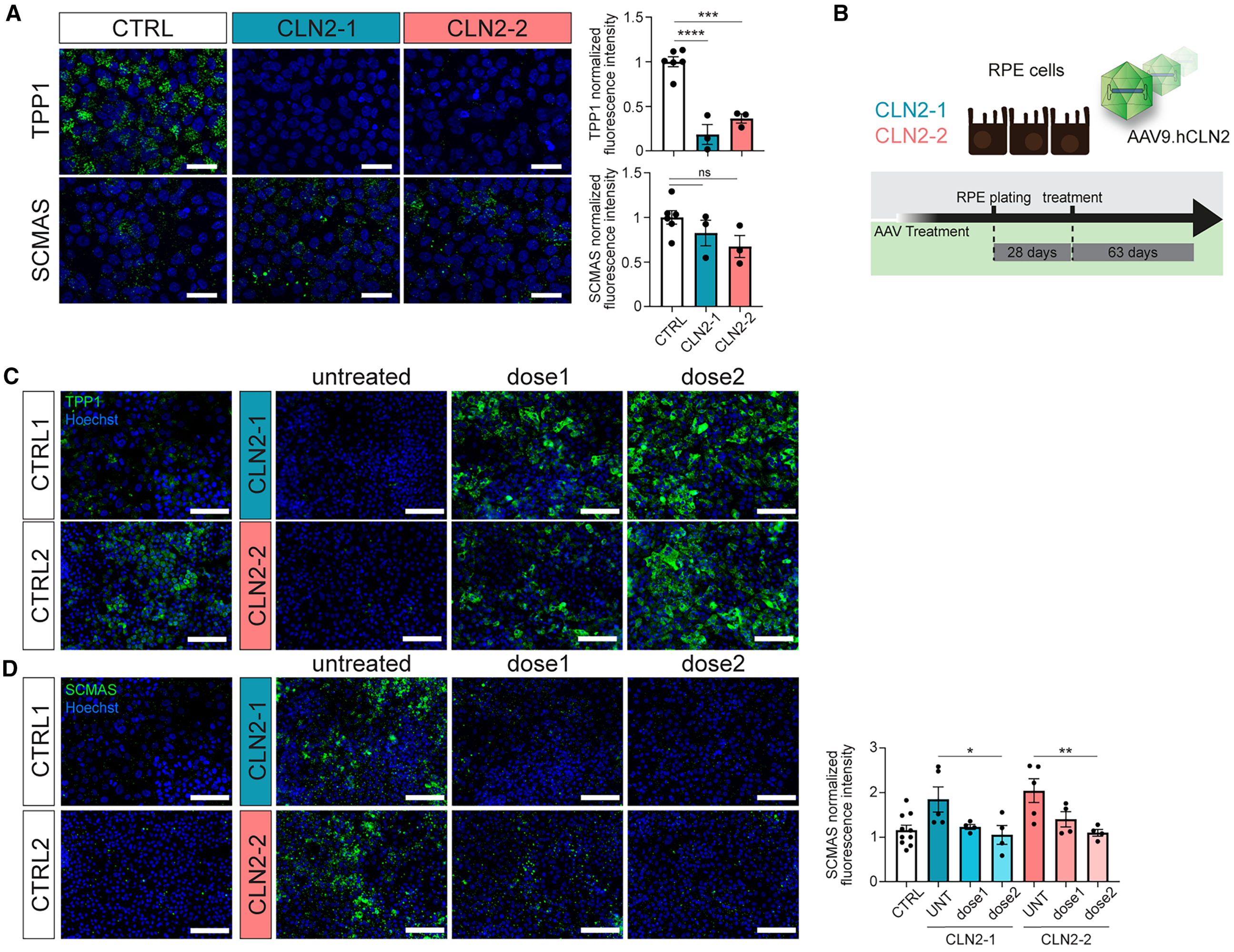

Figure 6. Characterization and AAV9.hCLN2 treatment of CLN2 RPE cells

(A) TPP1 and SCMAS immunostaining and quantification of hiPSC-RPE cultured for 4 weeks. $n = 3$, one differentiation.

(B) Schematics of AAV9.hCLN2 treatment of the hiPSC-RPE.

(C and D) TPP1 and SCMAS immunostaining and SCMAS quantification of hiPSC-RPE 63 days after treatment with AAV9.hCLN2. AAV9.hCLN2 dose 1: 10^5 gc/cell and dose 2: 10^6 gc/cell. $n = 4–5$, one differentiation.

Values are mean ± SEM. Scale bars: (A) 25 μm, (C, D) 100 μm. Hoechst: blue. Tx: treatment. $^*p < 0.05$, $^{**}p < 0.01$, $^{***}p < 0.001$, $^{****}p < 0.0001$.

dependent TPP1 restoration (Figure 6C) and SCMAS reduction (Figure 6D) in treated CLN2 RPE cells. Dose 2 restored SCMAS levels to control levels.

Evaluation of pharmacological properties of TPP1 gene therapy using the RoC platform

In order to model CLN2 in a complex tissue context, the RoC,[28] a tailored organ-on-chip platform allowing the proximity culture of RPE layers and ROs in a vasculature-like perfusion, was employed (Figure 7A). CLN2 RoCs were treated for 4 weeks with AAV9.hCLN2 at three different concentrations (dose 1–3), corresponding to the genome copies per cell used in the RPE and RO treatments. Similar to the treatment performed in ROs alone, we observed a dose-dependent increase of TPP1 and decrease of SCMAS accumulation in AAV9.hCLN2-treated RoC (Figures 7B and 7C). Immunohistochemical analysis showed that TPP1 expression in CLN2 ROs treated in the RoC was more than two times higher than in controls already at the lowest AAV9.hCLN2 dose (dose 1) (red line, Figure 7E), whereas ROs treated in culture (or "off-chip") dishes reached control levels only with dose 2 and 3 (gray line, Figure 7E). Consistent with these data, ECL assays showed that TPP1 levels were at least 100 times higher than control levels in dose 1-treated CLN2 RoC supernatant (red line, Figure 7G), whereas they were only around 2 times higher in the off-chip RO culture subjected to the same AAV dose (gray line, Figure 7G). A dose-dependent increase of TPP1 was also observed in the RPE layer of treated CLN2 RoCs (Figure 7D). All three doses efficiently suppressed SCMAS accumulation in the RoC treatment (Figure 7F). Of note, in the treated RoCs, SCMAS levels reached control levels already at dose 1 (red line, Figure 7F), whereas control SCMAS levels were reached only at dose 2 and 3 in off-chip ROs (gray line,

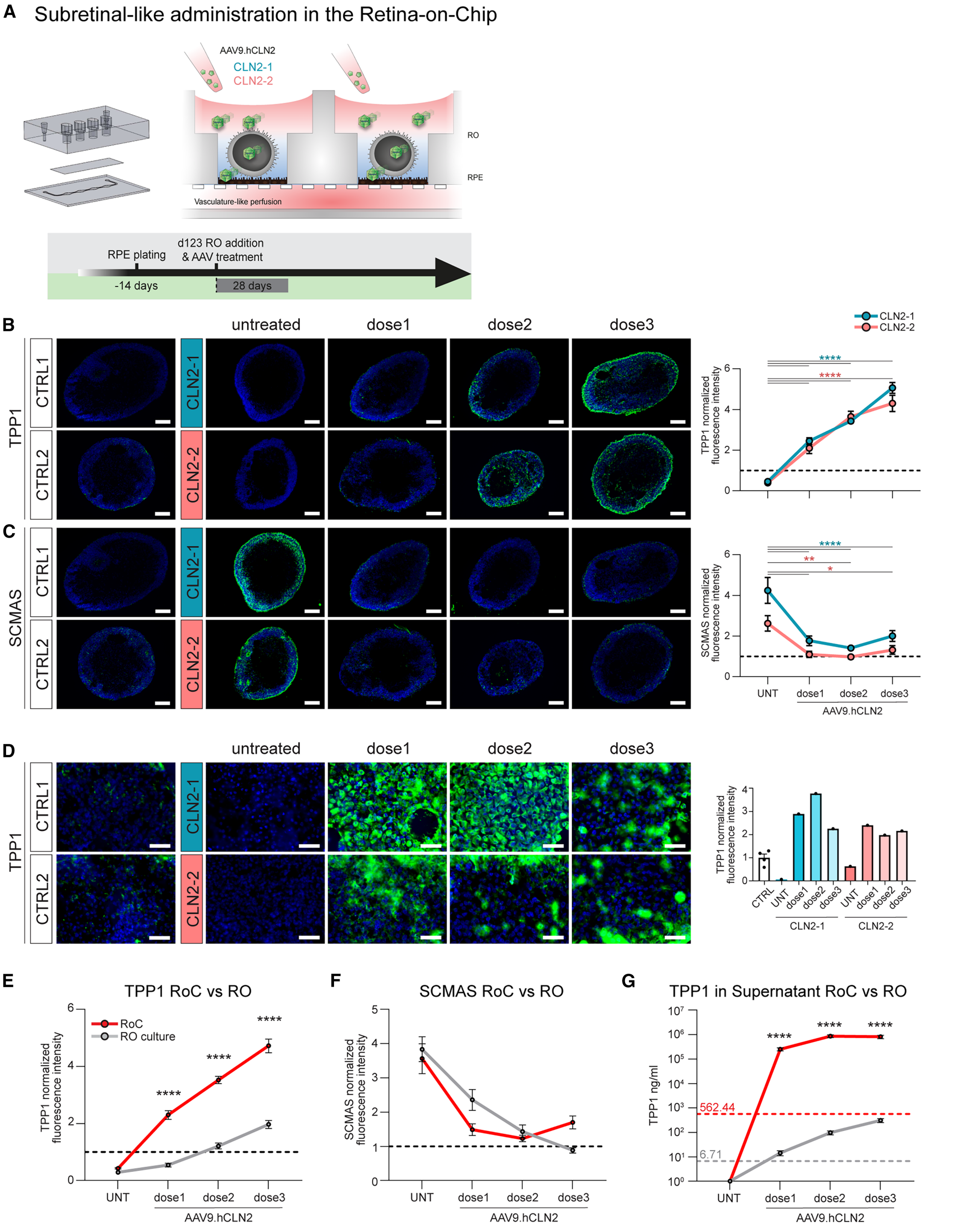

(legend on next page)

Figure 7F). To further investigate this difference, we compared the RoC to ROs that were cultured in the same volume and under the exact same conditions (without RPE, Figure S7). We found that TPP1 expression was significantly higher in RoC treated with equivalent doses than in a 384-well plate (Figures S7A and S7B). We therefore speculated for a paracrine effect of TPP1 expressed by the RPE cells in the RoC. However, when only the RPE cells were transduced with AAV9-hCLN2 in a RoC prior to the RO loading, no TPP1 signal could be found in the RO (Figure S7C).

DISCUSSION

SCMAS accumulation reflects *in vivo* pathology of CLN2 disease

Despite their utility in developmental biology and biomedical research, animal models do not accurately recapitulate human physiology and therefore are often not sufficient to understand disease progression and the response to a specific drug or therapeutic product. With respect to CLN2 disease, available mouse models do not display the ocular disease phenotype.[11] CLN2 canine models display inner retinal degeneration,[14–17] which differs from patients with CLN2, where the disease is characterized by outer retinal degeneration.[18,19]

In patients, progressive retina degeneration and visual loss are among the major hallmarks of CLN2 disease.[41,48] The gradual retinal deterioration appears to start in the outer retinal layers (RPE cells and photoreceptors) and advance from the macula toward the peripheral retina.[19] Retina degeneration correlates with the presence of autofluorescent SCMAS storage granules throughout the ocular tissue, most notably in retinal ganglion cells and the inner and outer nuclear layer cells.[49] Both CLN2 patient-derived hiPSCs employed in this study differentiated into laminated ROs containing all major retinal cell types, including rod and cone photoreceptors as demonstrated by scRNA-seq analysis and imaging. Importantly, even in advanced stages (day 350), CLN2 ROs did not show signs of drastic photoreceptor loss or degeneration. Nevertheless, a significant accumulation of SCMAS protein was observed starting from day 123 and increasing through day 350 in CLN2 ROs, thus recapitulating a major pathohistological hallmark of CLN2 disease. Especially in earlier stages, SCMAS accumulations were predominantly located at the outer rim of the outer nuclear layer where the photoreceptors of the RO reside. Co-localization analysis with recoverin confirmed that around 50% of SCMAS accumulations were located in photoreceptors. Interestingly, in CLN2 patient postmortem retinal biopsies, SCMAS storage was also observed in the outer nuclear layer[49]; however, in these studies, cell identification was uniquely based on morphology rather than cell type-specific markers. A low number of SCMAS punctae were also detected in control organoids starting from day 123. While, to the best of our knowledge, no clinical data from healthy retinal tissue are available, studies showed that a certain degree of SCMAS deposits can be observed in the brain of otherwise healthy individuals (60–91 years old).[49,50] Finally, CPs, the ultrastructural appearance of storage material observed in CLN2 disease, have been identified by electron microscopy in the photoreceptor segments of both CLN2 lines but not in the CTRLs.

Little is known about the onset of SCMAS accumulation in CLN2 retina pathophysiology. Fundus photographs and OCT imaging showed that rapid acceleration of outer retinal degeneration in patients with CLN2 between 4 and 6 years of age culminates in complete blindness around 8–10 years of age.[8,51] The very early onset of SCMAS accumulation detected in CLN2 ROs suggests that, in patients, the storage material might begin to deposit well before any vision symptom is noticed. In other degenerative eye disease models (such as retinitis pigmentosa), disease phenotypes were observed to appear at an earlier "age" in patient hiPSC-derived ROs than in patients.[23,25]

In patients with CLN2, SCMAS protein mainly accumulates in lysosomes together with other proteins and lipids.[34,52,53] Lipid and lysosome accumulation have also been reported in patient hiPSC-derived neural stem cells[54] and neural progenitor cells,[38] in the retina, forebrain, and hindbrain of CLN2 zebrafish embryo,[55] as well as in the brain of a CLN2 mouse model.[11] In day 200 control and CLN2 ROs, a large proportion of SCMAS punctae co-localized with the lysosomal marker LAMP2. Still, lysosome number was only increased in CLN2-2 line, whereas lysosome volume was slightly but not significantly increased in both CLN2 lines. Finally, lipid droplets were significantly accumulating only in the CLN2-1 line. Overall, our analyses indicate that lipid and lysosome accumulation are more subtle, whereas SCMAS accumulations represent the most pronounced *in vitro* CLN2 phenotype. Together with the lack of specific cell degeneration, this suggests that CLN2 ROs mainly reflect the early phase of disease progression.

Figure 7. Evaluation of AAV9.hCLN2 gene therapy in CLN2 RoC
(A) Schematics of AAV9.hCLN2 treatment of the RoC.
(B and C) TPP1 and SCMAS immunostaining and quantification of day 123 + 28 ROs treated with AAV9.hCLN2 in the RoC. AAV9.hCLN2 dose 1: 6.5×10^9, dose 2: 6.5×10^{10}, and dose 3: 2.17×10^{11} gc/well. TPP1 and SCMAS intensity in CTRL organoids are represented as dashed line. Analyzed ROs: CLN2-1 $n = 10–11$; CLN2-2 $n = 8$; CTRL1 $n = 16$; CTRL2 $n = 14$.
(D) TPP1 immunostaining and quantification of hiPSC-RPE cells in AAV9.hCLN2-treated RoCs. Number of analyzed RoC wells: CLN2-1, CLN2-2 $n = 1$; CTRLs $n = 4$.
(E and F) Quantification of TPP1 (E) and SCMAS (F) in ROs treated with AAV9.hCLN2 at day 123 + 35 in RO culture (gray line, treatment, doses, and *n*, see Figure 4) or at day 123 + 28 in RoC (red line, treatment, doses, and *n*, see B and C). Values were normalized on TPP1 or SCMAS expression in CTRL ROs or RoC = dashed line.
(G) TPP1 protein in supernatant of ROs treated with AAV9.hCLN2 in RO culture (gray line) or RoC (red line), evaluated by electrochemiluminescence (ECL) immunoassay. Gray and red dashed lines: average concentration of TPP1 in CTRL samples from RO culture and RoC treatment, respectively. Analyzed RO supernatants: see Figure 4K. Analyzed ROC supernatants: CLN2-1 $n = 7–19$, 5 RoC; CLN2-2 $n = 9–15$, 4 RoC; CTRL1 $n = 25$, 7 RoC; CTRL2 $n = 27$, 7 RoC. Scale: log10.
Values and dots are mean ± SEM. Scale bars: (B, C) 100 μm, (D) 50 μm. Hoechst: blue. Tx: treatment. $^*p < 0.05$, $^{**}p < 0.01$, $^{***}p < 0.001$, $^{****}p < 0.0001$.

Early pathomechanisms of cone photoreceptor degeneration might involve autophagy and imbalance of cell metabolism

Retinal degeneration in patients with CLN2 first affects the macula and predominantly cone photoreceptors.[18] DGE analysis of a scRNA-seq dataset derived from CLN2 ROs identified a substantial dysregulation in ribosomal genes, mitochondrial structure, and respiration in cones, suggesting an ongoing impairment of translational and mitochondrial function. This is in line with previous reports of mitochondrial fragmentation observed in CLN2 patient fibroblasts.[56] Moreover, a recent overlay of independent proteomic analyses of CLN1-4 postmortem tissues and mouse models suggested alterations in OXPHOS and mitochondrial function,[44] which are potential outcomes of autophagy disruption.[57] Interestingly, dysregulation of autophagy seems to be a common mechanism in several NCLs.[58–61] In CLN2 patient fibroblasts, reactive oxygen species (ROS) generation, caused by a defective autophagy, was associated with upregulation of the Akt/mTOR pathway that, in turn, further exacerbated autophagy disruption.[61]

Multi-omics analyses on CLN1-4 tissues identified RICTOR, a subunit of mTOR complex 2, as the upstream regulator of proteomic dysregulation, including altered OXPHOS and mitochondrial dysfunction, suggesting an involvement of the mTOR pathway in different NCLs.[44] In our study, CLN2 cones displayed an increased expression of *RICTOR* as well as dysregulation of 24 out of the 66 genes that have previously been associated with the regulatory network of RICTOR, mitochondrial structure, and OXPHOS.[44] Finally, autophagy impairment has been linked to p53 pathway upregulation, which results in decreased protein synthesis and senescence-like growth-arrested state.[62] In line with that, our iRegulon analysis identified TP53 (p53 protein), commonly associated with cell cycle and protein and ribosome biosynthesis,[42,43] as the most enriched master TF in the DGE of cones. Indeed, among the dysregulated genes in cones, we identified several proteins involved in translation (*RPS*s and *RPL*s genes) that are regulated by TP53.

Altogether, these findings indicate a pathomechanism in which TPP1 deficiency results in the failed digestion of SCMAS, leading to autophagy disruption, dysfunctional mitochondria, and subsequent increase of ROS generation influencing AKT/mTOR and p53 pathways. This overall disturbance of anabolic functions (protein, lipid, and ribosome synthesis and OXPHOS) could potentially impact cone cell survival.[63] Although investigation of the molecular pathway(s) that link TPP1 deficiency with autophagy was beyond the scope of this article, we believe that future investigation of autophagy pathway status as well as Akt/mTOR and p53 activity in CLN2 cone cells might provide further insights into the pathophysiology of CLN2 disease in the retina.

AAV9.hCLN2 therapy is a potential treatment for patients with CLN2

In 2017, cerliponase alfa, a recombinant form of human TPP1, became the first Food and Drug Administration-approved ERT for CLN2 disease. Cerliponase alfa, administered to the cerebrospinal fluid by intraventricular infusion every 2 weeks, resulted in a decrease of motor and language function decline.[9] However, a therapeutic effect on CLN2 retinal degeneration has not been reported to date. Periodic intravitreal injections of a recombinant human TPP1 protein resulted in inhibition of retinal degeneration and preserved retinal function in a CLN2 canine model.[15,16] However, an ERT approach for CLN2 would require biweekly life-long administrations of TPP1 both to the retina and the brain, resulting in a significant reduction of the quality of life of patients and an increased risk of adverse reactions (infections and allergies).

In the recent years, AAV-based gene therapies have emerged as successful one-time strategies for the treatment of genetic diseases. In a CLN2 canine model, intravitreal delivery of AAV2-TPP1 succeeded not only in preserving retinal structure and function but also in inhibiting the formation of autofluorescent storage bodies.[17] AAV9 has been shown to successfully transduce photoreceptors, RPE cells, and Müller glia cells in mouse models[64] and cones in non-human primates[65]; however, it showed low transduction efficiency for hiPSC-derived ROs and RPE cells.[29,66] ROs and the RoC have demonstrated to be suitable models to screen the efficiency and tropism of AAV-based gene delivery.[29] In the present study, AAV9.hCLN2 was added to the culture medium of ROs and the organoid compartment of the RoC acting as *in vitro* analogy of a subretinal injection, the preferred route of administration to obtain the optimal photoreceptor transduction. scRNA-seq showed that AAV9.hCLN2 was able to transduce all retinal cell types and was particularly efficient in transducing horizontal cells and cone and rod photoreceptors.[64,65] Interestingly, AAV9.hCLN2 was able to transduce 11%–18% of amacrine cells that are located in the innermost layer of the ROs *in vitro* and in one of the innermost layers of the human retina *in vivo*, suggesting that subretinal injection in patient eyes might lead to transduction of all retinal layers. In our study, immunofluorescence analyses confirmed that TPP1 transgenic protein was expressed in all retinal layers, with peaks at the photoreceptor layer. The transduction efficiency was higher in organoids treated at younger age (days 88 and 123), containing a higher number of proliferating progenitors, than in mature ROs (day 260). Moreover, AAV9.hCLN2 efficiently transduced RPE cells, proving the ability of this gene therapy product to reach all retinal cell types.

In AAV9.hCLN2-treated CLN2 ROs, SCMAS accumulation was efficiently obliterated in a dose-dependent way. Interestingly, SCMAS deposits decreased both when organoids were treated before the onset of accumulation (day 88) and when the accumulations were already present (days 123 and 260). This indicates that treatment with AAV9.hCLN2 is not only able to prevent but also to attenuate or even completely revert the CLN2 disease phenotype according to the organoid age and the disease advancement at which the treatment is performed. Due to the rather late appearance of the first ocular symptoms in patients with CLN2, this property might be particularly promising for clinical applications involving AAV9.hCLN2 gene therapy.

A significant reduction of SCMAS accumulation was also achieved in CLN2 ROs treated with the lowest AAV9.hCLN2 dose that only led to the transduction of a limited number of cells. TPP1 is a secreted protein that can be taken up through the mannose-6-phosphate receptor and targeted to the lysosomes.[67,68] Analysis of the supernatant of both control and

AAV-treated CLN2 organoids revealed the presence of secreted TPP1. It is likely that the secreted transgenic protein is internalized by neighboring cells that were not transduced by the AAV9.hCLN2 vector and cross-correct the phenotype. The capability of TPP1 to rescue the disease phenotype in surrounding cells and tissue is particularly important in a clinical context.

The RoC technology enables recapitulation of a high level of tissue complexity that can be invaluable for drug testing and pharmacological investigations, due to its ability to combine all main retinal cell types and to recapitulate retinal physiological tissue structure, compartmentalization, and function.[28,29] Moreover, we would like to highlight that the RoC provides the possibility of accurate therapy simulation by allowing (1) a one-time application of the vector without further requirement of media change to dilute or remove the remaining vectors; (2) measurement of secreted TPP1 protein in a small retinal compartment with a physiologically relevant cell-to-medium ratio, thanks to compartmentalization; and (3) a faithful co-culture of RPE and RO, which in turn allows modeling the effect of RPE and retinal cells being transduced by the viral vector in terms of dosage as well as in terms of paracrine and *trans*-tissue effects. Subretinal-like administration of AAV9.hCLN2 to CLN2 RoCs led to a more efficient TPP1 transgene expression in the RO tissue and in the supernatant of the RoC in comparison with off-chip RO culture. This is possibly due to the *in vivo*-like compartmentalization in the RoC preventing non-physiological dilution of secreted factors in the large media volumes used in static culture. A comparison between the RoC and a 384-well culture with comparable volumes supports the hypothesis that additional effects such as the auto- or paracrine secretion and uptake of TPP1 between cells could have contributed to this increase. All these factors could be of benefit for dose finding and pharmacokinetic studies as demonstrated by the significant difference in dose response between RoCs and ROs. Importantly, the approach we established, moving from high-throughput, highly standardized cell monolayer cultures to 3D organoids and finally to lower throughput, high-complexity OoC models, could serve as a blueprint for gene therapy development that can potentially be transferred to other retinal or even non-retinal diseases.[26]

In conclusion, this study provides human-relevant CLN2 disease models as well as insights into CLN2 retinal pathology and demonstrates the reversal of histopathological hallmarks via AAV gene therapy. Consequently, this work sets the ground for a successful clinical application aiming to preserve vision in CLN2-affected children.

Limitations of the study

The cell composition and function of matured ROs resemble that of late fetal stages; thus, they do not fully recapitulate the final stage of disease progression such as retinal degeneration yet. Furthermore, while we demonstrate the impairment of translational and mitochondrial function in CLN2 cone photoreceptors, the exact molecular mechanisms leading from TPP1 mutation to autophagy disruption and cone degeneration still must be uncovered. Finally, to assess AAV safety and prevent toxicity associated with immune system rejection, future studies should incorporate immune cells.

RESOURCE AVAILABILITY

Lead contact

Further information and requests for resources and reagents should be directed to and will be fulfilled by the lead contact, Kevin Achberger (kevin.achberger@uni-tuebingen.de).

Materials availability

All unique/stable reagents generated in this study are available from the lead contact with a completed materials transfer agreement.

Data and code availability

- scRNA-seq data have been deposited at the NCBI Gene Expression Omnibus database as GSE244262, RNA-seq data have been deposited at the NCBI Gene Expression Omnibus database as GSE297954 and are publicly available as of the date of publication, respectively.
- All original code has been deposited at https://github.com/kachberger/Corti_et_al_TPP1 and is publicly available as of the date of publication.
- Any additional information required to reanalyze the data reported in this paper is available from the lead contact upon request.

ACKNOWLEDGMENTS

This work was funded by REGENXBIO, Inc. The confocal platform was kindly provided by Melanie Philipp and Martin Burkhalter and was co-funded by a grant from the German Research Foundation, Germany (grant no. INST 37/1171-1 FUGG). NGS methods were performed with the support of the German Research Fundation-funded NGS Competence Center Tübingen (INST 37/1049-1). We would like to acknowledge Michaela Pogoda for her assistance in organizing the RNA sequencing experiment and Jakob Admard for the processing of the RNA sequencing data.

AUTHOR CONTRIBUTIONS

Conceptualization, S.C., P. Loskill, A.K., K.H.K., N.B., S.L., N.P., and K.A.; methodology, S.C., K.M., A.B., A.B.V., K.H.K., M.H., K.A., V.C., N.P., D.S., K.D., S.W., M.B., P. Lopatta, A.T., S.B., M.U., and A.K.; investigation, S.C., K.H.K., R.M.R., C.B.T., K.M., A.B., A.B.V., and V.C.; writing, S.C., K.A., K.H.K., P. Loskill, S.L., and A.B.; single-cell RNA sequencing analysis and bioinformatics, K.A., V.C., and T.C.; resources, K.H.K., P. Loskill, and S.L.; supervision, S.L., K.H.K., M.U., and K.A.

DECLARATION OF INTERESTS

K.H.K., C.B.T., M.H., R.M.R., T.C., and N.B. are current or previous employees of REGENXBIO, Inc. K.H.K. and N.B. are inventors on patent applications related to AAV9.hCLN2 vector delivery methods. N.B. is an inventor on patent applications related to AAV formulations. K.A., S.L., and P. Loskill hold a patent on the RoC technology.

STAR★METHODS

Detailed methods are provided in the online version of this paper and include the following:

- KEY RESOURCES TABLE
- EXPERIMENTAL MODEL AND STUDY PARTICIPANT DETAILS
- METHOD DETAILS
  - iPSC culture
  - Retinal organoid culture
  - Differentiation and culture of retinal pigment epithelial cells
  - RoC fabrication
  - RoC culture
  - AAV vectors
  - AAV treatment
  - CRISPR/Cas9 correction of *TPP1* mutation
  - Overexpression of TPP1 cDNA and NMD inhibition

- Western Blot
- Immunohistochemistry
- RO autofluorescence
- Microscopy and image processing
- Transmission electron microscopy and analysis
- TPP1 quantification in supernatants
- Gene expression analysis via RT-qPCR
- RNA sequencing
- 10X genomics single-cell RNA sequencing
- QUANTIFICATION AND STATISTICAL ANALYSIS
  - Image analysis
  - RNA sequencing analysis
  - Gene ontology and semantic analysis
  - Single-cell sequencing data analysis
  - Differential gene expression (DGE) analysis
  - Gene set enrichment analysis (GSEA)
  - iRegulon analysis
  - Scoring of RICTOR targets obtained from Kline et al
- QUANTIFICATION AND STATISTICAL ANALYSIS

SUPPLEMENTAL INFORMATION

Supplemental information can be found online at https://doi.org/10.1016/j.xcrm.2025.102244.

Received: October 4, 2023
Revised: March 10, 2025
Accepted: June 26, 2025
Published: July 23, 2025

REFERENCES

1. Sleat, D.E., Donnelly, R.J., Lackland, H., Liu, C.G., Sohar, I., Pullarkat, R. K., and Lobel, P. (1997). Association of mutations in a lysosomal protein with classical late-infantile neuronal ceroid lipofuscinosis. Science *277*, 1802–1805. https://doi.org/10.1126/SCIENCE.277.5333.1802.
2. Rawlings, N.D., and Barrett, A.J. (1999). Tripeptidyl-peptidase I is apparently the CLN2 protein absent in classical late-infantile neuronal ceroid lipofuscinosis. Biochim. Biophys. Acta *1429*, 496–500. https://doi.org/10.1016/S0167-4838(98)00238-6.
3. Palmer, D.N., Martinus, R.D., Cooper, S.M., Midwinter, G.G., Reid, J.C., and Jolly, R.D. (1989). Ovine ceroid lipofuscinosis. The major lipopigment protein and the lipid-binding subunit of mitochondrial ATP synthase have the same NH2-terminal sequence. J. Biol. Chem. *264*, 5736–5740.
4. Palmer, D.N., Fearnley, I.M., Medd, S.M., Walker, J.E., Martinus, R.D., Bayliss, S.L., Hall, N.A., Lake, B.D., Wolfe, L.S., and Jolly, R.D. (1989). Lysosomal storage of the DCCD reactive proteolipid subunit of mitochondrial ATP synthase in human and ovine ceroid lipofuscinoses. Adv. Exp. Med. Biol. *266*, 211–223. https://doi.org/10.1007/978-1-4899-5339-1_15.
5. Ezaki, J., Takeda-Ezaki, M., and Kominami, E. (2000). Tripeptidyl peptidase I, the late infantile neuronal ceroid lipofuscinosis gene product, initiates the lysosomal degradation of subunit c of ATP synthase. J. Biochem. *128*, 509–516. https://doi.org/10.1093/oxfordjournals.jbchem.a022781.
6. Haltia, M. (2003). The neuronal ceroid-lipofuscinoses. J. Neuropathol. Exp. Neurol. *62*, 1–13. https://doi.org/10.1093/JNEN/62.1.1.
7. Kohlschütter, A., Schulz, A., Bartsch, U., and Storch, S. (2019). Current and Emerging Treatment Strategies for Neuronal Ceroid Lipofuscinoses. CNS Drugs *33*, 315–325. https://doi.org/10.1007/S40263-019-00620-8.
8. Kovacs, K.D., Patel, S., Orlin, A., Kim, K., Van Everen, S., Conner, T., Sondhi, D., Kaminsky, S.M., D'Amico, D.J., Crystal, R.G., and Kiss, S. (2020). Symmetric Age Association of Retinal Degeneration in Patients with CLN2-Associated Batten Disease. Ophthalmol. Retina *4*, 728–736. https://doi.org/10.1016/j.oret.2020.01.011.
9. Schulz, A., Ajayi, T., Specchio, N., de Los Reyes, E., Gissen, P., Ballon, D., Dyke, J.P., Cahan, H., Slasor, P., Jacoby, D., et al. (2018). Study of Intraventricular Cerliponase Alfa for CLN2 Disease. N. Engl. J. Med. *378*, 1898–1907. https://doi.org/10.1056/nejmoa1712649.
10. Whiting, R.E.H., Narfström, K., Yao, G., Pearce, J.W., Coates, J.R., Castaner, L.J., Jensen, C.A., Dougherty, B.N., Vuillemenot, B.R., Kennedy, D., et al. (2014). Enzyme replacement therapy delays pupillary light reflex deficits in a canine model of late infantile neuronal ceroid lipofuscinosis. Exp. Eye Res. *125*, 164–172. https://doi.org/10.1016/J.EXER.2014.06.008.
11. Sleat, D.E., Wiseman, J.A., El-Banna, M., Kim, K.H., Mao, Q., Price, S., Macauley, S.L., Sidman, R.L., Shen, M.M., Zhao, Q., et al. (2004). A mouse model of classical late-infantile neuronal ceroid lipofuscinosis based on targeted disruption of the CLN2 gene results in a loss of tripeptidyl-peptidase I activity and progressive neurodegeneration. J. Neurosci. *24*, 9117–9126. https://doi.org/10.1523/JNEUROSCI.2729-04.2004.
12. Geraets, R.D., Langin, L.M., Cain, J.T., Parker, C.M., Beraldi, R., Kovacs, A.D., Weimer, J.M., and Pearce, D.A. (2017). A tailored mouse model of CLN2 disease: A nonsense mutant for testing personalized therapies. PLoS One *12*, e0176526. https://doi.org/10.1371/JOURNAL.PONE.0176526.
13. Chang, M., Cooper, J.D., Sleat, D.E., Cheng, S.H., Dodge, J.C., Passini, M.A., Lobel, P., and Davidson, B.L. (2008). Intraventricular enzyme replacement improves disease phenotypes in a mouse model of late infantile neuronal ceroid lipofuscinosis. Mol. Ther. *16*, 649–656. https://doi.org/10.1038/MT.2008.9.
14. Katz, M.L., Coates, J.R., Cooper, J.J., O'Brien, D.P., Jeong, M., and Narfström, K. (2008). Retinal pathology in a canine model of late infantile neuronal ceroid lipofuscinosis. Investig. Ophthalmol. Vis. Sci. *49*, 2686–2695. https://doi.org/10.1167/IOVS.08-1712.
15. Whiting, R.E.H., Robinson Kick, G., Ota-Kuroki, J., Lim, S., Castaner, L.J., Jensen, C.A., Kowal, J., Nguyen, A., Corado, C., O'Neill, C.A., and Katz, M. L. (2020). Intravitreal enzyme replacement inhibits progression of retinal degeneration in canine CLN2 neuronal ceroid lipofuscinosis. Exp. Eye Res. *198*, 108135. https://doi.org/10.1016/j.exer.2020.108135.
16. Whiting, R.E.H., Pearce, J.W., Vansteenkiste, D.P., Bibi, K., Lim, S., Robinson Kick, G., Castaner, L.J., Sinclair, J., Chandra, S., Nguyen, A., et al. (2020). Intravitreal enzyme replacement preserves retinal structure and function in canine CLN2 neuronal ceroid lipofuscinosis. Exp. Eye Res. *197*, 108130. https://doi.org/10.1016/j.exer.2020.108130.
17. Kick, G.R., Whiting, R.E.H., Ota-Kuroki, J., Castaner, L.J., Morgan-Jack, B., Sabol, J.C., Meiman, E.J., Ortiz, F., and Katz, M.L. (2023). Intravitreal gene therapy preserves retinal function in a canine model of CLN2 neuronal ceroid lipofuscinosis. Exp. Eye Res. *226*, 109344. https://doi.org/10.1016/J.EXER.2022.109344.
18. Thompson, D.A., Handley, S.E., Henderson, R.H., Marmoy, O.R., and Gissen, P. (2021). An ERG and OCT study of neuronal ceroid lipofuscinosis CLN2 Battens retinopathy. Eye *35*, 2438–2448. https://doi.org/10.1038/s41433-021-01594-y.
19. Orlin, A., Sondhi, D., Witmer, M.T., Wessel, M.M., Mezey, J.G., Kaminsky, S.M., Hackett, N.R., Yohay, K., Kosofsky, B., Souweidane, M.M., et al. (2013). Spectrum of Ocular Manifestations in CLN2-Associated Batten (Jansky-Bielschowsky) Disease Correlate with Advancing Age and Deteriorating Neurological Function. PLoS One *8*, e73128. https://doi.org/10.1371/JOURNAL.PONE.0073128.
20. Zhong, X., Gutierrez, C., Xue, T., Hampton, C., Vergara, M.N., Cao, L.H., Peters, A., Park, T.S., Zambidis, E.T., Meyer, J.S., et al. (2014). Generation of three-dimensional retinal tissue with functional photoreceptors from human iPSCs. Nat. Commun. *5*, 4047. https://doi.org/10.1038/ncomms5047.
21. Ohlemacher, S.K., Iglesias, C.L., Sridhar, A., Gamm, D.M., and Meyer, J.S. (2015). Generation of highly enriched populations of optic vesicle-like retinal cells from human pluripotent stem cells. Curr. Protoc. Stem Cell Biol. *32*, 1H.8.1–20. https://doi.org/10.1002/9780470151808.sc01h08s32.
22. Sridhar, A., Hoshino, A., Finkbeiner, C.R., Chitsazan, A., Dai, L., Haugan, A.K., Eschenbacher, K.M., Jackson, D.L., Trapnell, C., Bermingham-McDonogh, O., et al. (2020). Single-Cell Transcriptomic Comparison of

CellPress
OPEN ACCESS

Human Fetal Retina, hPSC-Derived Retinal Organoids, and Long-Term Retinal Cultures. Cell Rep. *30*, 1644–1659.e4. https://doi.org/10.1016/J.CELREP.2020.01.007.

23. Lane, A., Jovanovic, K., Shortall, C., Ottaviani, D., Panes, A.B., Schwarz, N., Guarascio, R., Hayes, M.J., Palfi, A., Chadderton, N., et al. (2020). Modeling and Rescue of RP2 Retinitis Pigmentosa Using iPSC-Derived Retinal Organoids. Stem Cell Rep. *15*, 67–79. https://doi.org/10.1016/j.stemcr.2020.05.007.
24. Kruczek, K., Qu, Z., Gentry, J., Fadl, B.R., Gieser, L., Hiriyanna, S., Batz, Z., Samant, M., Samanta, A., Chu, C.J., et al. (2021). Gene Therapy of Dominant CRX-Leber Congenital Amaurosis using Patient Stem Cell-Derived Retinal Organoids. Stem Cell Rep. *16*, 252–263. https://doi.org/10.1016/j.stemcr.2020.12.018.
25. Rodrigues, A., Slembrouck-Brec, A., Nanteau, C., Terray, A., Tymoshenko, Y., Zagar, Y., Reichman, S., Xi, Z., Sahel, J.A., Fouquet, S., et al. (2022). Modeling PRPF31 retinitis pigmentosa using retinal pigment epithelium and organoids combined with gene augmentation rescue22. NPJ Regen. Med. *7*, 139. https://doi.org/10.1038/s41536-022-00235-6.
26. Loskill, P., Hardwick, R.N., and Roth, A. (2021). Challenging the pipeline. Stem Cell Rep. *16*, 2033–2037. https://doi.org/10.1016/J.STEMCR.2021.08.004.
27. Ingber, D.E. (2022). Human organs-on-chips for disease modelling, drug development and personalized medicine. Nat. Rev. Genet. *23*, 467–491. https://doi.org/10.1038/S41576-022-00466-9.
28. Achberger, K., Probst, C., Haderspeck, J., Bolz, S., Rogal, J., Chuchuy, J., Nikolova, M., Cora, V., Antkowiak, L., Haq, W., et al. (2019). Merging organoid and organ-on-a-chip technology to generate complex multi-layer tissue models in a human retina-on-a-chip platform. eLife *8*, e46188. https://doi.org/10.7554/eLife.46188.
29. Achberger, K., Cipriano, M., Düchs, M.J., Schön, C., Michelfelder, S., Stierstorfer, B., Lamla, T., Kauschke, S.G., Chuchuy, J., Roosz, J., et al. (2021). Human stem cell-based retina on chip as new translational model for validation of AAV retinal gene therapy vectors. Stem Cell Rep. *16*, 2242–2256. https://doi.org/10.1016/j.stemcr.2021.08.008.
30. Gardner, E., Bailey, M., Schulz, A., Aristorena, M., Miller, N., and Mole, S.E. (2019). Mutation update: Review of TPP1 gene variants associated with neuronal ceroid lipofuscinosis CLN2 disease. Hum. Mutat. *40*, 1924–1938. https://doi.org/10.1002/humu.23860.
31. Ma, L., Prada, A.M., Schmidt, M., and Morrow, E.M. (2021). Generation of pathogenic TPP1 mutations in human stem cells as a model for neuronal ceroid lipofuscinosis type 2 disease. Stem Cell Res. *53*, 102323. https://doi.org/10.1016/J.SCR.2021.102323.
32. Worgall, S., Kekatpure, M.V., Heier, L., Ballon, D., Dyke, J.P., Shungu, D., Mao, X., Kosofsky, B., Kaplitt, M.G., Souweidane, M.M., et al. (2007). Neurological deterioration in late infantile neuronal ceroid lipofuscinosis. Neurology *69*, 521–535. https://doi.org/10.1212/01.WNL.0000267885.47092.40.
33. Cowan, C.S., Renner, M., De Gennaro, M., Gross-Scherf, B., Goldblum, D., Hou, Y., Munz, M., Rodrigues, T.M., Krol, J., Szikra, T., et al. (2020). Cell Types of the Human Retina and Its Organoids at Single-Cell Resolution. Cell *182*, 1623–1640.e34. https://doi.org/10.1016/J.CELL.2020.08.013.
34. Palmer, D.N., Fearnley, I.M., Walker, J.E., Hall, N.A., Lake, B.D., Wolfe, L. S., Haltia, M., Martinus, R.D., and Jolly, R.D. (1992). Mitochondrial ATP synthase subunit c storage in the ceroid-lipofuscinoses (Batten disease). Am. J. Med. Genet. *42*, 561–567. https://doi.org/10.1002/AJMG.1320420428.
35. Micsenyi, M.C., Sikora, J., Stephney, G., Dobrenis, K., and Walkley, S.U. (2013). Lysosomal membrane permeability stimulates protein aggregate formation in neurons of a lysosomal disease. J. Neurosci. *33*, 10815–10827. https://doi.org/10.1523/JNEUROSCI.0987-13.2013.
36. Goebel, H.H., Zeman, W., and Damaske, E. (1977). An ultrastructural study of the retina in the Jansky-Bielschowsky type of neuronal ceroid-lipofuscinosis. Am. J. Ophthalmol. *83*, 70–79. https://doi.org/10.1016/0002-9394(77)90194-5.
37. Traboulsi, E.I., Green, W.R., Luckenbach, M.W., and de la Cruz, Z.C. (1987). Neuronal ceroid lipofuscinosis. Ocular histopathologic and electron microscopic studies in the late infantile, juvenile, and adult forms. Graefes Arch. Clin. Exp. Ophthalmol. *225*, 391–402. https://doi.org/10.1007/BF02334164.
38. Lojewski, X., Staropoli, J.F., Biswas-legrand, S., Simas, A.M., Haliw, L., Selig, M.K., Coppel, S.H., Goss, K.A., Petcherski, A., Chandrachud, U., et al. (2014). Human iPSC models of neuronal ceroid lipofuscinosis capture distinct effects of TPP1 and CLN3 mutations on the endocytic pathway. Hum. Mol. Genet. *23*, 2005–2022. https://doi.org/10.1093/HMG/DDT596.
39. Passini, M.A., Dodge, J.C., Bu, J., Yang, W., Zhao, Q., Sondhi, D., Hackett, N.R., Kaminsky, S.M., Mao, Q., Shihabuddin, L.S., et al. (2006). Intracranial delivery of CLN2 reduces brain pathology in a mouse model of classical late infantile neuronal ceroid lipofuscinosis. J. Neurosci. *26*, 1334–1342. https://doi.org/10.1523/JNEUROSCI.2676-05.2006.
40. Arsenio-Nunes, M.L., Goutières, F., and Aicardi, J. (1981). An ultramicroscopic study of skin and conjunctival biopsies in chronic neurological disorders of childhood. Ann. Neurol. *9*, 163–173. https://doi.org/10.1002/ANA.410090210.
41. Weleber, R.G. (1998). The dystrophic retina in multisystem disorders: the electroretinogram in neuronal ceroid lipofuscinoses. Eye *12*, 580–590. https://doi.org/10.1038/EYE.1998.148.
42. Levine, A.J. (1997). p53, the cellular gatekeeper for growth and division. Cell *88*, 323–331. https://doi.org/10.1016/S0092-8674(00)81871-1.
43. Budde, A., and Grummt, I. (1999). p53 represses ribosomal gene transcription. Oncogene *18*, 1119–1124. https://doi.org/10.1038/sj.onc.1202402.
44. Kline, R.A., Wishart, T.M., Mills, K., and Heywood, W.E. (2020). Applying modern Omic technologies to the Neuronal Ceroid Lipofuscinoses. Biochim. Biophys. Acta, Mol. Basis Dis. *1866*, 165498. https://doi.org/10.1016/J.BBADIS.2019.06.012.
45. Buss, N., Lanigan, L., Zeller, J., Cissell, D., Metea, M., Adams, E., Higgins, M., Kim, K.H., Budzynski, E., Yang, L., et al. (2022). Characterization of AAV-mediated dorsal root ganglionopathy. Mol. Ther. Methods Clin. Dev. *24*, 342–354. https://doi.org/10.1016/j.omtm.2022.01.013.
46. Takahashi, K., Eultgen, E.M., Wang, S.H., Rensing, N.R., Nelvagal, H.R., Dearborn, J.T., Danos, O., Buss, N., Sands, M.S., Wong, M., and Cooper, J.D. (2023). Gene therapy ameliorates spontaneous seizures associated with cortical neuron loss in a Cln2R207X mouse model. J. Clin. Investig. *133*, e165908. https://doi.org/10.1172/JCI165908.
47. Ohlemacher, S.K., Iglesias, C.L., Sridhar, A., Gamm, D.M., and Meyer, J.S. (2015). Generation of highly enriched populations of optic vesicle-like retinal cells from human pluripotent stem cells. Curr. Protoc. Stem Cell Biol. *32*, 1h.8.1–1h.8.20. https://doi.org/10.1002/9780470151808.sc01h08s32.
48. Goebel, H.H. (1995). Topical Review: The Neuronal Ceroid-Lipofuscinoses. J. Child Neurol. *10*, 424–437. https://doi.org/10.1177/088307389501000602.
49. Elleder, M., Sokolová, J., and Hrebícek, M. (1997). Follow-up study of subunit c of mitochondrial ATP synthase (SCMAS) in Batten disease and in unrelated lysosomal disorders. Acta Neuropathol. *93*, 379–390. https://doi.org/10.1007/S004010050629.
50. Sleat, D.E., Tannous, A., Sohar, I., Wiseman, J.A., Zheng, H., Qian, M., Zhao, C., Xin, W., Barone, R., Sims, K.B., et al. (2017). Proteomic analysis of brain and cerebrospinal fluid from the three major forms of neuronal ceroid lipofuscinosis reveals potential biomarkers. J. Proteome Res. *16*, 3787–3804. https://doi.org/10.1021/acs.jproteome.7b00460.
51. Kovacs, K.D., Orlin, A., Sondhi, D., Kaminsky, S.M., D'amico, D.J., Crystal, R.G., and Kiss, S. (2021). Automated Retinal Layer Segmentation in CLN2-Associated Disease: Commercially Available Software Characterizing a

Progressive Maculopathy. Transl. Vis. Sci. Technol. *10*, 23. https://doi.org/10.1167/TVST.10.8.23.

52. Kominami, E., Ezaki, J., Muno, D., Ishido, K., Ueno, T., and Wolfe, L.S. (1992). Specific storage of subunit c of mitochondrial ATP synthase in lysosomes of neuronal ceroid lipofuscinosis (batten's disease). J. Biochem. *111*, 278–282. https://doi.org/10.1093/oxfordjournals.jbchem.a123749.
53. Ezaki, J., Wolfe, L.S., Higuti, T., Ishidoh, K., and Kominami, E. (1995). Specific Delay of Degradation of Mitochondrial ATP Synthase Subunit c in Late Infantile Neuronal Ceroid Lipofuscinosis (Batten Disease). J. Neurochem. *64*, 733–741. https://doi.org/10.1046/j.1471-4159.1995.64020733.x.
54. Sima, N., Li, R., Huang, W., Xu, M., Beers, J., Zou, J., Titus, S., Ottinger, E. A., Marugan, J.J., Xie, X., and Zheng, W. (2018). Neural stem cells for disease modeling and evaluation of therapeutics for infantile (CLN1/PPT1) and late infantile (CLN2/TPP1) neuronal ceroid lipofuscinoses. Orphanet J. Rare Dis. *13*, 54. https://doi.org/10.1186/S13023-018-0798-2.
55. Mahmood, F., Fu, S., Cooke, J., Wilson, S.W., Cooper, J.D., and Russell, C. (2013). A zebrafish model of CLN2 disease is deficient in tripeptidyl peptidase 1 and displays progressive neurodegeneration accompanied by a reduction in proliferation. Brain *136*, 1488–1507. https://doi.org/10.1093/BRAIN/AWT043.
56. Van Beersel, G., Tihon, E., Demine, S., Hamer, I., Jadot, M., and Arnould, T. (2013). Different molecular mechanisms involved in spontaneous and oxidative stress-induced mitochondrial fragmentation in tripeptidyl peptidase-1 (TPP-1)-deficient fibroblasts. Biosci. Rep. *33*, e00023–e00258. https://doi.org/10.1042/BSR20120104.
57. Wu, J.J., Quijano, C., Chen, E., Liu, H., Cao, L., Fergusson, M.M., Rovira, I. I., Gutkind, S., Daniels, M.P., Komatsu, M., and Finkel, T. (2009). Mitochondrial dysfunction and oxidative stress mediate the physiological impairment induced by the disruption of autophagy. Aging *1*, 425–437. https://doi.org/10.18632/AGING.100038.
58. Seranova, E., Connolly, K.J., Zatyka, M., Rosenstock, T.R., Barrett, T., Tuxworth, R.I., and Sarkar, S. (2017). Dysregulation of autophagy as a common mechanism in lysosomal storage diseases. Essays Biochem. *61*, 733–749. https://doi.org/10.1042/EBC20170055.
59. Mukherjee, A.B., Appu, A.P., Sadhukhan, T., Casey, S., Mondal, A., Zhang, Z., and Bagh, M.B. (2019). Emerging new roles of the lysosome and neuronal ceroid lipofuscinoses. Mol. Neurodegener. *14*, 14–23. https://doi.org/10.1186/S13024-018-0300-6.
60. Brandenstein, L., Schweizer, M., Sedlacik, J., Fiehler, J., and Storch, S. (2016). Lysosomal dysfunction and impaired autophagy in a novel mouse model deficient for the lysosomal membrane protein Cln7. Hum. Mol. Genet. *25*, 777–791. https://doi.org/10.1093/HMG/DDV615.
61. Vidal-Donet, J.M., Cárcel-Trullols, J., Casanova, B., Aguado, C., and Knecht, E. (2013). Alterations in ROS activity and lysosomal pH account for distinct patterns of macroautophagy in LINCL and JNCL fibroblasts. PLoS One *8*, e55526. https://doi.org/10.1371/JOURNAL.PONE.0055526.
62. Kang, H.T., Lee, K.B., Kim, S.Y., Choi, H.R., and Park, S.C. (2011). Autophagy impairment induces premature senescence in primary human fibroblasts. PLoS One *6*, e23367. https://doi.org/10.1371/JOURNAL.PONE.0023367.
63. Li, Z.y., Yang, Y., Ming, M., and Liu, B. (2011). Mitochondrial ROS generation for regulation of autophagic pathways in cancer. Biochem. Biophys. Res. Commun. *414*, 5–8. https://doi.org/10.1016/j.bbrc.2011.09.046.
64. Lei, B., Zhang, K., Yue, Y., Ghosh, A., and Duan, D. (2009). Adeno-associated virus serotype-9 efficiently transduces the retinal outer plexiform layer. Mol. Vis. *15*, 1374–1382.
65. Vandenberghe, L.H., Bell, P., Maguire, A.M., Xiao, R., Hopkins, T.B., Grant, R., Bennett, J., and Wilson, J.M. (2013). AAV9 Targets Cone Photoreceptors in the Nonhuman Primate Retina. PLoS One *8*, e53463. https://doi.org/10.1371/JOURNAL.PONE.0053463.
66. Garita-Hernandez, M., Routet, F., Guibbal, L., Khabou, H., Toualbi, L., Riancho, L., Reichman, S., Duebel, J., Sahel, J.A., Goureau, O., and Dalkara, D. (2020). AAV-mediated gene delivery to 3D retinal organoids derived from human induced pluripotent stem cells. Int. J. Mol. Sci. *21*, 994. https://doi.org/10.3390/ijms21030994.
67. Wujek, P., Kida, E., Walus, M., Wisniewski, K.E., and Golabek, A.A. (2004). N-glycosylation is crucial for folding, trafficking, and stability of human tripeptidyl-peptidase I. J. Biol. Chem. *279*, 12827–12839. https://doi.org/10.1074/JBC.M313173200.
68. Baranzehi, T., Kordi-Tamandani, D.M., Najafi, M., Khajeh, A., and Schmidts, M. (2022). Identification of a TPP1 Q278X Mutation in an Iranian Patient with Neuronal Ceroid Lipofuscinosis 2: Literature Review and Mutations Update. J. Clin. Med. *11*, 6415. https://doi.org/10.3390/JCM11216415.
69. Frank, S., Zhang, M., Schöler, H.R., and Greber, B. (2012). Small molecule-assisted, line-independent maintenance of human pluripotent stem cells in defined conditions. PLoS One *7*, e41958. https://doi.org/10.1371/JOURNAL.PONE.0041958.
70. Pantazis, C.B., Yang, A., Lara, E., McDonough, J.A., Blauwendraat, C., Peng, L., Oguro, H., Kanaujiya, J., Zou, J., Sebesta, D., et al. (2022). A reference human induced pluripotent stem cell line for large-scale collaborative studies. Cell Stem Cell *29*, 1685–1702.e22. https://doi.org/10.1016/J.STEM.2022.11.004.
71. Yu, G., Wang, L.G., Han, Y., and He, Q.Y. (2012). ClusterProfiler: An R package for comparing biological themes among gene clusters. OMICS *16*, 284–287. https://doi.org/10.1089/OMI.2011.0118/ASSET/IMAGES/LARGE/FIGURE1.JPEG.
72. Heaton, H., Talman, A.M., Knights, A., Imaz, M., Gaffney, D.J., Durbin, R., Hemberg, M., and Lawniczak, M.K.N. (2020). Souporcell: robust clustering of single-cell RNA-seq data by genotype without reference genotypes. Nat. Methods *17*, 615–620. https://doi.org/10.1038/s41592-020-0820-1.
73. Butler, A., Hoffman, P., Smibert, P., Papalexi, E., and Satija, R. (2018). Integrating single-cell transcriptomic data across different conditions, technologies, and species. Nat. Biotechnol. *36*, 411–420. https://doi.org/10.1038/nbt.4096.
74. Janky, R., Verfaillie, A., Imrichová, H., van de Sande, B., Standaert, L., Christiaens, V., Hulselmans, G., Herten, K., Naval Sanchez, M., Potier, D., et al. (2014). iRegulon: From a Gene List to a Gene Regulatory Network Using Large Motif and Track Collections. PLoS Comput. Biol. *10*, e1003731. https://doi.org/10.1371/JOURNAL.PCBI.1003731.
75. Okita, K., Matsumura, Y., Sato, Y., Okada, A., Morizane, A., Okamoto, S., Hong, H., Nakagawa, M., Tanabe, K., Tezuka, K.I., et al. (2011). A more efficient method to generate integration-free human iPS cells. Nat. Methods *8*, 409409–412412. https://doi.org/10.1038/nmeth.1591.
76. Love, M.I., Huber, W., and Anders, S. (2014). Moderated estimation of fold change and dispersion for RNA-seq data with DESeq221. Genome Biol. *15*, 550. https://doi.org/10.1186/S13059-014-0550-8/FIGURES/9.
77. McGinnis, C.S., Murrow, L.M., and Gartner, Z.J. (2019). DoubletFinder: Doublet Detection in Single-Cell RNA Sequencing Data Using Artificial Nearest Neighbors. Cell Syst. *8*, 329–337.e4. https://doi.org/10.1016/j.cels.2019.03.003.
78. Andreatta, M., and Carmona, S.J. (2021). UCell: Robust and scalable single-cell gene signature scoring. Comput. Struct. Biotechnol. J. *19*, 3796–3798. https://doi.org/10.1016/J.CSBJ.2021.06.043.
79. Gu, Z., Eils, R., and Schlesner, M. (2016). Complex heatmaps reveal patterns and correlations in multidimensional genomic data. Bioinformatics *32*, 2847–2849. https://doi.org/10.1093/BIOINFORMATICS/BTW313.
80. Alquicira-Hernandez, J., and Powell, J.E. (2021). Nebulosa recovers single-cell gene expression signals by kernel density estimation. Bioinformatics *37*, 2485–2487. https://doi.org/10.1093/BIOINFORMATICS/BTAB003.
81. Marsh, S., Salmon, M., and Hoffman, P. (2023). samuel-marsh/scCustomize: Version 1.1.1. Zenodo https://doi.org/10.5281/ZENODO.7534950.
82. Durinck, S., Spellman, P.T., Birney, E., and Huber, W. (2009). Mapping identifiers for the integration of genomic datasets with the R/Bioconductor package biomaRt. Nat. Protoc. *4*, 1184–1191. https://doi.org/10.1038/NPROT.2009.97.

CellPress
OPEN ACCESS

STAR★METHODS

KEY RESOURCES TABLE

REAGENT or RESOURCE	SOURCE	IDENTIFIER
Antibodies		
HSP60	Abcam, UK	Cat#: ab128567; RRID:AB_11145464
LAMP1	Abcam, UK	Cat#: ab24170; RRID:AB_775978
LAMP2	Santa Cruz Biotechnology, USA	Cat#: Sc-18822; RRID:AB_626858
Recoverin	Santa Cruz Biotechnology, USA	Cat#: Sc-20353; RRID:AB_2284995
SCMAS	Abcam, UK	Cat#: ab181243; RRID:AB_2935765
TOMM20	Proteintech,USA	Cat#: 11802-1-AP; RRID:AB_2207530
TPP1	Santa Cruz Biotechnology, USA	Cat#: Sc-365838; RRID:AB_10846463
Bacterial and virus strains		
AAV9.hCLN2	Buss et al.[45]	
Chemicals, peptides, and recombinant proteins		
Matrigel hESC-qualified	BD Biosciences, USA	354277
FTDA	Frank et al.[69]	–
TrypLE	ThermoFisher Scientific	12604013
PeproGrow	Peprotech, USA	BM-hESC-500
mTESR	Stemcell Technologies, Canada	85850
Y27632 (ROCK-inhibitor)	Ascent Scientific, USA	Asc-129
Blebbistatin	Sigma-Aldrich, USA	B0560
DMEM/F12 (1:1) containing Glutamax supplement	ThermoFisher Scientific, USA	10565018
Minimum essential media-nonessential amino acids (NEAA)	ThermoFisher Scientific, USA	11140050
Antibiotics-antimycotics (AA)	ThermoFisher Scientific, USA	15240062
Growth Factor Reduced Matrigel	BD Biosciences, USA	356231
B27 (w/o vitamin A)	ThermoFisher Scientific, USA	12587010
fetal bovine serum (FBS)	Thermo Fisher Scientific, USA	A5256801
Taurine	Sigma-Aldrich, USA	T8691
Retinoic Acid	Sigma-Aldrich, USA	R2625
Laminin	Roche, Switzerland	11243217001
HyStem-C	ESI Bio, USA	#GS313F
CloneR	Stem Cell Technologies, UA	5888
Critical commercial assays		
LipidSpot™ 488 Lipid Droplet Stain	Biotium, USA	70065-T
Deposited data		
Single Cell RNAseq-Raw and analyzed Data	This Paper	GEO: GSE244262
RNAseq-Raw and analyzed Data	This Paper	GEO: GSE297954
Experimental models: Cell lines		
CTRL1	Applied StemCell Inc, USA	ASE-9202
CTRL2	ThermoFisher Scientific, USA	A18945
CTRL3	Pantazis et al.[70]	KOLF2.1J
CLN2-1	Coriell Institute, USA	GM16485
CLN2-2	Coriell Institute, USA	GM16486
Software and algorithms		
Fiji, ImageJ 1.53	ImageJ	https://imagej.net/software/fiji/

(Continued on next page)

Continued

REAGENT or RESOURCE	SOURCE	IDENTIFIER
STAR v2.7.10b	STAR	Dobin et al.,2013
DESeq2	DESeq2	Love, Huber and Anders, 2014
ClusterProfiler R package v4.6	ClusterProfiler	Yu et al., 2012[71]
RStudio 2023	Posit Software, PBC	https://posit.co/download/rstudio-desktop/
R version 4.3	R Core Team	https://cran.r-project.org/
Souporcell v2.0	Souporcell	Heaton et al., 2020[72]
Seurat 4.3	Seurat	Butler et al., 2018[73]
GraphPad Prism 9.0	GraphPad Software, USA	
iRegulon v1.3	iRegulon	Janky et al., 2014[74]
Cytoscope v3.9.1	Cytoscope	https://cytoscape.org/).

EXPERIMENTAL MODEL AND STUDY PARTICIPANT DETAILS

Three apparently healthy hiPSC lines from two independent donors, referred to as CTRL1 (male; ASE-9202, Applied StemCell Inc, USA), CTRL2 (female; A18945, ThermoFisher Scientific, USA) and CTRL3 (KOLF2.1J,[70]) were commercially purchased. CLN2 iPSC lines (GM27465*A, GM28380*B) were generated by Applied StemCell Inc. (Milpitas, USA) from two CLN2 patient fibroblasts obtained from Coriell Institute: GM16485 (referred to as CLN2-1) from female and GM16486 (CLN2-2) from male lines. CLN2-1 line harbors a C-to-T transition at nucleotide 379 in exon 4 and a C-to-T transition at nucleotide 622 in exon 6, that result in two nonsense mutations. In CLN2-2 line, a G-to-A transition at nucleotide 380 in exon 4 results in a missense mutation and a G-to-C transversion in intron 5 at nucleotide g.3556 (IVS5-1G>C). For the reprogramming of CTRL1 and CTRL2, episomal expression of OCT4, SOX2, KLF4, LIN28, L-Myc and p53 shRNA were employed following methods described previously.[75] Reprogramming of CTRL3, CLN2-1 and CLN2-2 was performed via Sendai viral vectors containing OCT4, SOX2, KL4 and c-Myc (Sendai Cyto-Tune, Thermo Fisher Scientific, USA). All lines have been tested positive for pluripotency markers and negative for mycoplasma by the distributors. Reported mutations in *CLN2* gene of patient fibroblasts were confirmed by Sanger sequencing.

METHOD DETAILS

iPSC culture

HiPSC were cultured on 6-well plates coated with Matrigel (hESC-qualified, BD Biosciences, USA) using FTDA medium,[69] as previously described.[28] All procedures were done in accordance with the Helsinki Convention and approved by the Ethical Committee of the Eberhard Karls University Tübingen (no. 678/2017BO2).

Retinal organoid culture

ROs were differentiated from hiPSCs based on a previously described protocol by Zhong et al., 2014[20,28] with additional modifications. Briefly, on d0, approximately 2 million hiPSCs were detached and dissociated using TrypLE (ThermoFisher Scientific, USA). Cells were resuspended in either PeproGrow (Peprotech, USA) or mTESR medium + mTESR Plus 5x Supplement (Stemcell Technologies, Canada). Additionally, 10 mM Y-27632 (ROCK-inhibitor, Ascent Scientific, USA) and 10 mM blebbistatin (Sigma-Aldrich, USA) were added and the cells were distributed in untreated v-shaped 96-wells (Sarstedt, Germany) at a density of 20000 cells/well. Then, the plate was centrifuged for 4 min at 400 g. On d1 and d4, the medium was replaced with N2-based neural induction medium (DMEM/F12 (1:1) containing Glutamax supplement (ThermoFisher Scientific, USA), 100X *N*-2 Supplement (ThermoFisher Scientific, USA), 1x minimum essential media-nonessential amino acids (NEAA, ThermoFisher Scientific, USA), and 1x antibiotics-antimycotics (AA, ThermoFisher Scientific, USA). On d7, the EBs were plated on 6-well plates coated with Growth Factor Reduced Matrigel (BD Biosciences, USA) at a density of 32 EBs/well, changing the medium every other day. On d16 the medium was switched to a B27-based retinal differentiation medium (BRDM) (DMEM/F12 3:1 containing 2% B27 (w/o vitamin A, ThermoFisher Scientific, USA), 1x NEAA and 1x AA). On d24, the retinal fields were detached using a cell spatula (Techno Plastic Products AG, Switzerland) and transferred to 10 cm bacterial grade petri dishes (Greiner Bio One, Germany) with BRDM. On the next day, non-retina spheres were removed and ROs were cut with microscissors. From d35 on, the medium was supplemented with 10% fetal bovine serum (FBS, Thermo Fisher Scientific, USA) and 100 μM taurine (Sigma-Aldrich, USA). Between days 70–100, the medium was further supplemented with 1 μM RA and between days 100–190 the RA concentration was reduced to 0.5 μM. After 190 days, the RA was completely removed. Additionally, during the RO differentiation, pigmented areas were manually excised from the ROs and kept in culture together with ROs for the whole duration of the differentiation.

Differentiation and culture of retinal pigment epithelial cells

RPE cells were obtained from RPE organoids according to methods adapted from Zhong et al., 2014, Ohlemacher et al., 2015 and Achberger et al., 2019.[20,28,47] For passaging, d120-150 RPE organoids were dissociated with the Neurosphere Dissociation Kit (P) (Miltenyi Biotec, Germany) according to manufacturer's instruction and seeded at the density of 75,000 cells/cm^2 in BRDM supplemented with 10% FBS, 20 mg/mL EGF (Cell Guidance Systems, United Kingdom), 20 mg/mL FGF2 (Cell Guidance Systems, United Kingdom), 2 mg/mL heparin (Sigma-Aldrich, USA), and 10 mM Y-27632 (ROCK-inhibitor, Ascent Scientific, USA). After one or two days of culture, once the cells reached confluency, the medium was changed to BRDM and replaced every other day.

RoC fabrication

Fabrication of the RoC was done according to Achberger et al.,[28] with a modified design of the top layer, allowing a 27 μL volume of the tissue compartments.

RoC culture

The culture system was prepared according to Achberger et al., 2019[28] with minor modifications. Individual chips were sterilized under the UV light for 1 h and kept in PBS to displace the air in the channels. After coating the wells with 50 μg/mL Laminin (Roche, Switzerland) for 2h, the RPEs were dissociated using Accumax at 37°C and 5% CO2 for 10–30 min. The cells were seeded at a density of 15000/well in 4.5 μL BRDM supplemented with 10% FBS, 20 mg/mL EGF (Cell Guidance Systems, United Kingdom), 20 mg/mL FGF2 (Cell Guidance Systems, United Kingdom), 2 mg/mL heparin (Sigma-Aldrich, USA), and 10 mM Y-27632 (ROCK-inhibitor, Ascent Scientific, USA). The medium was changed every day with BRDM for 2 weeks prior loading of the organoids into the RoC. After 2 weeks, one organoid was placed in each well on top of the RPE-covered membrane. Hyaluronic acid-based hydrogel HyStem-C (ESI Bio, USA) was prepared according to manufacturer's recommendations and added into the wells. The medium volume inside each RoC well was brought to 27 μL with BRDM with 100 μM taurine and 10% FBS. During the RoC culture, the wells were sealed with a polymethyl methacrylate (PMMA) lid to avoid medium evaporation. BRDM with 100 μM taurine and 10% FBS was supplied at the constant flow rate of 40 μL/h using a syringe pump. Depending on the age of the ROs, the medium was additionally supplemented with RA, as mentioned above.

AAV vectors

The test articles used in this study are described previously.[45] Briefly, in AAV9.hCLN2, codon optimized human *TPP1* sequence is flanked by AAV2 inverted terminal repeats (ITRs) and the polyadenylation signal from the rabbit β-globin (RBG) gene. *TPP1* sequence is under the control of a CB7 promoter, a hybrid between a cytomegalovirus (CMV) immediate-early enhancer and the chicken β-actin promoter. The titer of the test article was determined by droplet digital PCR (ddPCR) using forward primer: 5'-TTC CCT CTG CCA AAA ATT ATG G-3′, reverse primer: 5'-CCT TTA TTA GCC AGA AGT.

AAV treatment

RO culture treatment

Young ROs (d88 and 123). On d0, one RO was placed in each well of non-adherent 96-well plates in 80 μL BRDM with 10% FBS and 100 μM taurine (depending on the age of the ROs, the medium was additionally supplemented with RA, as mentioned above). AAVs were thawed in ice and added via a 50% medium change to obtain a genome copy number of either 5x10^9, 5x10^{10} or 1.67x10^{11} gc/well. On d1, 20 μL of medium were added per well in order to have a final volume of 100 μL/well. The medium was changed twice per week by a 70% replacement. On d7 the medium volume was increased to 150 μL/well and on d35 the ROs were transferred to a 48-well plate with 300 μL culture medium. From d49, the ROs were cultured in 500 μL/well. After 35 days, the culture was stopped and the organoids were either fixed for immunohistochemical studies or snap frozen in liquid nitrogen for DNA or protein analysis. For the scRNA-seq analysis, the organoids were cultured for 70 days after treatment.

Matured ROs (d260). For matured organoids, the procedure was done similarly with slight modifications. On d1, the ROs were transferred to 48-well plates in 300 μL of culture medium. After 21 days, the culture was stopped and the organoids were collected and fixed.

RoC

Each well (containing one RO and RPE cells) received 6.5x10^9, 6.5x10^{10} or 2.167x10^{11} gc after organoid loading and hydrogel addition. The volume was adjusted to 27 μL with BRDM with 100 μM taurine and 10% FBS. Depending on the age of the ROs, the medium was additionally supplemented with RA, as mentioned above.

Comparison of RoC, 384 well plate and RoC with only RPE transduced

RoC were cultured as mentioned above but with modifications: RPE was cultured for 7 days prior to RO loading and the assembled RoC was cultured for 14 days. In parallel, RO were cultured in individual 384-well plate wells and filled with 23 μL BRDM +10% FBS +0.5μM RA. Medium was changed every other day (half medium change). To avoid evaporation, all other wells were filled with PBS and plates were packed in low density PE plastic bags. On d0, every well received dose 1 adjusted for the lack of RPE cells in comparison to the RoC (5.65 x10^9 gc/well). RoC received 6.5x10^9 gc/well. RoC with RPE only transfected were transduced on d1 after seeding the RPE (day −6) with dose 1 adjusted for only RPE (8.48x10^8) and only for 8 h. Afterward chips were washed once with BRDM and then cultured normally afterward.

RPEs

RPE cells were detached according to the procedure described previously[28] and 75,000 cells/cm^2 were seeded in 4-well culture inserts (ibidi, USA) placed into 35 mm µ-Dishes (ibidi, USA). RPE cells were cultured for 4 weeks in 100 µL/well BRDM. The medium was changed daily. After 4 weeks of culture, RPE cells received 10^5 or 10^{6}gc/cell diluted in 140 µL BRDM. On d1, medium was replaced with 100 µL/well BRDM. The medium was changed every day. After 9 weeks, cells were fixed for immunohistochemical studies.

CRISPR/Cas9 correction of *TPP1* mutation

To rescue TPP1 deficiency, CLN2 hiPSC lines were gently harvested with TrypLE at confluency of around 70–80%. 200,000 cells were centrifuged at 130 g for 5 min. The pellet was resuspended in 16,4 µL of Nucleofection solution and 3.6 µL of Nucleofection supplement (Amaxa P3 Primary Cell 4D-Nucleofector X Kit S, Lonza) containing 12 pmol S.p. Cas9-GFP V3 (IDT), 72 pmol sgRNA (Synthego) and 60 pmol ssDNA repair template for the (c.380G>A) mutation. The solution was transferred into the nucleocuvette and placed in the 4D-Nucleofector (Lonza). Cells were electroporated with the CA-137 pulse setting. 80 µL of mTESR+ (Stem Cell Technologies) with 10% CloneR (Stem Cell Technologies, USA) were gently added to the reaction and the cuvette was kept at 37°C for 3–5 min. After this short recovery phase, the cells were gently resuspended and transferred into a 96-well plate well coated with hESC-qualified Matrigel, where 120µL of mTESR+ with 10% CloneR had been pre-added. On the following day fresh mTESR+ containing 10% CloneR was added and the successful transfection was validated by GFP signal. On the following day the cells were harvested with TrypLE and 50 single cells were seeded into a hESC-qualified Matrigel coated 6-well plate well in mTESR+ with 10% CloneR. On day 1 and 3 the media was replaced with mTESR+ with 10% CloneR. The following media changes were performed with mTESR+. Around d10 the single cell colonies were manually picked under a microscope and seeded into a 96-well plate. The cell lines generated were genotyped to confirm the successful repair and then expanded, frozen and used for experiments. The sequence of all constructs and primers used for this study is reported in Table S3.

Overexpression of TPP1 cDNA and NMD inhibition

For overexpression experiments, TPP1 wildtype cDNA was commercially synthesized according to its ENSEMBL sequence (TPP1-201), as well as the mutations TPP1-c.379C>T and TPP1.c380G>A, by Eurofins genomics, Germany. cDNAs were then cloned into the pIRES2-AcGFP1 vector (Takara Bio, Japan). Overexpression was performed in commercial HEK 293T cells (LentiX, Takara Bio, Japan). For nonsense mediated decay inhibition, HEK 293T cells were pre-incubated for 2 h with 1µM NMDI14 (MedChemExpress, USA), transduced with plasmid vectors and subsequently treated for 24h until fixation of the cells.

Western Blot

Total protein was isolated from 10 organoids per condition using a chemical lysis method with RIPA buffer. The protein concentration in the samples was measured using the Pierce BCA Protein Assay Kit (Thermo Fisher Scientific, USA). Electrophoretic separation of proteins was performed on a 4–20% gradient Precast Gel System (Bio-Rad, USA), with 15 µg of total protein loaded per lane. Proteins were transferred to a nitrocellulose membrane using a tank blotting system. The membrane was blocked in Intercept Blocking Buffer (LI-COR, USA) for 1 h at room temperature, followed by incubation with primary antibodies against TPP1 and GAPDH for 1 h at room temperature. Afterward, the membrane was incubated with secondary antibodies for 1 h.

Immunohistochemistry

Immunofluorescence was performed either on fixed RPE cells or on organoid cryosections. ROs were collected and fixed in 4% PFA for 30 min at RT. Organoids were embedded in cryomolds using Tissue-Tek OCT (Sakura Finetek, USA) and stored at −80°C until further processing. Organoid cryosectioning was performed with a cryostat (14 µm slices, CM 3050 S Cryocut, Leica Biosystems, Germany), mounted on Superfrost microscope slides (Thermo Fisher Scientific, USA). Before staining, slides were rehydrated in PBS for 20 min and permeabilized with 0.5% Triton X- in PBS for 10 min. Then, they were incubated in blocking solution (10% donkey serum in PBS +0.2% Triton X-) for 1 h, with primary antibodies (Table S1; diluted in blocking solution) overnight at 4°C. Slice were then washed with PBS and incubated for 1 h at RT with Hoechst 33342 (1:1000, Thermo Fisher Scientific, USA) and secondary antibodies (Table S2; Abcam, UK; in 1:1 blocking solution:PBS). After each antibody incubation, antibody excess was removed by washing four times with PBS for 3 min. The samples were mounted with ProLong Gold Antifade Reagent without DAPI (Thermo Fisher Scientific, USA).

The RoCs were processed according to Achberger et al., 2021. Briefly, after disconnecting them from the syringe pumps, the RoCs were fixed with 4% PFA for 30 min at RT. Subsequently, the RoCs were washed with PBS and ROs were collected from RoCs by flushing wells with PBS and kept in 30% sucrose (in PBS) overnight. The RoCs with only RPE cells remaining were stored at 4°C. Embedding, cryosectioning and immunostaining were performed in the same way as retinal organoids.

RPE cells were fixed in 4% PFA for 20 min at RT. The staining procedure used for ROs was followed with minor modifications. RPE cells were permeabilized with 0.2% Saponin in PBS for 15 min and then blocked with 10% donkey serum in PBS +0.05% Saponin. Primary and secondary antibodies were diluted in blocking solution.

RO autofluorescence

Autofluorescent deposits were assessed on 14 μm sections of ROs at d350. Slides were viewed and photographed under a Stellaris 5 (Leica, Germany) confocal microscope equipped with a plan apochromatic 63× objective with glycerol immersion, whitelight laser with an excitation line set to 488 nm and Power HyD detectors. Emission detection was 501–584 nm. Images were pseudo-colored to magenta as emission wavelength.

Microscopy and image processing

All images were acquired by an Imager.M2 Apotome1 (Carl Zeiss, Germany), an Axio Imager Z1 (Zeiss, Germany) or a Stellaris 5 (Leica, Germany) microscope. Confocal images recorded with Stellaris 5 were acquired with 20x or 63× objective (Glycerol) at 1 airy unit (AU). If required, stitching was performed automatically by the Leica software. Images were exported as original TIFF and processed with ImageJ. Immunostaining images from main figures underwent noise reduction using ImageJ despeckle algorithm, as well as Supplementary figures Figures S1I, S1J, S3A–S3D, S5A and S5B.

Transmission electron microscopy and analysis

Retinal organoids were fixed in 2.5% glutaraldehyde, 2% paraformaldehyde, and 0.1 M sodium cacodylate buffer (pH 7.4, Electron Microscopy Sciences, Munich, Germany) overnight at 4°C. After rinsing in 0.1 M sodium cacodylate buffer, samples were postfixed in 1% OsO4 (Electron Microscopy Sciences) for 1.5 h at room temperature, washed in cacodylate buffer, and dehydrated with 50% ethanol. Tissues were counterstained with 6% uranyl acetate (Serva, Heidelberg, Germany) dissolved in 70% ethanol, followed by graded ethanol concentrations up to 100% and Propylenoxide. The dehydrated samples were incubated in 2:1, 1:1, and 1:2 mixtures of propylene oxide and Epon resin (Serva) for 1 h each. Finally, samples were infiltrated with pure Epon resin for 2 h. Samples were embedded in fresh Epon resin in block molds and cured for 3 days at 60°C. Semithin sections (500 nm) were cut on a Reichert Ultracut S (Leica Microsystems, Wetzlar, Germany) and stained with Richardson staining solution. Ultrathin sections (50 nm) were cut on the same Ultracut, collected on copper grids, and counterstained with Reynold's lead citrate. Sections were analyzed with a Zeiss EM 900 transmission electron microscope (Zeiss) equipped with a 2k x 2k CCD camera.

Curvilinear profiles were scored in at least 12 photoreceptor segments per cell line (12 segments from 3 different ROs were analyzed from CTRL1 and CLN2 lines; 16 segments from 4 different ROs were analyzed from CTRL2 and CLN1 lines). The area of the segments and the curvilinear profiles was calculated in ImageJ by defining regions of interest.

TPP1 quantification in supernatants

Supernatants were snap frozen until analysis and diluted in MSD Diluent 2 (MSD R51BB) before measurement. Total protein was measured by BCA assay (ThermoFisher 23235). TPP1 concentration was estimated by an electrochemiluminescence (ECL) immunoassay implemented using the Meso Scale Discovery (MSD) platform as described previously.[45] Briefly, biotinylated monoclonal anti-TPP1 antibody (R&D Systems no. MAB2237) was added to a streptavidin-coated MSD plate that has been blocked before use. After incubation, unbound antibody was washed from the plate followed by addition of samples, including calibration standards, quality controls (QCs), and study samples. After incubation, the plate was washed and any TPP1 protein captured by the immobilized antibody was detected by a polyclonal anti-TPP1 antibody (R&D Systems no. AF2237) labeled with SULFO-TAG. Following a wash step, the bound SULFO-TAG-labeled anti-TPP1 was detected with tripropylamine containing MSD read buffer. The intensity of the chemiluminescent signal, which was directly proportional to the amount of TPP1 present in the sample, was measured in an MSD reader.

Gene expression analysis via RT-qPCR

qPCR was performed using a direct 2-step approach with the QuantiFast SYBR Green RT-PCR Kit (Qiagen, Germany) according to the manufacture's instruction on a StepOnePlus real-time PCR system (Thermo Fisher Scientific, USA). The primer assays were: *GAPDH* (QT00079247, Qiagen, Germany) and *RCVRN* (QT00014098, Qiagen, Germany).

RNA sequencing

RNA was extracted using the Qiagen MicroRNeasy Kit. The concentration of RNA was measured using the Qubit Fluorometric Quantitation and RNA Broad-Range Assay (Thermo Fisher Scientific, USA). RNA Integrity Number RIN was determined using the Fragment Analyzer 5300 and the Fragment Analyzer RNA kit (Agilent Technologies, USA) and presented a good integrity (RIN>7). For library preparation, the mRNA fraction was enriched using polyA capture from 100ng of total RNA using the NEBNext Poly(A) mRNA Magnetic Isolation Module (NEB). Subsequently, libraries were prepared using the NEB Next Ultra II Directional RNA Library Prep Kit for Illumina and NEBNext UDI UMI (NEB, USA) following the manufacturer's instructions. To minimize technical batch effects, library preparations were performed using the liquid handler Biomek i7 (Beckman). The library molarity was determined by measuring the library size (approximately 400 bp) using the Fragment Analyzer 5300 and the Fragment Analyzer DNA HS NGS fragment kit (Agilent Technologies, USA) and the library concentration (>2 ng/μL) using Qubit Fluorometric Quantitation and dsDNA High sensitivity assay (Thermo Fisher Scientific, USA). The libraries were denaturated according to the manufacturer's instructions, diluted to 150 pM and sequenced as paired-end 100bp reads on an Illumina NovaSeqX (Illumina, USA).

10X genomics single-cell RNA sequencing

Two RO samples were used for each condition (CTRL1, CTRL2, CLN2-1, CLN2-2, AAV9.hCLN2-treated CLN2-1 and AAV9.hCLN2-treated CLN2-2). The ROs were dissociated with the Neurosphere Dissociation Kit (P) (Miltenyi Biotec, Germany) according to manufacturer's instruction. Then, the cells were filtered with a 30 μm MACS strainer (Miltenyi Biotec, Germany) and centrifuged for 2 min at 400 g. Subsequently, the cells were resuspended in proper volumes of 10% FBS in PBS for sequencing. All steps were performed at RT. Single cell suspension concentration and cell viability were determined by automatic cell counting (DeNovix CellDrop, DE, USA) using an AO/PI viability assay (DeNovix, DE, USA). Gene expression libraries were generated using the 10X Chromium Next gel beads-in-emulsion (GEM) Single Cell 3′ Reagent Kit v3.1 (10X Genomics, CA, USA) according to manufacturer's instructions. In brief, 18,000 cells originating from two different cell lines, were loaded on the Chromium Next GEM Chip G, which was subsequently run on the Chromium Controller (10X Genomics, CA, USA) to partition cells into GEMs. Cell lysis and reverse transcription of poly-adenylated mRNA occurred within the GEMs and resulted in cDNA with GEM-specific barcodes and transcript-specific unique molecular identifiers (UMIs). After breaking the emulsion, cDNA was amplified by PCR, enzymatically fragmented, end-repaired, extended with 3′ A-overhangs, and ligated to adapters. P5 and P7 sequences, as well as sample indices (Chromium i7 Multiplex kit, 10X Genomics, CA, USA), were added during the final PCR amplification step. The fragment size of the final libraries was determined using the Bioanalyzer High-Sensitivity DNA Kit (Agilent, CA, USA). Library concentration was determined using the Qubit dsDNA HS Assay Kit (Thermo Fisher Scientific, MA, USA). scRNA libraries were pooled and paired-end-sequenced on the Illumina NovaSeq 6000 platform using for the read 1 28 cycles, i7 index 10 cycles, i5 index 10 cycles and read 2 90 cycles.

QUANTIFICATION AND STATISTICAL ANALYSIS

Image analysis

All image analyses were performed with Fiji version of ImageJ 1.53 (https://imagej.net/software/fiji/). Quantification of fluorescent intensity of whole RO (10x epifluorescent images) was performed with a macro routine. Original TIFFs were used as input. Briefly, the macro semi-automatically recognized the area covered by the RO using thresholding the tissue background of one of the fluorescent or brightfield channels (manually adjustable by user). This ROI was then used for each channel to quantify average fluorescence using the "Measure" function of ImageJ. Background values were manually measured in a staining-negative area of one of the images of each channel. Subtraction of background values was then done using Microsoft Excel (Microsoft, USA).

Quantification of SCMAS, TPP1 and LipidSpot punctae (Figures 1I, S3H, S3I, 2A, 2E, 2F, 4J, 5D, S5C and S5D) in 63x stacked confocal images (0.33 μm distance between stacks at 1 AU) was performed using a self-written macro routine. Briefly, the macro allowed the user to select the area covered by the RO manually and then applies the "Smooth" algorithm of ImageJ. Then it used the 3D Object Counter Plugin with a manually determined threshold (the same threshold for each separate quantified channel was used) within the selected RO ROI. Statistics (Particle number, volume, position and mean intensity) were then saved in a csv table. Quantification was afterward performed using a self-written R script.

For co-localization analyses (Figures 2F and S3E–S3G), an adapted version of the above mentioned 63x quantification macro was used. First, 3D punctae were recognized for the SCMAS channel in the same way as above mentioned and saved in the 3D ROI manager. For the second channel (Recoverin, CRALBP, HSP60, TOMM20 or LAMP2) of the co-localization, a manual threshold was set by the user and the image was binarized (8-bit). Then, within all ROI of the SCMAS channel, the mean intensity of the second channel was calculated. The co-localization ratio of each image was then calculated as the mean intensity of the cell marker in all SCMAS punctae ROI divided by the image depth (255 for 8-bit).

For quantification of TPP1 and SCMAS fluorescent intensity in 2D RPE cultures, the ImageJ function "Measure" was used. Background values were manually measured in a staining-negative area of one of the images of each channel. Values were normalized on the fluorescent intensity in control ROs. For each replicate, 4 to 8 fields at 10× magnification or 11 to 18 fields at 20× magnification were analyzed.

Customized macros are available on Github (https://github.com/kachberger/Corti_et_al_TPP1).

RNA sequencing analysis

The sequencing aimed to achieve a depth of approximately >20 million clusters per sample. Read quality of RNA-seq data in fastq files was assessed using ngs-bits (2023_11-253-gc83b16dc), to identify sequencing cycles with low average quality, adaptor contamination, or repetitive sequences from PCR amplification. Reads were aligned using STAR v2.7.10b (Dobin et al.,2013) to the GRCh38 and alignment quality was analyzed using ngs-bits and visually inspected in the Integrative Genome Viewer (v2.15.4). Normalized read counts for all genes were obtained using Subread (v2.0.4). For differentiation gene expression analysis, raw counts filtered for protein coding genes were subjected to a standard DESeq2[76] pipeline. For Vulcano plot EnhancedVolcano 3.2 (https://github.com/kevinblighe/EnhancedVolcano) was used.

Gene ontology and semantic analysis

For gene ontology analysis (GO), the enrichGO function of the ClusterProfiler R package v4.6 (Yu et al., 2012)) with cellular component ontology, Benjamini-Hochberg as a p-adjustment method and q and p cutoff values of 0.2 was used. For visualization the dotplot

function of the ClusterProfiler package was used. For the venn plot, the github package ggvenn (https://github.com/NicolasH2/ggvenn) was applied. For the word cloud, GO terms were split into words and then frequency counted. Common words were removed by the stopwords function (https://cloud.r-project.org/web/packages/stopwords) as well as "cell" and "complex". Depiction was performed with the worcloud function (https://cran.r-project.org/web/packages/wordcloud/index.html) were used.

Single-cell sequencing data analysis

Samples were demultiplexed using Illumina's bcl2fastq conversion. A 10x Genomics custom reference package was created via Cell Ranger mkgtf v7.0.1 with the GENCODE GRCh38.p13 (release version 41) primary assemble reference sequence and main gene annotation file in GTF format along with the AAV vector genome sequence from the full-length 5′ flop ITR to the full-length 3′ flop ITR and the annotation of the transgene TPP1 sequence in the vector genome. Read alignments were performed in Cell Ranger count v7.0.1 against the pre-built reference package to generate gene-by-cell UMI count matrices with intronic reads included.

BAM files from cell ranger were used as input for sample demultiplexing and doublet removal by SNP genotyping using Souporcell v2.0.[72] Gene-barcode matrices were then loaded into Seurat (R, Version 4.3,[73]). Cells with less than 400 and more than the 98th percentile of detected genes, UMIs more than 98th percentile as well as more than 15% of mitochondrial genes were removed. Each Seurat element was then pre-processed separately (log-normalization, FindVariableFeatures (n = 2000), Scaling, PCA reduction) and then processed with the DoubletFinder R package v2.0.3.[77] Doublet rate was set to the expected multiplet rate of the 10X Genomics platform (10% for ~10000 cells recovered). Filtered datasets were then subjected to Seurat data integration[73] and merged. The combined dataset was then pre-processed again (log-normalization, scaling, PCA using the 2000 most variable features).

For UMAP reduction and for cluster identification (FindClusters), the first 35 pca dimensions were used. For clustering, the Louvain algorithm with a resolution of 0.4 was selected resulting in 16 clusters.

To identify each cluster, we used a list of marker genes (See Figure S2A) for each major retinal cell type (Retinal Progenitors, Rods, Cones, Bipolar Cells, Amacrine Cells, Horizontal Cells, Ganglion cells and Müler Glia). Each Louvain cluster was then module scored with the cell markers using the UCell Package v.2.2.0.[78] The highest scoring cell type name was then assigned to each cluster and same cell type clusters were merged for subsequent analysis. For comparison of only control and CLN2 RO lines, AAV9.hCLN2-treated RO samples were removed from the dataset by subsetting and re-processed as described above (Log-normalization, Scaling, PCA and UMAP reduction (first 35 PCA dimensions)). For visualizations, the Seurat (DimPlot, FeaturePlot, Violin Plot), ComplexHeatmap v.2.14.0 ([79], Heatmaps), Nebulosa v.1.8 (Density Plot,[80]) were used. Mean expression levels of TPP1 and AAV9.hCLN2 were calculated using the AverageExpression function of Seurat. The percentage of cells expressing *TPP1* and AAV9.hCLN2 were calculated with the Percent_Expressing function of scCustomize 1.1.1.[81]

Differential gene expression (DGE) analysis

For DGE analysis, the cone cluster of CTRL and CLN2 RO samples were subjected to the FindMarkers function from Seurat using the Wilcoxon test and Bonferroni correction. To identify top 25 up and downregulated genes, we further filtered the list for: minimum PCT (proportion of cells expressing a gene) of 0.2 for either CTRL or CLN2 samples, the same DGE tendency (up or downregulation) for each sample comparison (CTRL1 vs. CLN1, CTRL2 vs. CLN1, CTRL1 vs. CLN2, CTRL1 vs. CLN2), a log2FC > 0.2 and an adjusted p-value <0.05. The full list can be found in Table SX.

Gene set enrichment analysis (GSEA)

For GSEA analysis, log2FC values of the unfiltered DGE list from the cone cluster (Seurat's FindMarkers with a logf.threshold of -infinity and a min.pct of -infinity) were used as input into the gseGO function of the ClusterProfiler R package v4.6.[71] For reproducibility a seed was set before. The function was set as following: min Gssize = 3, maxGSize = 800, pvlaueCutoff = 0.05. All gene ontology sets (Biological Processes, Molecular Function and cellular components) were selected. For network visualization, the emapplot function was used. For selected GO Terms from the analysis, gene sets were retrieved using the getBM of the biomaRt R package v2.54.[82] GO terms that could not be retrieved via biomart were excluded from the analysis. The gene sets were then used for a Ucell module scoring of the single cell dataset. The median module score for the cone cluster of each cell line (CTRL1, CTRL2, CLN2-1, CLN2-2) was then visualized using ComplexHeatmap.

iRegulon analysis

For iRegulon Analysis the DGE list for cones (filtered for adjusted p-value <0.05) was entered into the iRegulon v1.3[74] plugin for Cytoscope v3.9.1 (https://cytoscape.org/). Enrichment score threshold was set at 2. AUC threshold at 0.03 and rank threshold at 5000. Data was visualized using R's ggplot2 v3.4.

Scoring of RICTOR targets obtained from Kline et al

The gene list obtained by Kline et al.[44] from the Sleat dataset[50] describing dysregulated genes regulated by RICTOR from CLN2 brain samples was used for Ucell scoring of the scRNAseq dataset. Scaled median values for each cell are depicted using the the ComplexHeatmap package.

QUANTIFICATION AND STATISTICAL ANALYSIS

RO culture treatment experiments were performed one to three times in the following way. Treatment at D88 (Figures 4G and 5A): CLN2-1 treated and untreated n = 9–10 ROs from two independent experiments; CLN2-2 treated and untreated n = 3–4 ROs from one experiment; CTRL1 *n* = 12 ROs from three independent experiments; CTRL2 *n* = 8 ROs from one experiment. Treatment at D123 (Figures 4H and 5B): CLN2-1 treated and untreated *n* = 10 ROs from two independent experiments; CLN2-2 treated and untreated n = 5–6 ROs from two independent experiments; CTRL1 *n* = 14 ROs from three independent experiments; CTRL2 *n* = 9 ROs from two independent experiments. Treatment at D260 (Figures 4I and 5C): CLN2-1 treated (dose 1 and 2) and untreated *n* = 11 ROs from two independent experiments; CLN2-1 treated (dose 3) *n* = 8 ROs from one experiment; CLN2-2 treated and untreated n = 5–8 ROs from two independent experiments; CTRL1 and 2 *n* = 9 ROs from two independent experiments.

RoC treatment (Figure 7) was performed in the following way. Each RoC is considered one independent experiment. CLN2-1 treated and untreated *n* = 10–11 ROs from 3 independent RoC; CLN2-2 treated and untreated *n* = 8 ROs from 2 independent RoC; CTRL1 *n* = 16 ROs from 4 independent RoC; CTRL2 *n* = 14 ROs from 4 independent RoC. RoC comparison to 384 well plates (Figure S7) were performed in the following way: each RoC is considered one independent experiment. CLN2-1 treated and untreated *n* = 4 ROs from 1 RoC to 5 384 wells; CLN2-2 treated and untreated *n* = 4 ROs from 1 RoC to 5 384-wells; CTRL1 *n* = 4 ROs from 1 RoC; CTRL2 *n* = 4 ROs from 1 RoC. CLN-2ISO *n* = 4 ROs from 1 RoC.

Statistical analysis was performed with GraphPad Prism 9.0 (GraphPad Software, USA). Statistical testing was performed using unpaired Student's T-test (Figures 3I and 3J), one-way ANOVA with Dunnett's post-hoc test (Figures 1H, 1I, 2A, 2B, 2E, 2F, 6A, S3E, S3F, S3G, S3H, S3I and S3M), one-way ANOVA with Bonferroni post-hoc test (Figures 5D, 6D, S5A, S5B, S5C and S5D), one-way ANOVA with Tukey post-hoc test (Figures S6K and S6L) two-way ANOVA with Dunnett's post-hoc test (Figures 4G–4I, 5A–5C, 7B and 7C), two-way ANOVA with Sidak post-hoc test (Figure S7B) and two-way ANOVA with Bonferroni post-hoc test (Figures 7E, 7F and 7G). For non-normal distributed samples (tested for normality with Kolmogorov-Smirnov), Kruskal-Wallis test with Dunn's post-hoc test was used (Figures 4J and 4K). *$p < 0.05$, **$p < 0.01$, ***$p < 0.001$, ****$p < 0.0001$.

Cell Reports Medicine, Volume *6*

Supplemental information

Recreating pathophysiology of CLN2 disease and demonstrating reversion by TPP1 gene therapy in hiPSC-derived retinal organoids and retina-on-chip

Serena Corti, Kwi Hye Kim, Ting Chen, Adelina Botezatu, Virginia Cora, Ke Ma, Natalia Pashkovskaia, Anamaria Bernal Vergara, Denise Sperlich, Kaushambee Dave, Arianna Tolone, Ryan M. Reddinger, Christopher B. Tully, Mikayla Higgins, Alexander Kleger, Markus Breunig, Paul Lopatta, Svenja Wingerter, Madalena Cipriano, Sylvia Bolz, Marius Ueffing, Nicholas Buss, Peter Loskill, Stefan Liebau, and Kevin Achberger

Supplemental Information- Recreating pathophysiology of CLN2 disease and demonstrating reversion by TPP1 gene therapy in hiPSCs-derived retinal organoid and retina-on-chip

Serena Corti[1*], Kwi Hye Kim[2*], Ting Chen[2], Adelina Botezatu[3,4], Virginia Cora[1], Ke Ma[1], Natalia Pashkovskaia[1], Anamaria Bernal Vergara[1], Denise Sperlich[1], Kaushambee Dave[1], Arianna Tolone[1], Ryan Reddinger[2], Christopher Tully[2], Mikayla Higgins[2], Alexander Kleger[6], Markus Breunig[6], Paul Lopatta[6], Svenja Wingerter[6], Madalena Cipriano[6], Sylvia Bolz[7], Marius Ueffing[7], Nicholas Buss[2], Peter Loskill[6,8], Stefan Liebau[1], Kevin Achberger[1†]

[1]Institute of Neuroanatomy & Developmental Biology (INDB), Eberhard Karls University Tübingen, Tübingen, Germany

[2]REGENXBIO Inc. Rockville, MD, USA

[3]German Cancer Consortium (DKTK), partner site Dresden, Germany

[4]German Cancer Research Center (DKFZ), Heidelberg, Germany

[5]Institute for Molecular Oncology and Stem Cell Biology, Ulm University Hospital, Ulm, Germany

[6]Institute of Biomedical Engineering, Eberhard Karls University Tübingen, Tübingen, Germany

[7]Centre for Ophthalmology, Institute for Ophthalmic Research, Eberhard Karls University Tübingen, Tübingen, Germany

[8]NMI Natural and Medical Sciences Institute at the University of Tübingen, Reutlingen, Germany

*Authors contributed equally

† Lead contact: kevin.achberger@uni-tuebingen.de

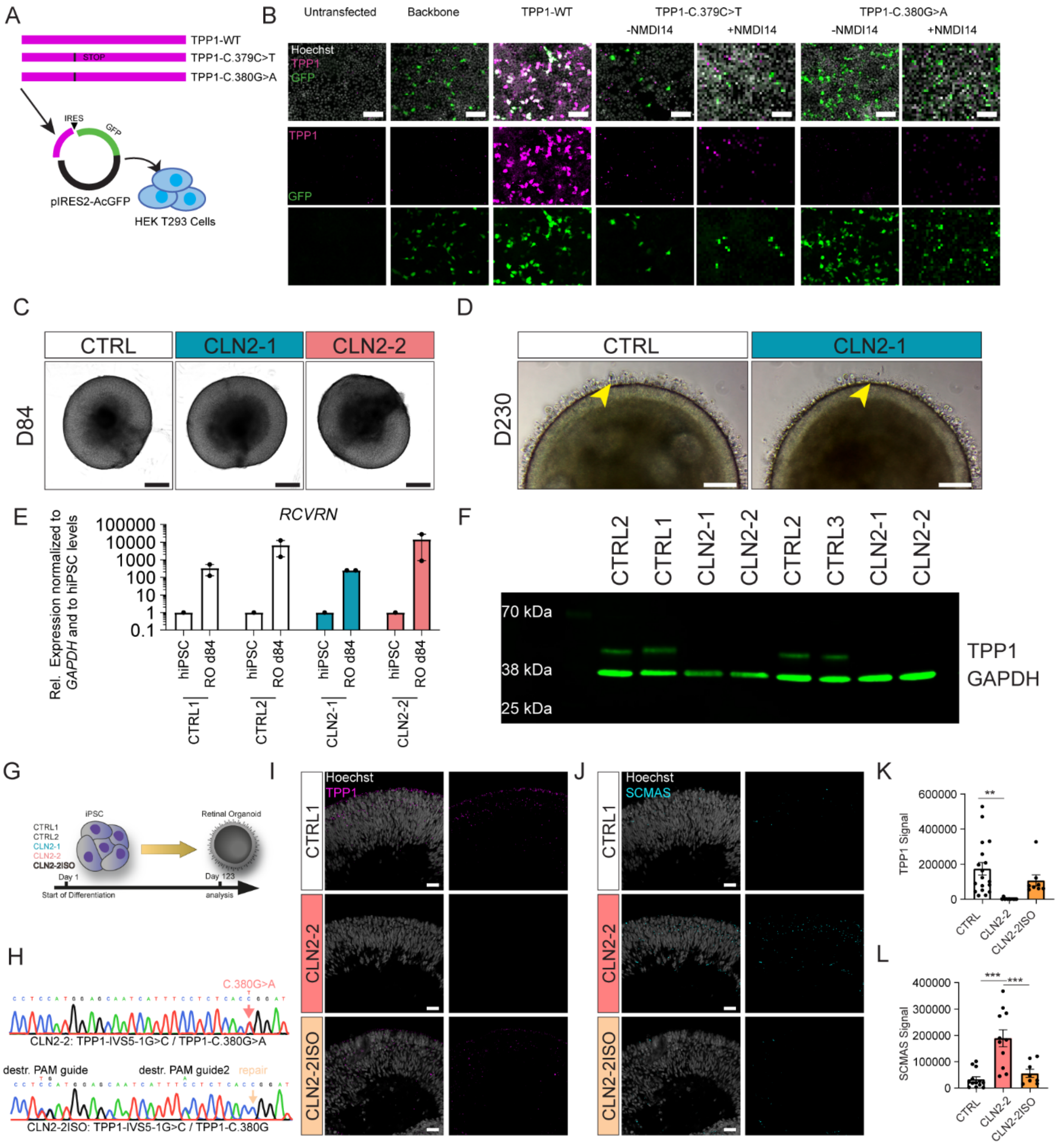

Fig. S1: Characterization of *TPP1* mutations and CLN2 Retinal Organoids. Related to Fig.1 and Fig.2

A) Experimental procedure: *TPP1*-wildtype (WT), *TPP1*-c.379C>T and *TPP1*-c380G>A cDNA was cloned into a pIRES2.AcGFP expression vector and then expressed in HEK T293 cells. B) *TPP1* cDNA variants were expressed for 24h in HEK T293 cells with or without 1 µM of nonsense mediated inhibitor NMDI14 and then stained for GFP (green) and for TPP1 (magenta). C) Brightfield image of CTRL1, CLN2-1 and CLN2-2 ROs at day 84 of differentiation. Scale bar = 200 µm. D) Brightfield image of photoreceptor outer segments (yellow arrowhead) in CTRL1 and CLN2-1 ROs at day 230 of differentiation. Scale bar = 100 µm. E) *RCVRN* (Recoverin) gene expression in ROs at day 84 of differentiation in comparison to hiPSC levels measured by qPCR. Values were normalized to the housekeeping gene GAPDH and then normalized to hiPSC levels. Y-axis is on log 10 scale. N= 1 hiPSC and 2 ROs from one differentiation. F) Western Blot analysis of TPP1 protein expression in CTRL1, CTRL2, CLN2-1, and CLN2-2 ROs at day 350. Samples from the first four and the last four lanes belong to two independent differentiation experiments. G) Creation of isogenic controls. H) Sanger Sequencing trace of mutated

(top) and corrected (bottom) allele in CLN-2ISO. I , J) TPP1 (magenta, I) and SCMAS (cyan, J) immunostaining of CTRL1, CLN2-2, and CLN2-2ISO ROs at day 123 of differentiation. Nuclei were counterstained with Hoechst (gray). Scale bar = 25 µm. K, L) TPP1 (K) and SCMAS (L) fluorescent intensity quantification in CTRL, CLN2-2 and CLN2-2ISO ROs at day 123. Values are mean ± SEM. N= 8-13 ROs from two differentiations. Statistical significance was evaluated by ordinary one-way ANOVA (***$p < 0.001$; ****$p < 0.0001$)

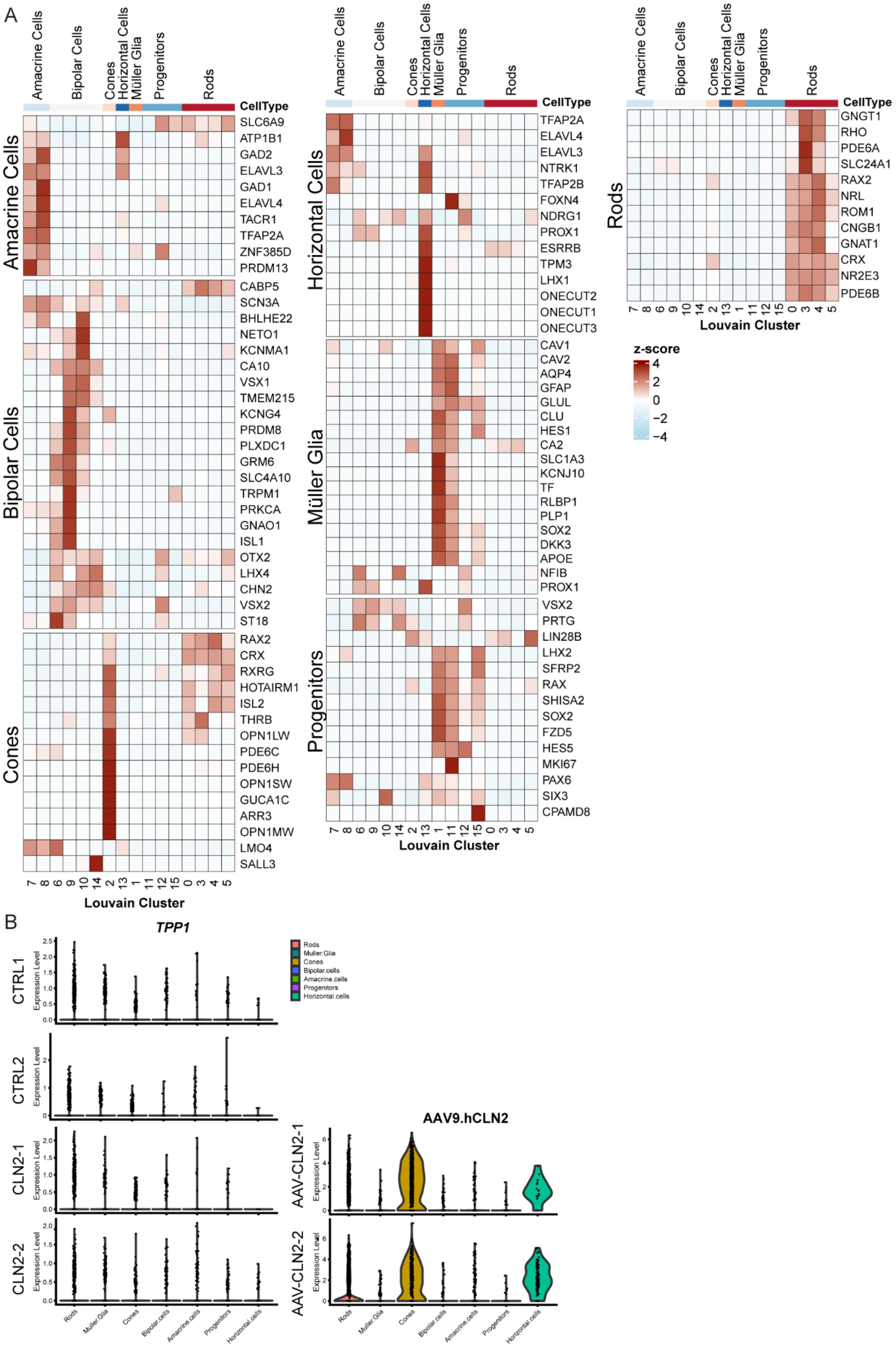
A
Amacrine Cells
Bipolar Cells
Cones
Horizontal Cells
Müller Glia
Progenitors
Rods
CellType
Amacrine Cells
SLC6A9
ATP1B1
GAD2
ELAVL3
GAD1
ELAVL4
TACR1
TFAP2A
ZNF385D
PRDM13
Bipolar Cells
CABP5
SCN3A
BHLHE22
NETO1
KCNMA1
CA10
VSX1
TMEM215
KCNG4
PRDM8
PLXDC1
GRM6
SLC4A10
TRPM1
PRKCA
GNAO1
ISL1
OTX2
LHX4
CHN2
VSX2
ST18
Cones
RAX2
CRX
RXRG
HOTAIRM1
ISL2
THRB
OPN1LW
PDE6C
PDE6H
OPN1SW
GUCA1C
ARR3
OPN1MW
LMO4
SALL3
7 8 6 9 10 14 2 13 1 11 12 15 0 3 4 5
Louvain Cluster
Horizontal Cells
TFAP2A
ELAVL4
ELAVL3
NTRK1
TFAP2B
FOXN4
NDRG1
PROX1
ESRRB
TPM3
LHX1
ONECUT2
ONECUT1
ONECUT3
Müller Glia
CAV1
CAV2
AQP4
GFAP
GLUL
CLU
HES1
CA2
SLC1A3
KCNJ10
TF
RLBP1
PLP1
SOX2
DKK3
APOE
NFIB
PROX1
Progenitors
VSX2
PRTG
LIN28B
LHX2
SFRP2
RAX
SHISA2
SOX2
FZD5
HES5
MKI67
PAX6
SIX3
CPAMD8
7 8 6 9 10 14 2 13 1 11 12 15 0 3 4 5
Louvain Cluster
Rods
GNGT1
RHO
PDE6A
SLC24A1
RAX2
NRL
ROM1
CNGB1
GNAT1
CRX
NR2E3
PDE6B
7 8 6 9 10 14 2 13 1 11 12 15 0 3 4 5
Louvain Cluster
z-score
4
2
0
−2
−4
B
TPP1
Rods
Muller.Glia
Cones
Bipolar.cells
Amacrine.cells
Progenitors
Horizontal.cells
CTRL1
CTRL2
CLN2-1
CLN2-2
Expression Level
AAV9.hCLN2
AAV-CLN2-1
AAV-CLN2-2
Rods
Muller.Glia
Cones
Bipolar.cells
Amacrine.cells
Progenitors
Horizontal.cells

Fig. S2. Retinal cell type identity, TPP1 and AAV9.hCLN2 transgene expression in CTRL and CLN2 ROs. Related to Fig.1

(A) Heatmap of a single cell RNA sequencing dataset of day 192 ROs (CTRL, CLN2 and AAV9.hCLN2-treated CLN2 ROs combined) depicting marker gene expression of 7 different retinal cell types (Amacrine Cells, Bipolar Cells, Cones, Horizontal Cells, Müller Glia, Retinal Progenitors and Rods) in unsupervised Louvain clusters (0-15). Values are depicted as scaled z-score. Top row indicates cell type label that was selected for each Louvain cluster. (B) Violin plots of a single-cell RNA sequencing dataset of day 192 ROs (CTRL1, CTRL2, CLN2-1, CLN2-2 and AAV9.hCLN2-treated CLN2 ROs) depicting *TPP1* gene and AAV9.hCLN2 transgene expression in 7 different retinal cell types (Rods, Müller Glia, Cones, Bipolar Cells, Amacrine Cells, Retinal Progenitors, and Horizontal Cells).

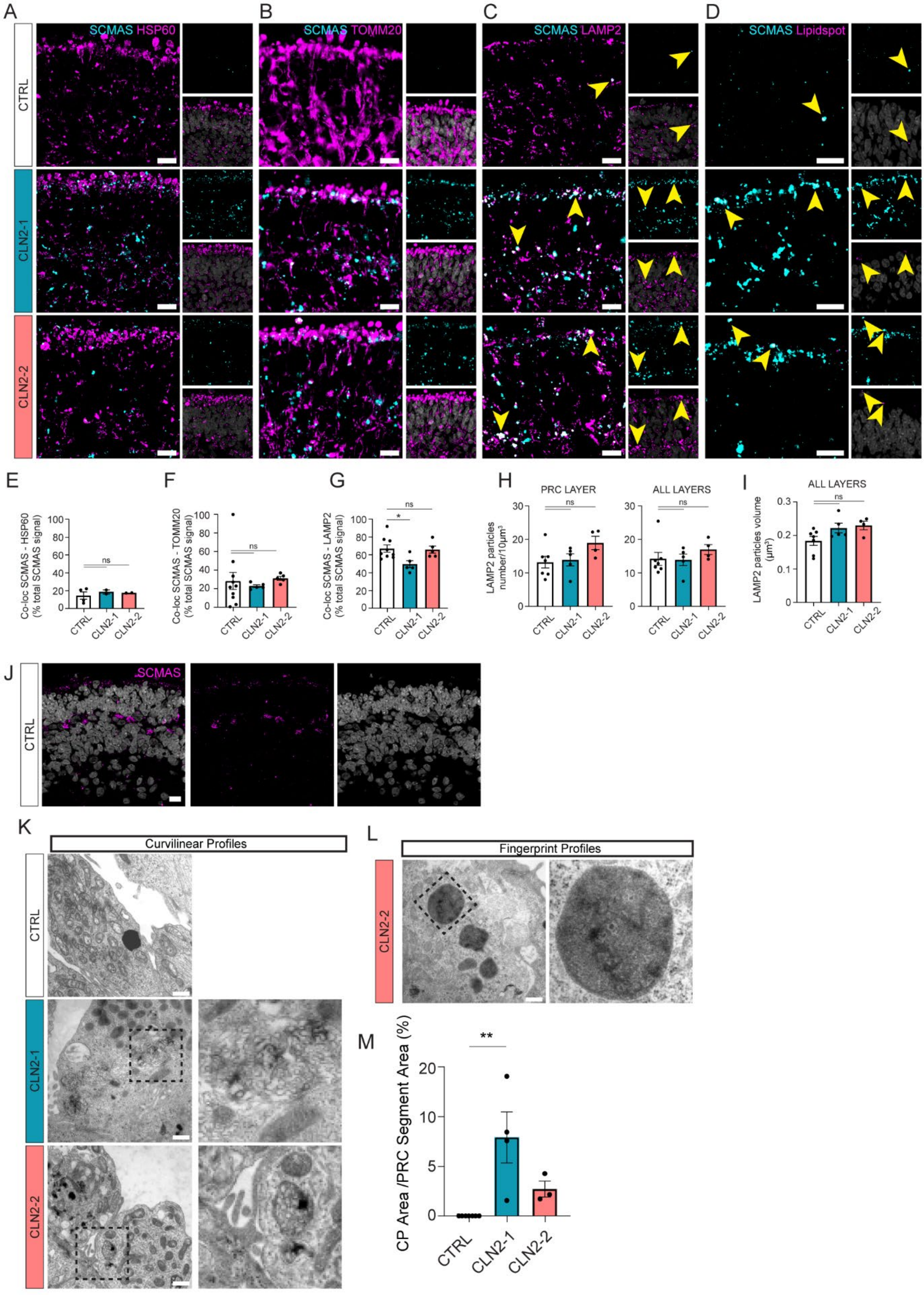

A
SCMAS HSP60
B
SCMAS TOMM20
C
SCMAS LAMP2
D
SCMAS Lipidspot
CTRL
CLN2-1
CLN2-2
E
Co-loc SCMAS - HSP60 (% total SCMAS signal)
F
Co-loc SCMAS - TOMM20 (% total SCMAS signal)
G
Co-loc SCMAS - LAMP2 (% total SCMAS signal)
H
PRC LAYER
ALL LAYERS
LAMP2 particles number/10µm³
I
ALL LAYERS
LAMP2 particles volume (µm³)
ns
*
J
SCMAS
K
Curvilinear Profiles
L
Fingerprint Profiles
M
CP Area /PRC Segment Area (%)
**

Fig. S3. Subcellular localization analysis of SCMAS punctae and ultrastructural evaluation of CLN2 depositions . Related to Fig.2

A-D) Single confocal plane showing co-localization of SCMAS (cyan) with HSP60 (magenta, A), TOMM20 (magenta, B) LAMP2 (magenta, C) and LipidSpot (magenta, D) in CTRL1 and CLN2 ROs at day 200 of differentiation. Nuclei were counterstained with Hoechst (gray). Scale bar = 10 µm. Examples of colocalizing signal are indicated with yellow arrowheads. E-G) Co-localization percentage of SCMAS with HSP60 (E), TOMM20 (F) and LAMP2 (G). Values indicate proportion of SCMAS signal that co-localizes with either HSP60 or LAMP2, respectively. N= 2-5 ROs from one differentiation. H and I) Quantification of LAMP2+ particles per 10 µm3 in photoreceptor cell (PRC) layer (H, left graph) and all retinal layers (H, right graph) and LAMP2+ particle volume (I) in CTRL and CLN2 ROs at day 200. Values are mean ± SEM. N=5 ROs from one differentiation. Statistical significance was evaluated by ordinary one-way ANOVA (*p < 0.05). J) Single confocal plane showing SCMAS expression visualized with increased brightness in a CTRL RO at day 158. K) Transmission electron microscopy images of d300 ROs showing curvilinear profiles (CP) in CLN2-1 and CLN2-2 photoreceptor (PRC) segments. The magnification of dashed squares is shown in the right column. Scale bar = 500nm. L) Transmission electron microscopy images of d300 CLN2-2 ROs showing fingerprint deposits in photoreceptor segments. The magnification of the dashed square is shown in the right column. Scale bar = 500nm. M) Percentage of photoreceptor segment area covered with curvilinear profiles in CTRL, CLN2-1 and CLN2-1 d300 ROs. Values are mean ± SEM. N= 3-5 ROs from one differentiation. Statistical significance was evaluated by ordinary One-Way ANOVA test (**p < 0.01)

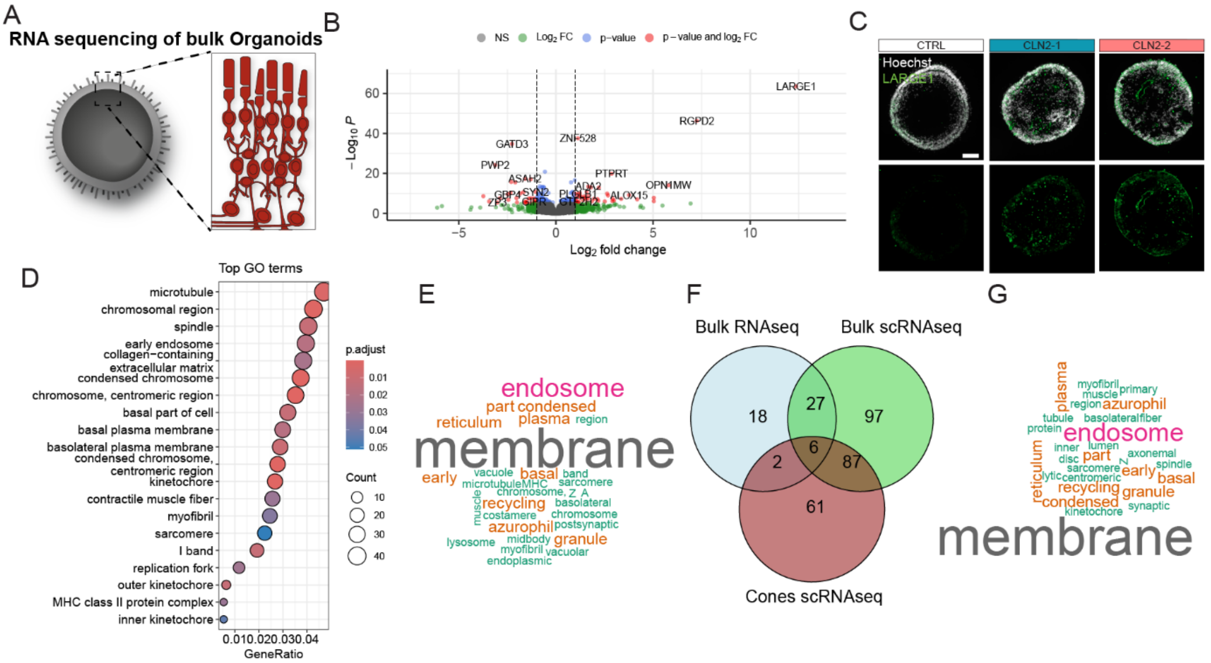

Fig. S4. Bulk RNA sequencing analysis. Related to Fig.3

A) RNA sequencing was performed with bulk d200 ROs. B) Vulcano Plot showing up- and downregulated genes comparing CLN2 (CLN2-1 and CLN2-2) and CTRLs (CTRL1 and CTRL2). Positive Log2 fold change values depict upregulation. C) Immunofluorescent staining showing LARGE1 (green) in CTRL2 and CLN2 organoids at day 321 of differentiation. Nuclei were counterstained with Hoechst (gray). Scale bar = 100 µm. D) Dot Plot of Gene Ontology (GO) Analysis showing the 20 top GO terms amongst dysregulated genes in CLN2 ROs in comparison to CTRLs. E) Word Cloud of semantic terms that were most mentioned in significantly enriched GO terms comparing CLN2 and CTRL ROs. F) Venn Diagram of GO Terms that have been found when using DGE of Bulk RNAseq, (Pseudo) bulk single cell RNAseq and scRNAseq cone data for the analysis. G) Word Cloud of semantic terms that are shared between bulk RNAseq and bulk scRNAseq analysis.

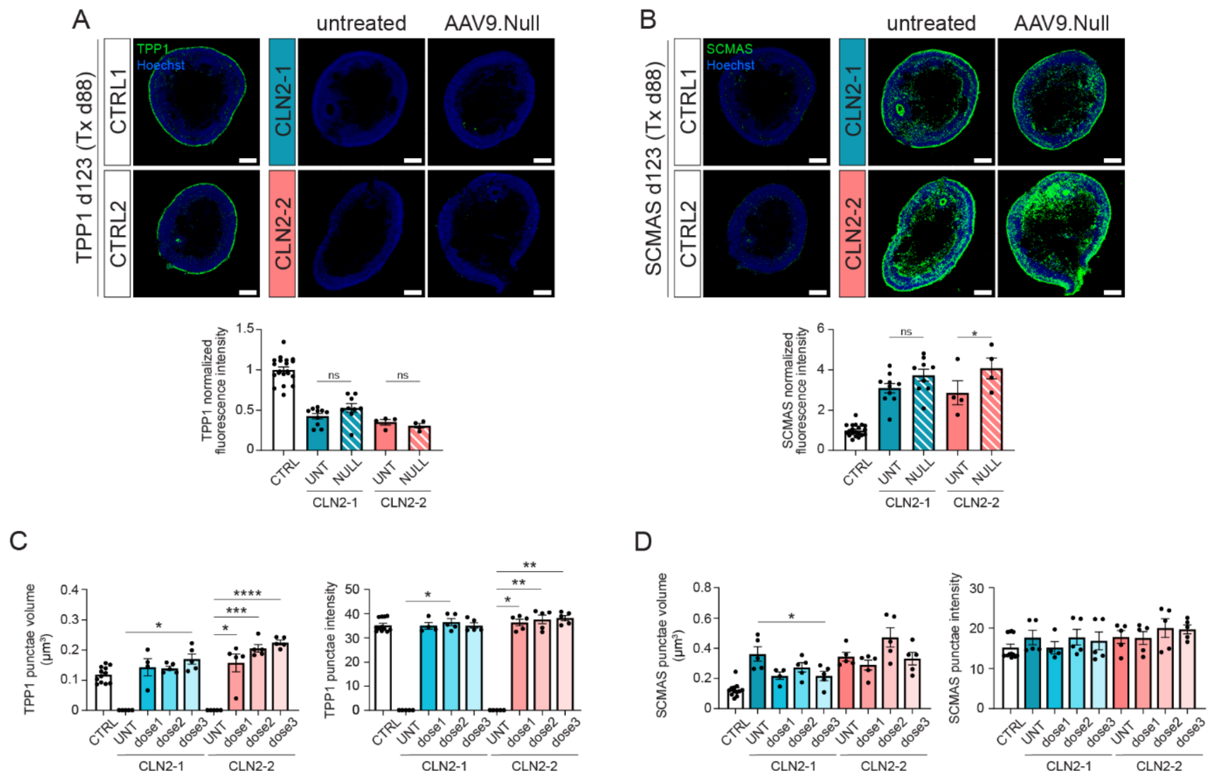

Fig. S5. Characterization of CLN2 ROs treated with AAV9.Null or AAV9.hCLN2. Related to Fig.4 and Fig.5

A and B) TPP1 (green, A) and SCMAS (green, B) immunostaining of d123 CTRL, untreated (UNT) CLN2 and CLN2 organoids treated with AAV9.Null (NULL) ($1.67x10^{11}$ gc/RO) at day 88. Nuclei were counterstained with Hoechst (blue). Scale bar = 100 µm. TPP1 and SCMAS protein expressions were evaluated by quantification of their fluorescent intensity. Number of analyzed organoids: CLN2-1 n=9-10 from two independent experiments; CLN2-2 n=4 from one experiment; CTRL1 n=8 from one experiment; CTRL2 n=12 from three independent experiments. Values are mean ± SEM. Statistical significance was evaluated by ordinary one-way ANOVA (*p < 0.05). Tx: treatment. C and D) Quantification of TPP1 (C) and SCMAS (D) punctae volume (left graphs) and fluorescent intensity (right graphs) in CTRL and CLN2 d158 ROs treated with AAV9.hCLN2 at day 123. Three different doses of AAV9.hCLN2 were tested (dose 1: $5x10^9$, dose 2: $5x10^{10}$ and dose 3: $1.67x10^{11}$ gc/RO). Values are mean ± SEM. N=5 organoids from two independent experiments. Statistical significance was evaluated by ordinary one-way ANOVA (*p < 0.05; ***p < 0.001; ****p < 0.0001)

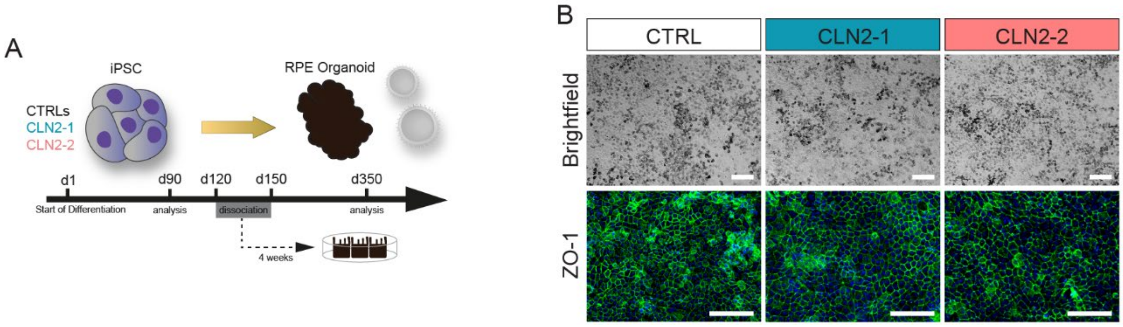

Fig. S6. Characterization of RPE cells. Related to Fig.6

A) Schematic overview of the 2D RPE derived from 3D RPE organoids differentiation protocol and time points used for analyses. B) Brightfield images (top row) and immunostaining of the RPE marker ZO-1 (green, bottom row) of CTRL1 and CLN2 RPE cells cultured in 2D for 4 weeks. Nuclei were counterstained with Hoechst (blue). Scale bar = 100 µm.

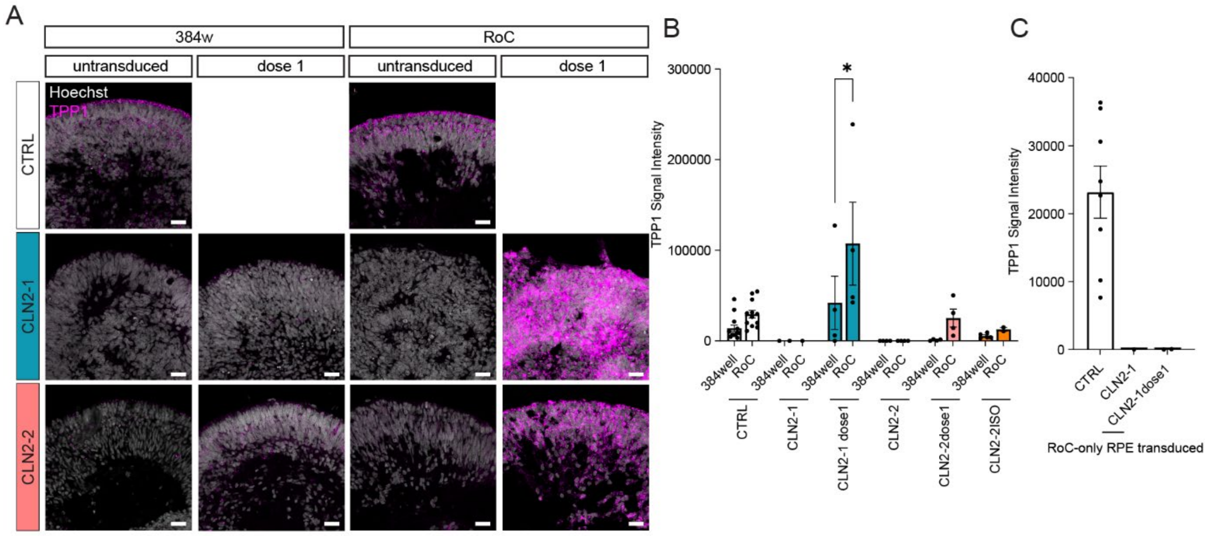

Fig S7: AAV9.hCLN2 is more efficient in the RoC than in 384-well plate. Related to Fig.7

A and B) Comparison of the RO transduced with AAV9-hCLN2 (dose 1) in a 384-well plate and the RoC with similar volumes. A) Exemplary TPP1 (magenta) immunostaining of CTRL1, CLN2-1 and CLN2-2 ROs at day 123 of differentiation comparing 384-well plate and RoC cultures as well as untransduced and AAV9.hCLN2 transduced (dose 1). Nuclei were counterstained with Hoechst (gray). Scale bar = 25 µm. B) TPP1 protein was evaluated by quantification of fluorescent intensity. N=4 from one experiment. Values are mean ± SEM. Statistical significance was evaluated by a two-way ANOVA (**p < 0.01). C) TPP1 protein levels of a RoC, where only the RPE cell were transduced with AAV9-hCLN2 ("RoC-only RPE transduced") at dose 1. Values are mean ± SEM. N=4 from one experiment.

Table S1, related to STAR Methods- Immunohistochemistry

Antibody/dye	Species/type	Dilution	Catalog number	Supplier
CRALBP	Mouse monoclonal	1:250	ab15051	Abcam, UK
Ezrin	Rabbit polyclonal	1:200	3145	Cell Signaling Technology, USA
GAPDH	Mouse monoclonal	1:1000	MAB374	Merck Millipore, USA
GFP	Rabbit polyclonal	1:1000	A6455	Thermo Fisher Scientific, USA
HSP60	Mouse monoclonal	1:1000	ab128567	Abcam, UK
LAMP1	Rabbit polyclonal	1:200	ab24170-100	Abcam, UK
LAMP2	Mouse monoclonal	1:50	Sc-18822	Santa Cruz Biotechnology, USA
LipidSpot™ 488 Lipid Droplet Stain	-	1:1000	70065-T	Biotium, USA
Recoverin	Goat polyclonal	1:50	Sc-20353	Santa Cruz Biotechnology, USA
TPP1	Mouse monoclonal	1:200	Sc-365838	Santa Cruz Biotechnology, USA
SCMAS	Rabbit polyclonal	1:100	ab181243	Abcam, UK
TOMM20	Rabbit polyclonal	1:100	11802-1-AP	Proteintech,USA
TOMM20	Mouse monoclonal	1:200	SC-17764	Santa Cruz Biotechnology, USA

ZO1	Mouse monoclonal	1:100	33-9100	Thermo Fisher Scientific, USA

Table S2, related to STAR Methods- Immunohistochemistry

Antibody	Type	Dilution	Catalog number	Supplier
Alexa Fluor™ 488	Donkey anti mouse IgG	1:500	ab150105	Abcam, UK
Alexa Fluor™ 488	Donkey anti rabbit IgG	1:500	ab150073	Abcam, UK
Alexa Fluor™ 568	Donkey anti mouse IgG	1:500	ab175472	Abcam, UK
Alexa Fluor™ 568	Donkey anti rabbit IgG	1:500	ab175470	Abcam, UK
Alexa Fluor™ 647	Donkey anti mouse IgG	1:500	ab150107	Abcam, UK
Alexa Fluor™ 647	Donkey anti rabbit IgG	1:500	ab150075	Abcam, UK
Alexa Fluor™ 647	Donkey anti goat IgG	1:500	ab150131	Abcam, UK
Alexa Fluor™ 568	Donkey anti rabbit IgG	1:500	A10042	Thermo Fisher Scientific, USA

Table S3, related to STAR Methods- CRISPR/Cas9 correction of TPP1 mutation

	**Sequence**
sgRNA	GAGAGGAAAUGAUUGCUCCA
Repair Template	ccatccatctcactgatgggatgactggtgccctTGatggagcaatcatttActctcacCGgatgctc agccagcaagtcagaaagtcctgtgtgatcac
Fwd primer for sequencing	ACATGGGTTTCCGTAGGTCC
Rvrs primer for sequencing	TGCCTCTTCTGAATCCCTGC
